# Supplementary figures and images for: Inhibition of mitochondrial protein import and proteostasis by a pro-apoptotic lipid
Source: eLife. 2025 May 30;13:RP93621. doi: 10.7554/eLife.93621 (PMC12124835; doi:10.7554/eLife.93621)

Figure 4D

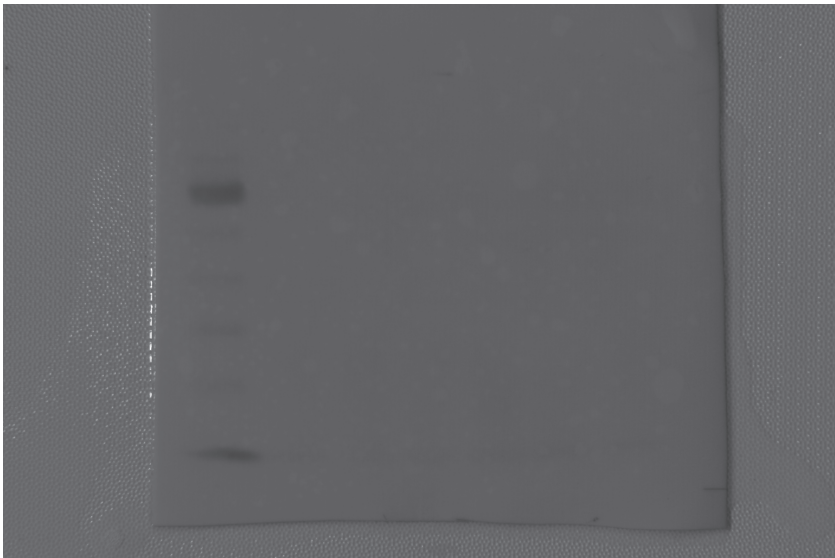

Protein ladder

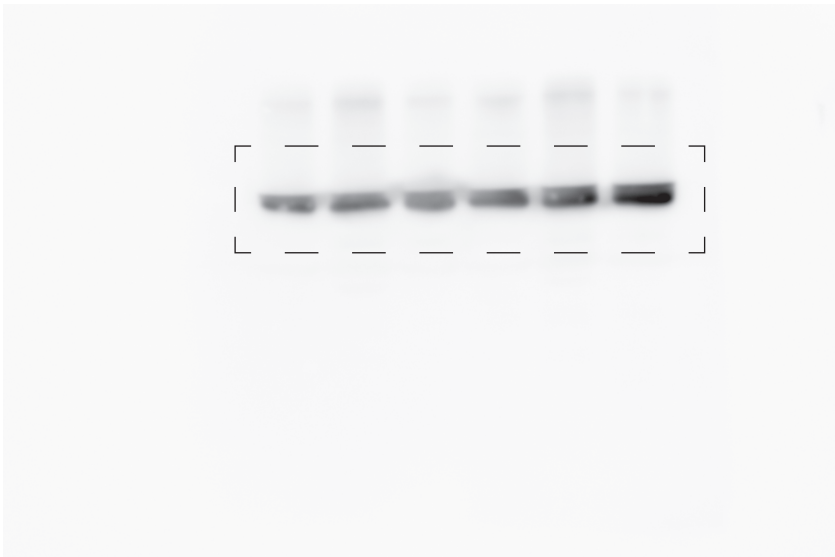

Relevant bands

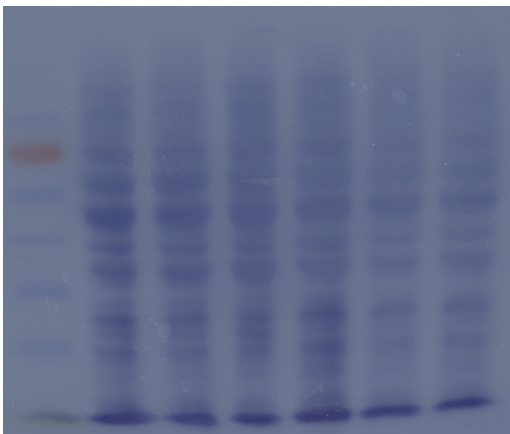

DB71 stain

Supplement: Figure 4—source data 1. [file elife-93621-fig4-data1.pdf]

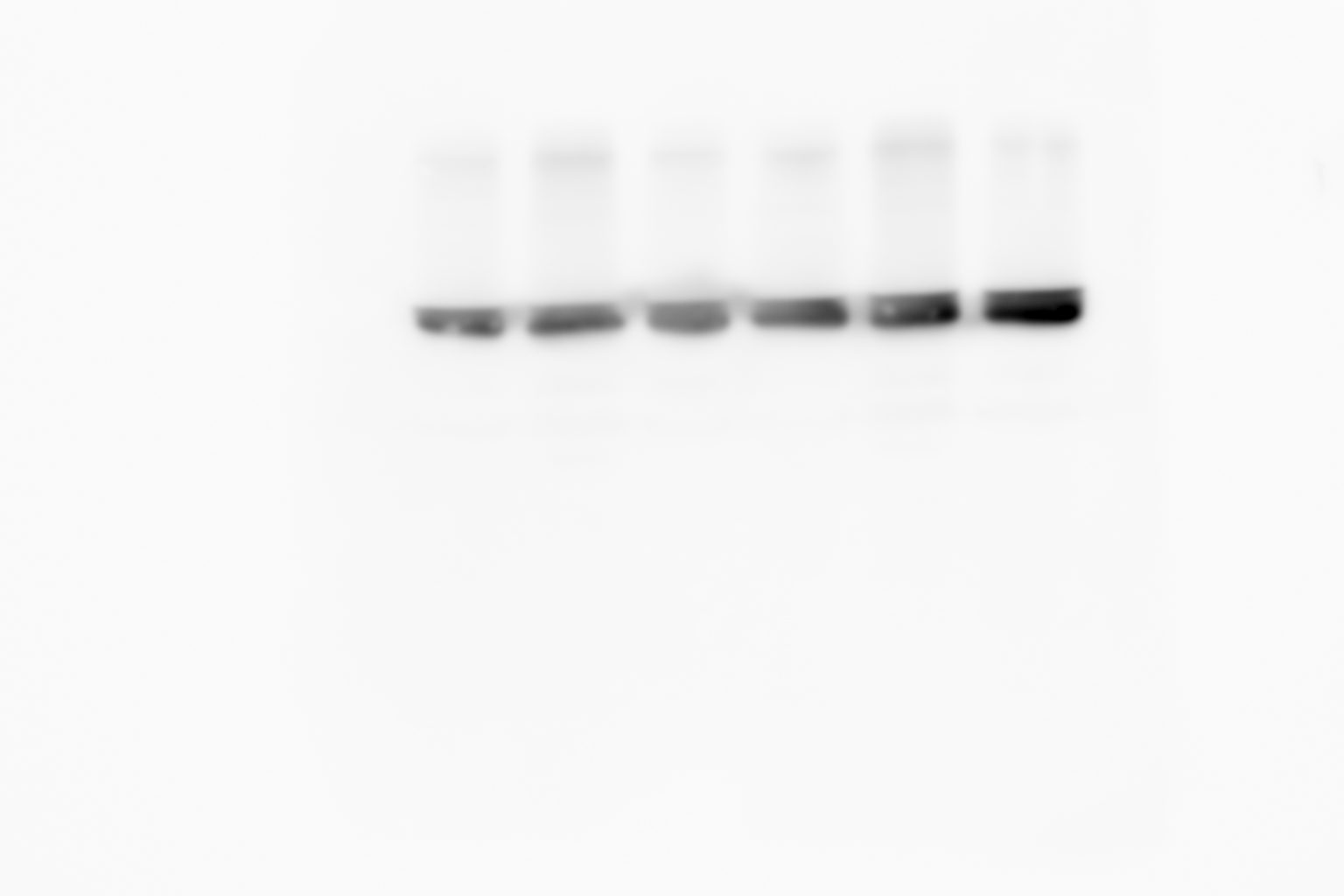

Supplement: Figure 4—source data 2. [file elife-93621-fig4-data2.zip › Figure 4D_Bands.tif]

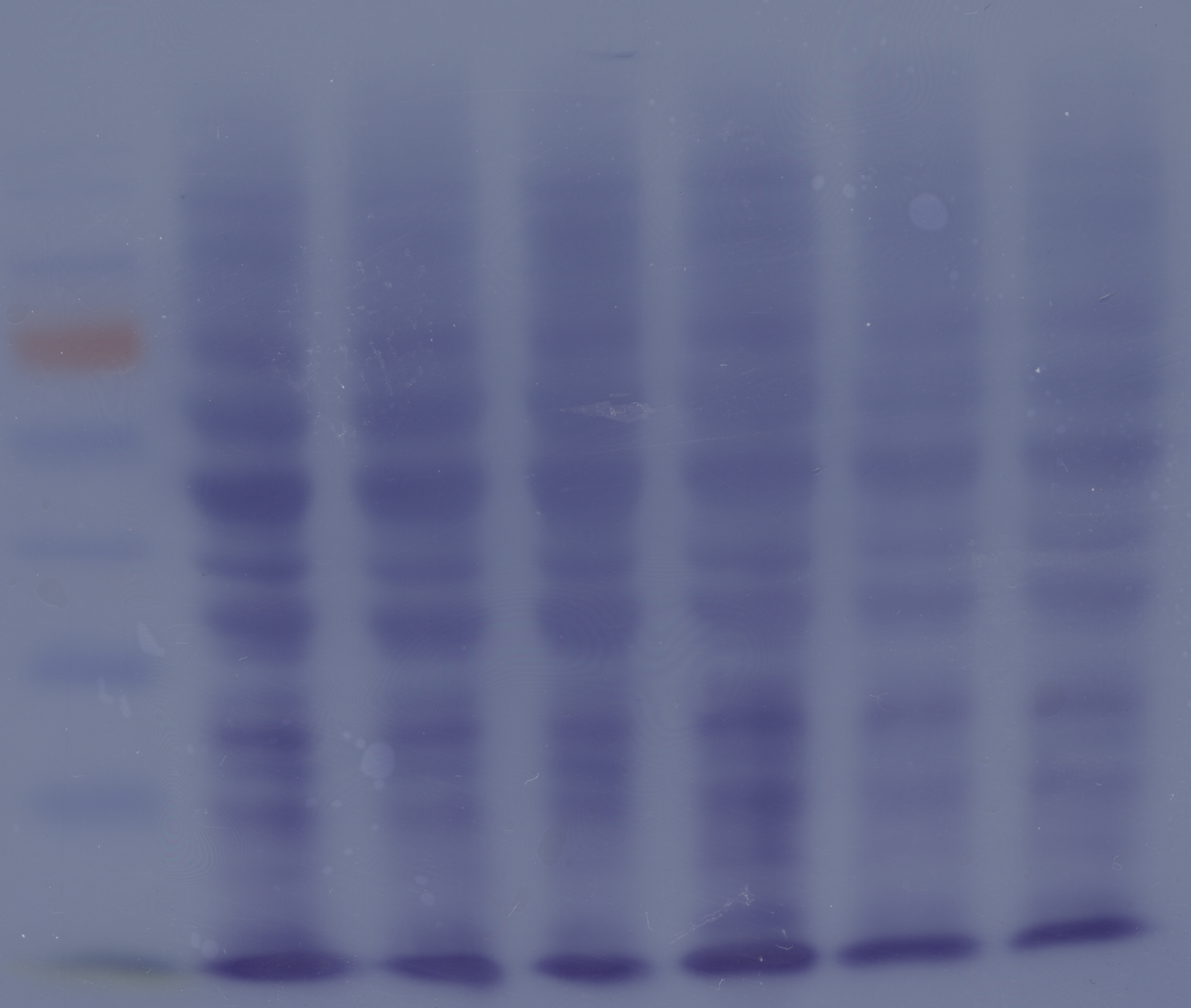

Supplement: Figure 4—source data 2. [file elife-93621-fig4-data2.zip › Figure 4D_DB71.tif]

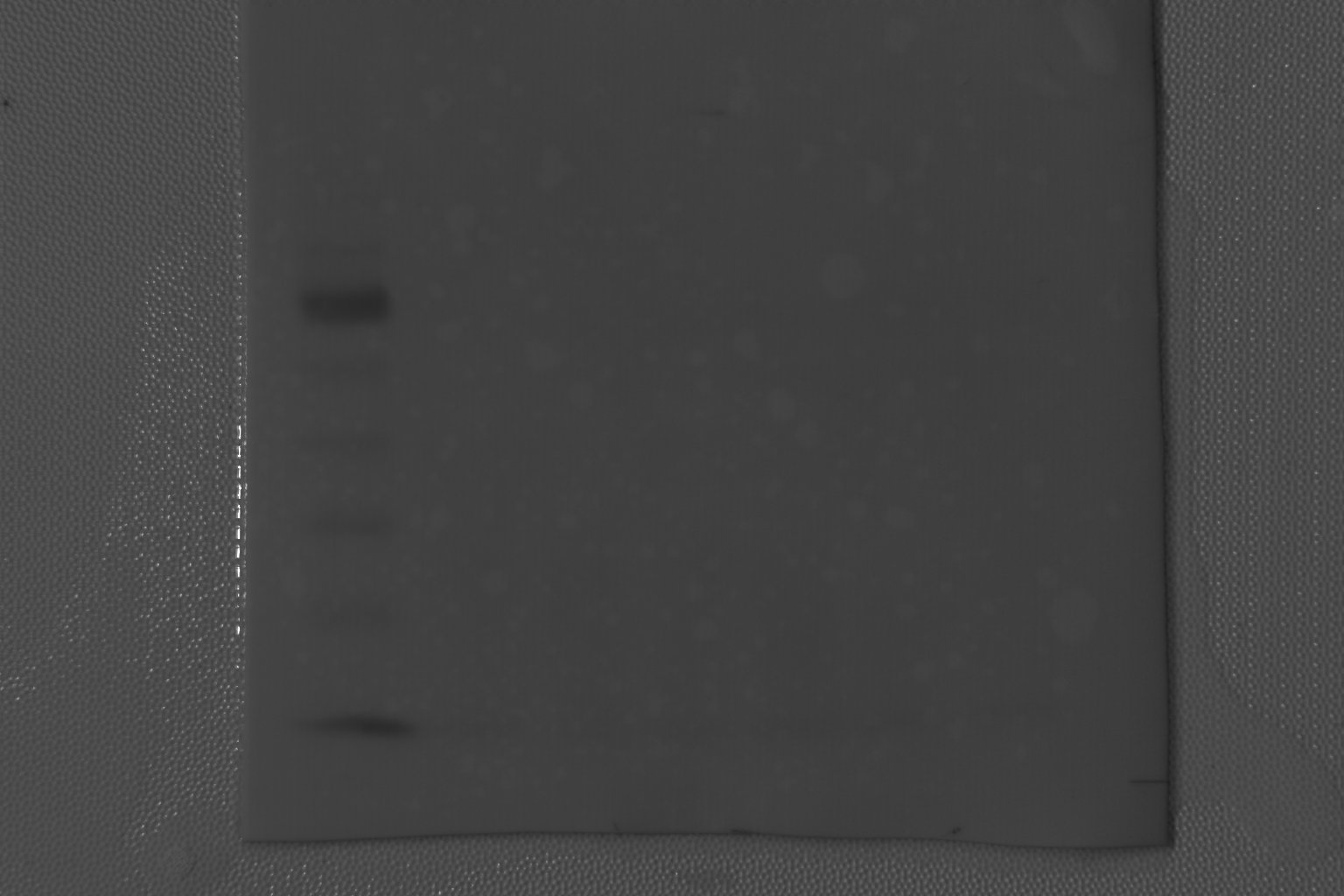

Supplement: Figure 4—source data 2. [file elife-93621-fig4-data2.zip › Figure 4D_Protein ladder.tif]

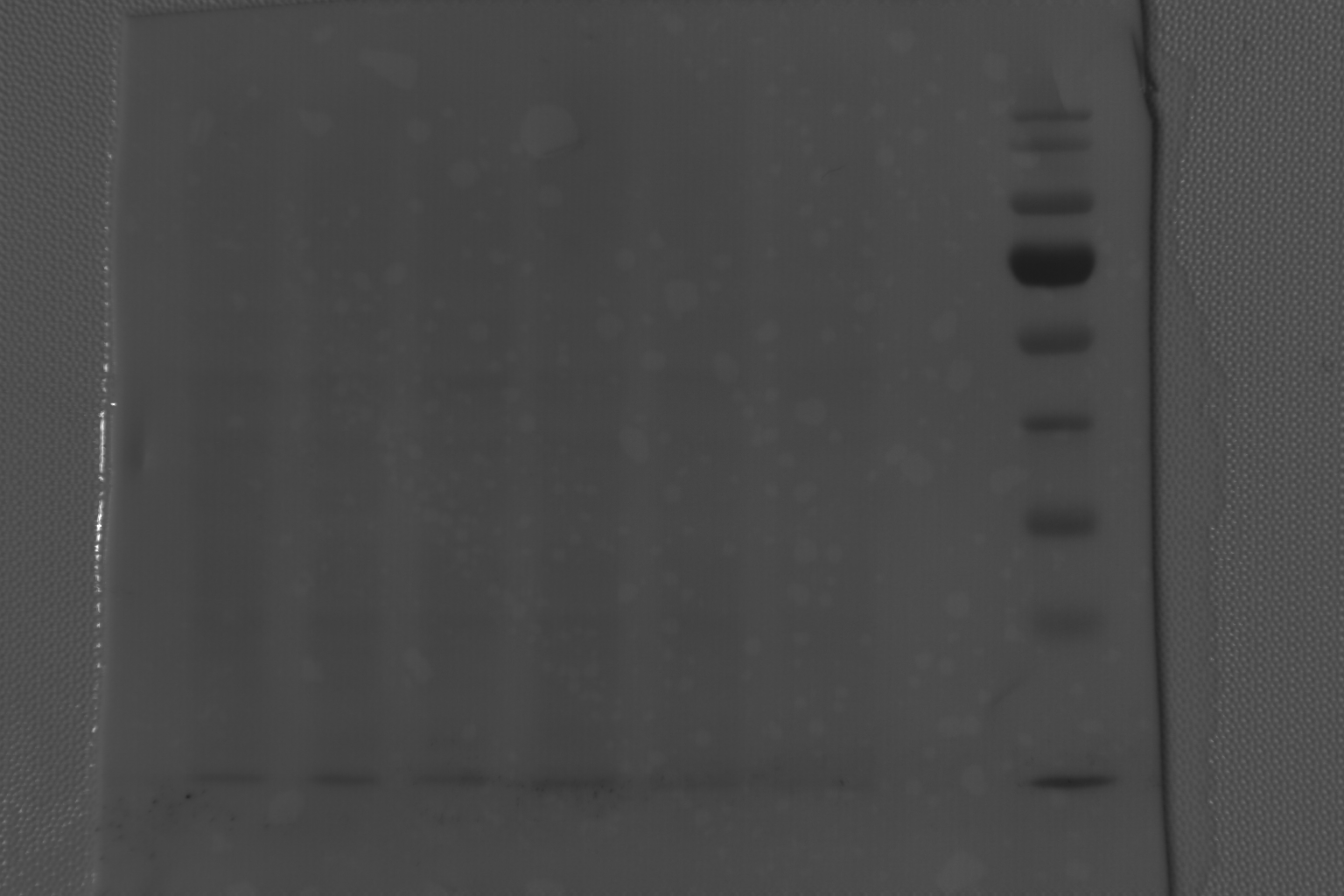

Supplement: Figure 5—source data 2. [file elife-93621-fig5-data2.zip › Figure 5D-Aim17_Protein ladder.tif]

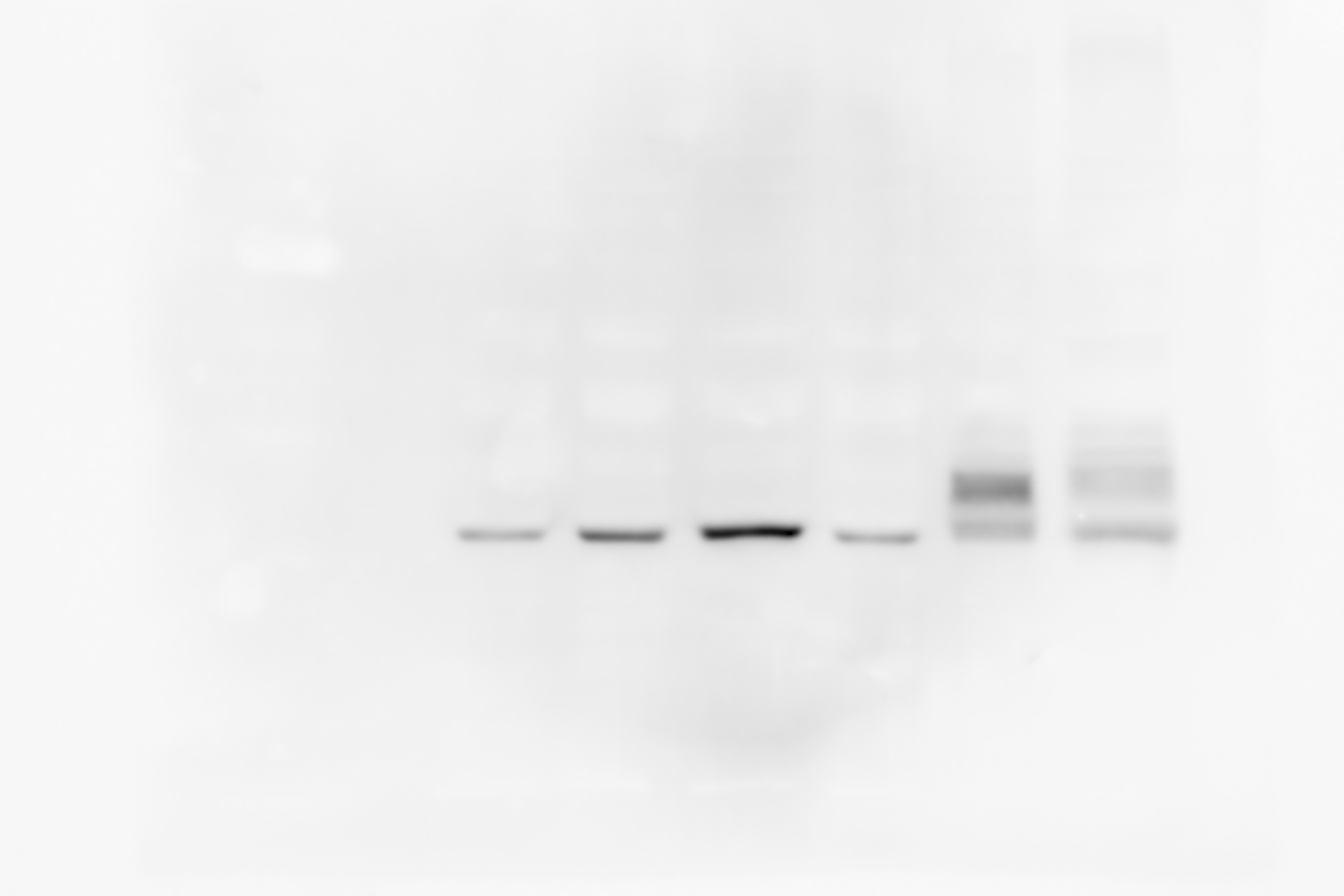

Supplement: Figure 5—source data 2. [file elife-93621-fig5-data2.zip › Figure 5D-Cyc7_Bands.tif]

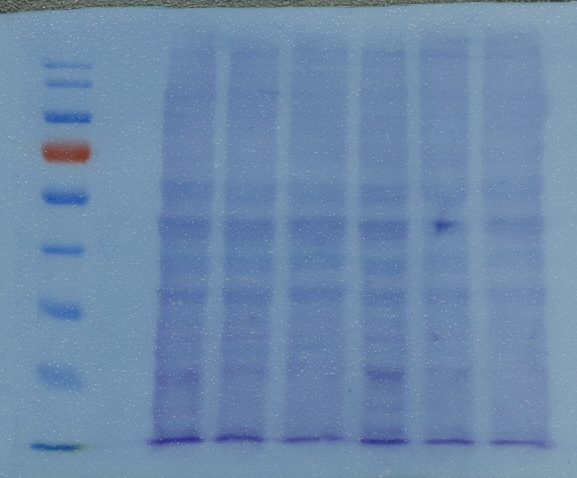

Supplement: Figure 5—source data 2. [file elife-93621-fig5-data2.zip › Figure 5D-Cyc7_DB71.jpg]

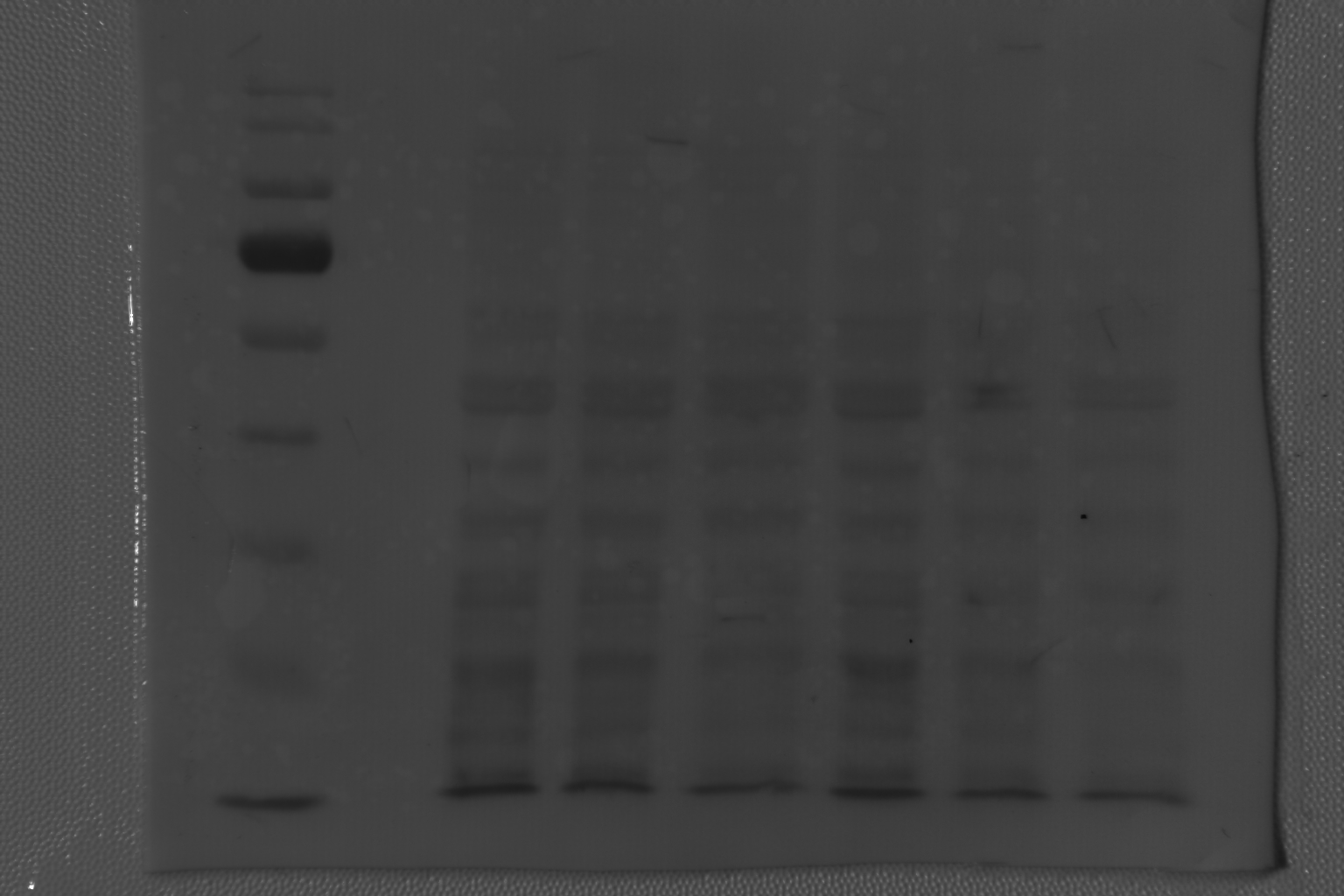

Supplement: Figure 5—source data 2. [file elife-93621-fig5-data2.zip › Figure 5D-Cyc7-Protein ladder.tif]

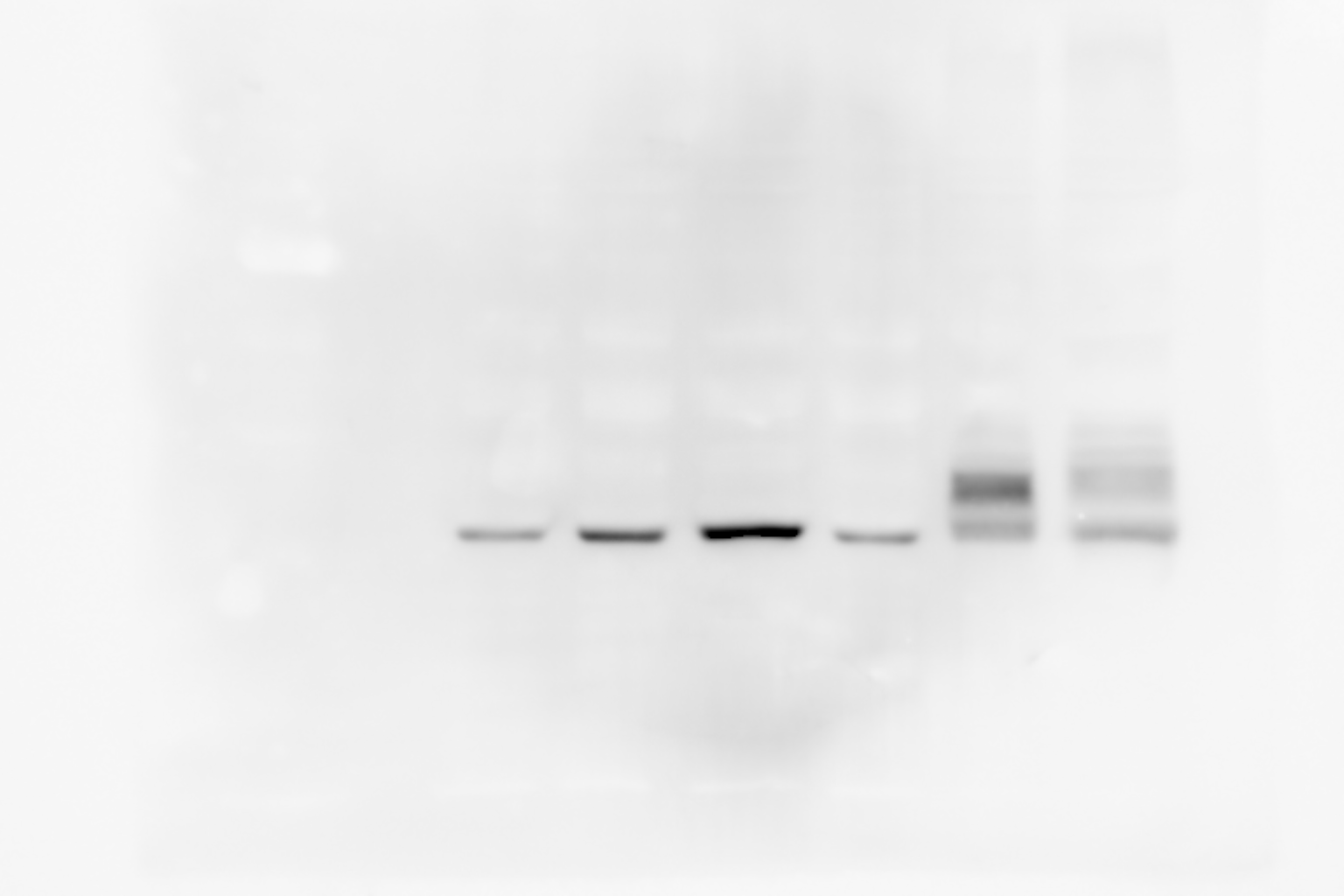

Supplement: Figure 5—source data 2. [file elife-93621-fig5-data2.zip › Figure 5D-Mpc3_Bands.tif]

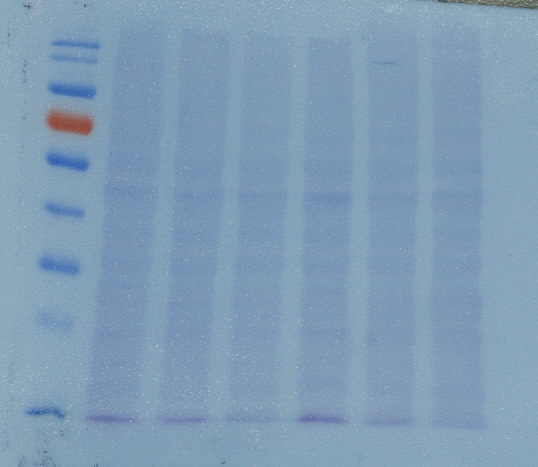

Supplement: Figure 5—source data 2. [file elife-93621-fig5-data2.zip › Figure 5D-Mpc3_DB71.jpg]

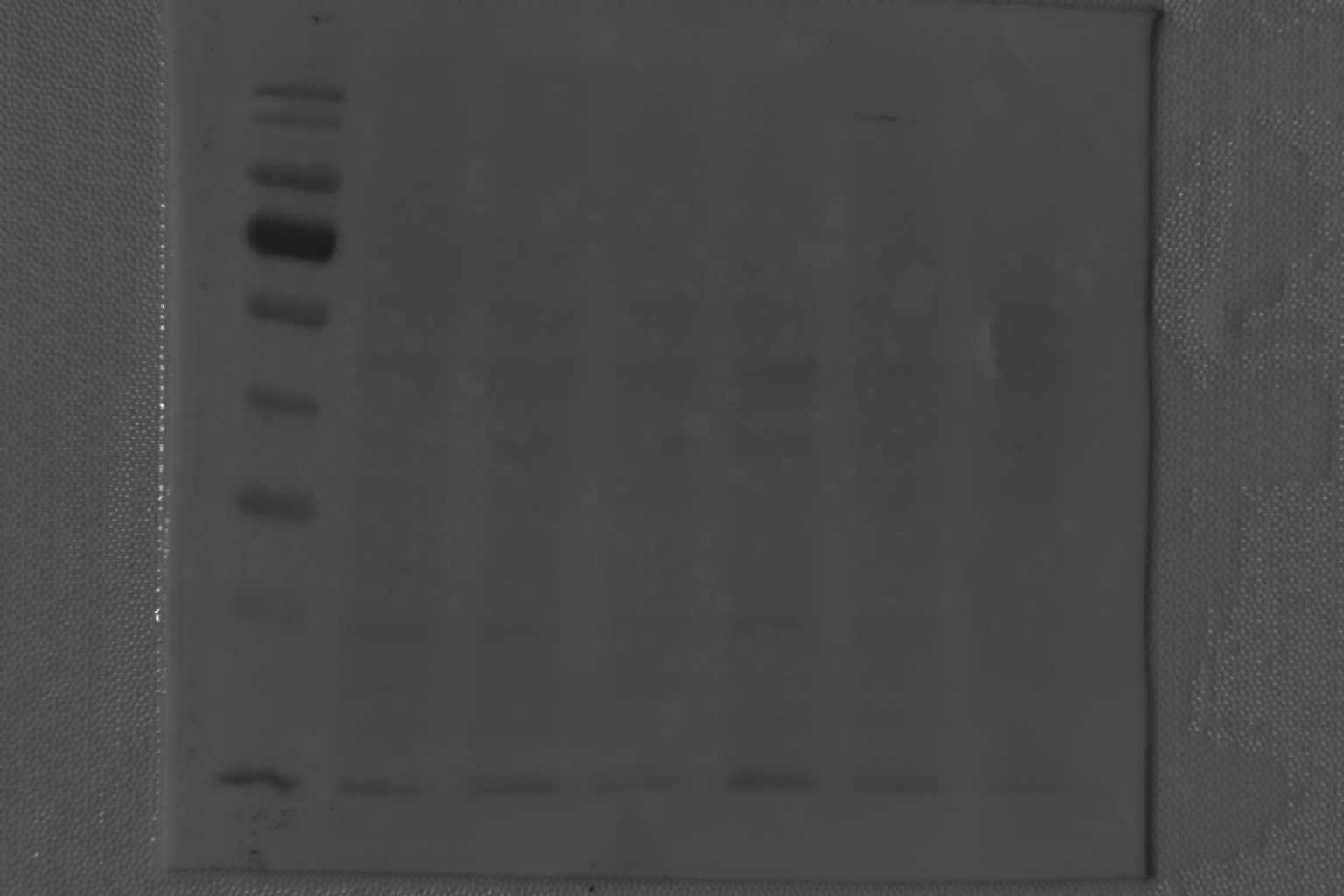

Supplement: Figure 5—source data 2. [file elife-93621-fig5-data2.zip › Figure 5D-Mpc3_Protein ladder.tif]

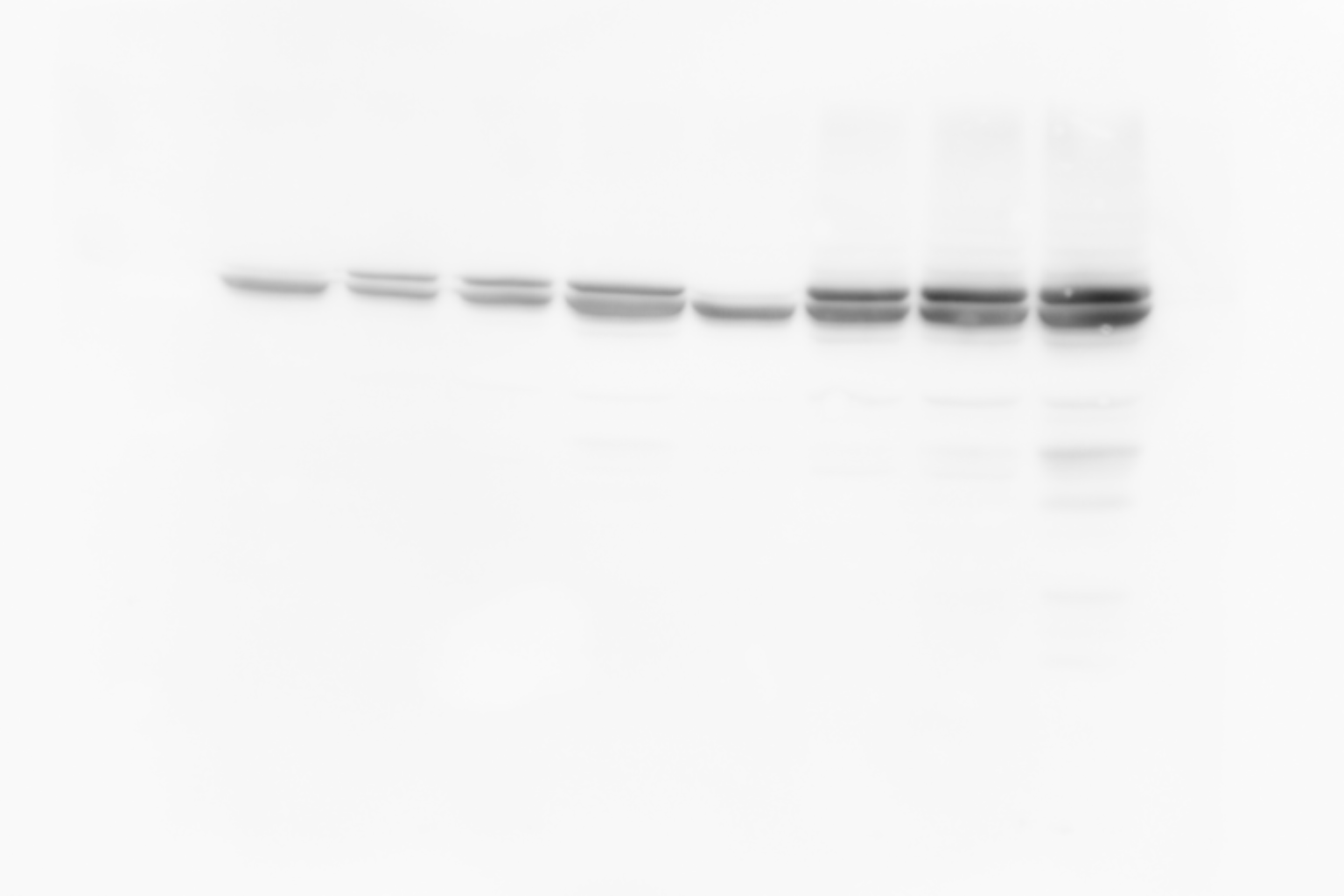

Supplement: Figure 5—source data 2. [file elife-93621-fig5-data2.zip › Figure 5A-Aim17_Bands.tif]

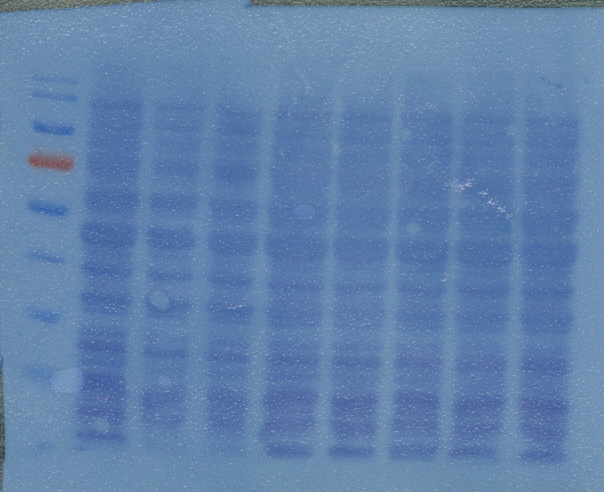

Supplement: Figure 5—source data 2. [file elife-93621-fig5-data2.zip › Figure 5A-Aim17_DB71.jpg]

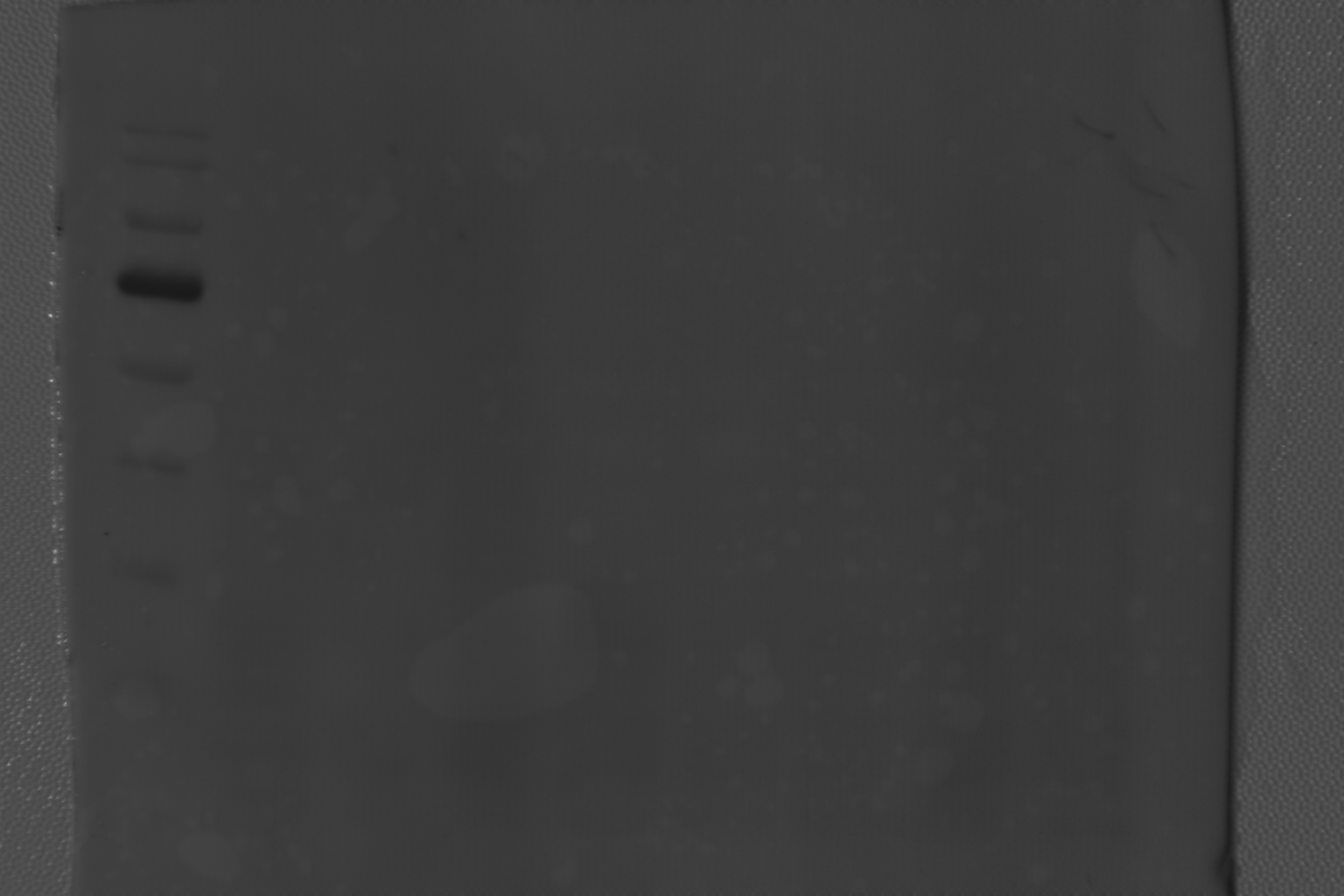

Supplement: Figure 5—source data 2. [file elife-93621-fig5-data2.zip › Figure 5A-Aim17_Protein ladder.tif]

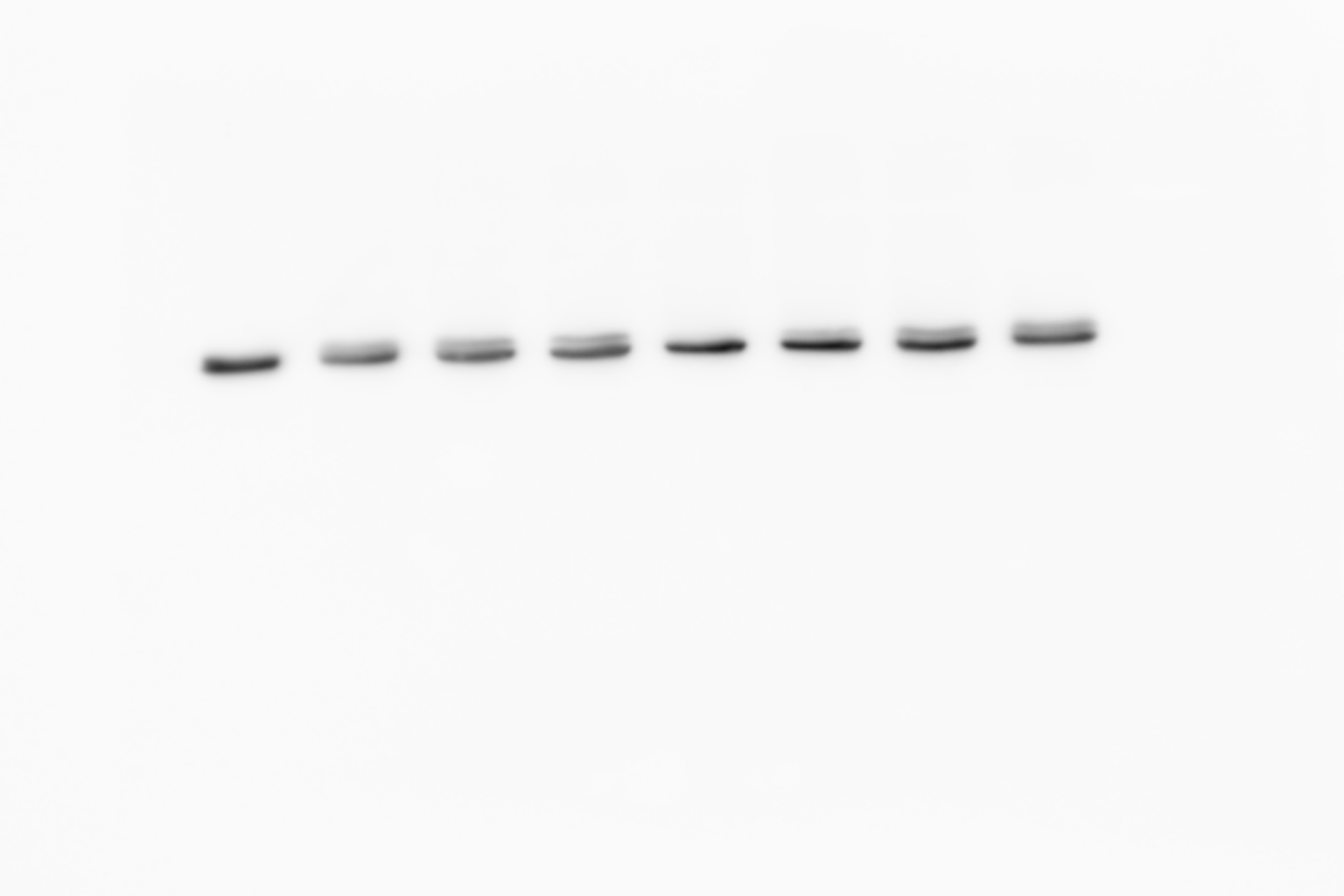

Supplement: Figure 5—source data 2. [file elife-93621-fig5-data2.zip › Figure 5A-Cox5a_Bands.tif]

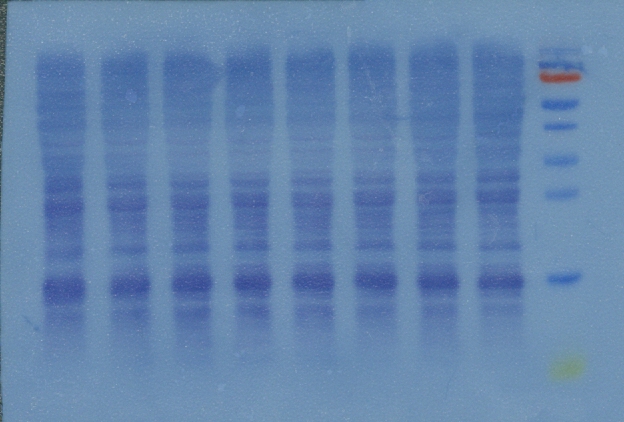

Supplement: Figure 5—source data 2. [file elife-93621-fig5-data2.zip › Figure 5A-Cox5a_DB71.jpg]

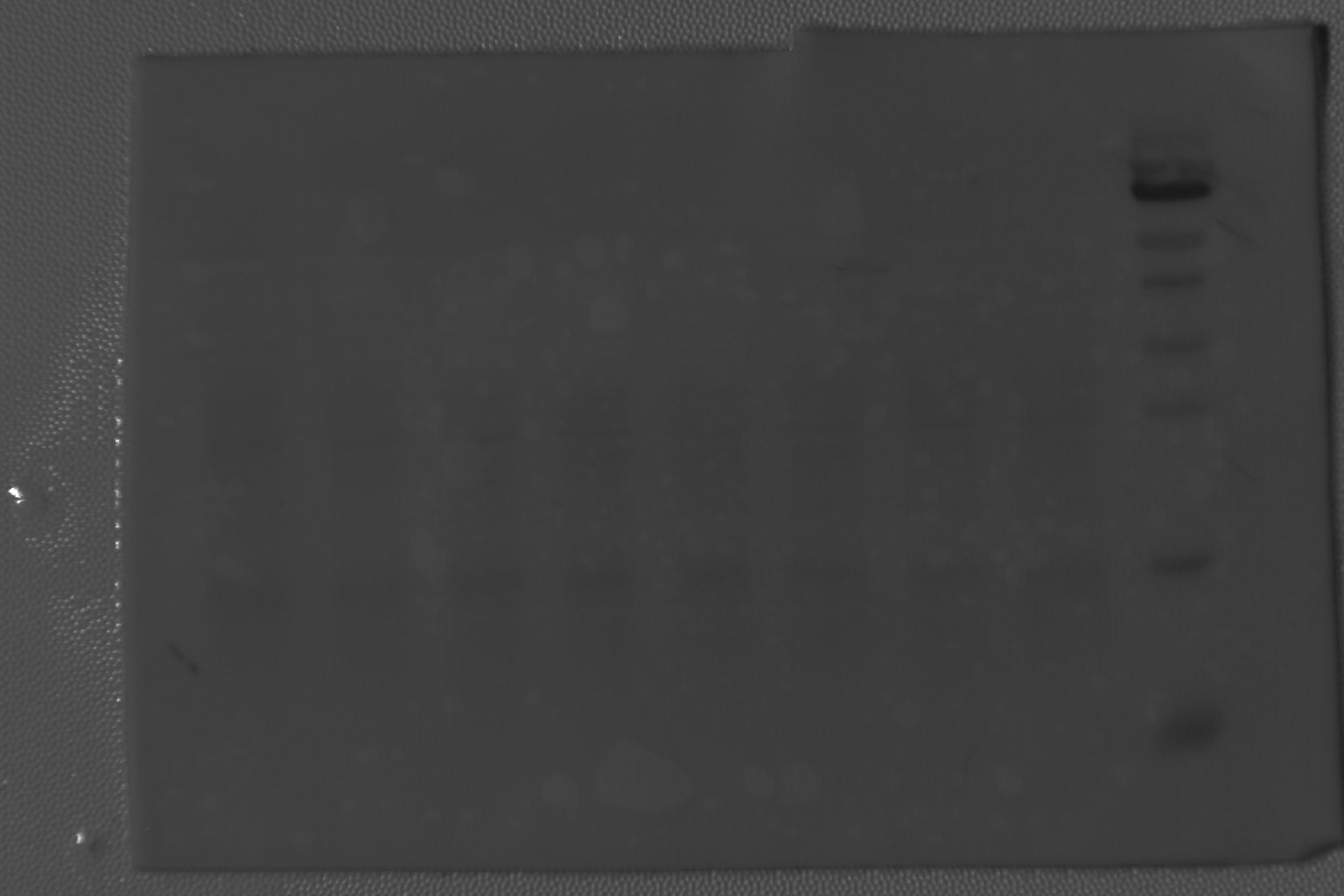

Supplement: Figure 5—source data 2. [file elife-93621-fig5-data2.zip › Figure 5A-Cox5a_Protein ladder.tif]

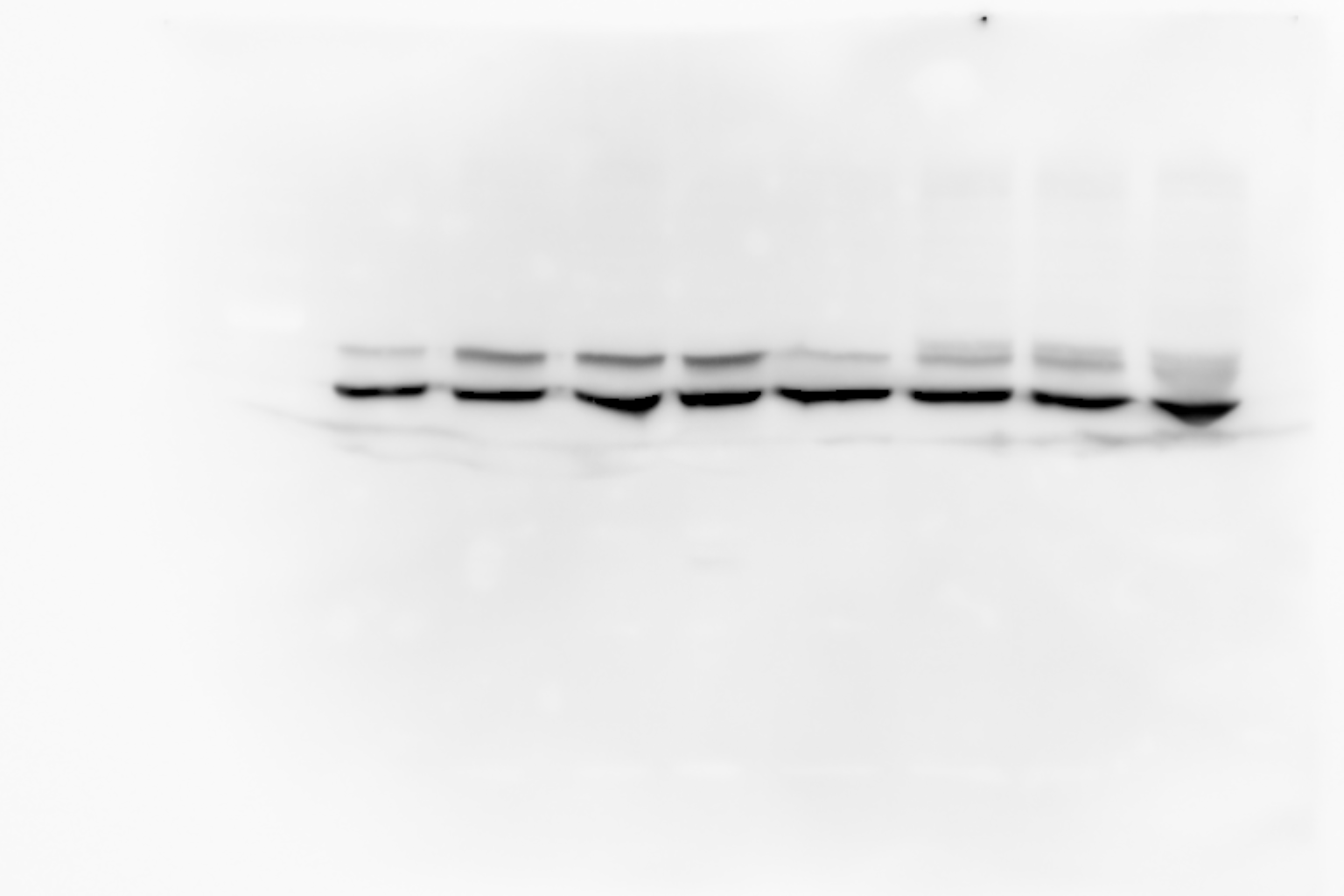

Supplement: Figure 5—source data 2. [file elife-93621-fig5-data2.zip › Figure 5A-Ilv6_Bands.tif]

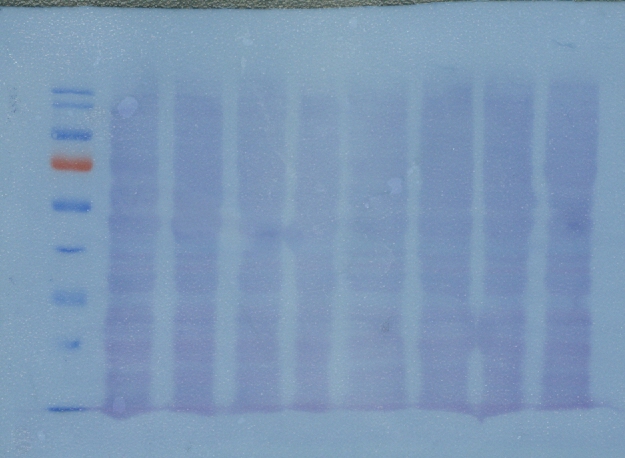

Supplement: Figure 5—source data 2. [file elife-93621-fig5-data2.zip › Figure 5A-Ilv6_DB71.jpg]

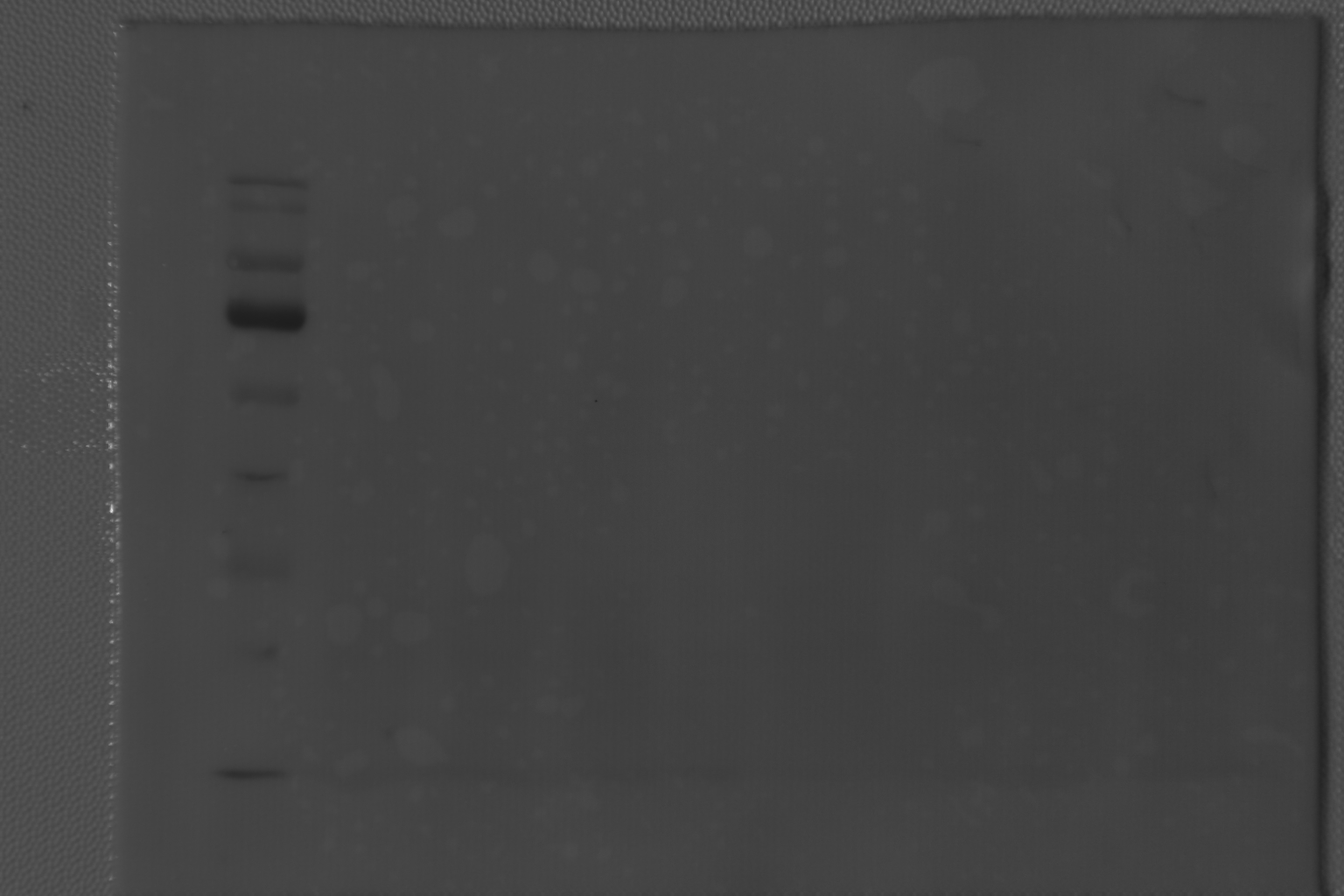

Supplement: Figure 5—source data 2. [file elife-93621-fig5-data2.zip › Figure 5A-Ilv6_Protein ladder.tif]

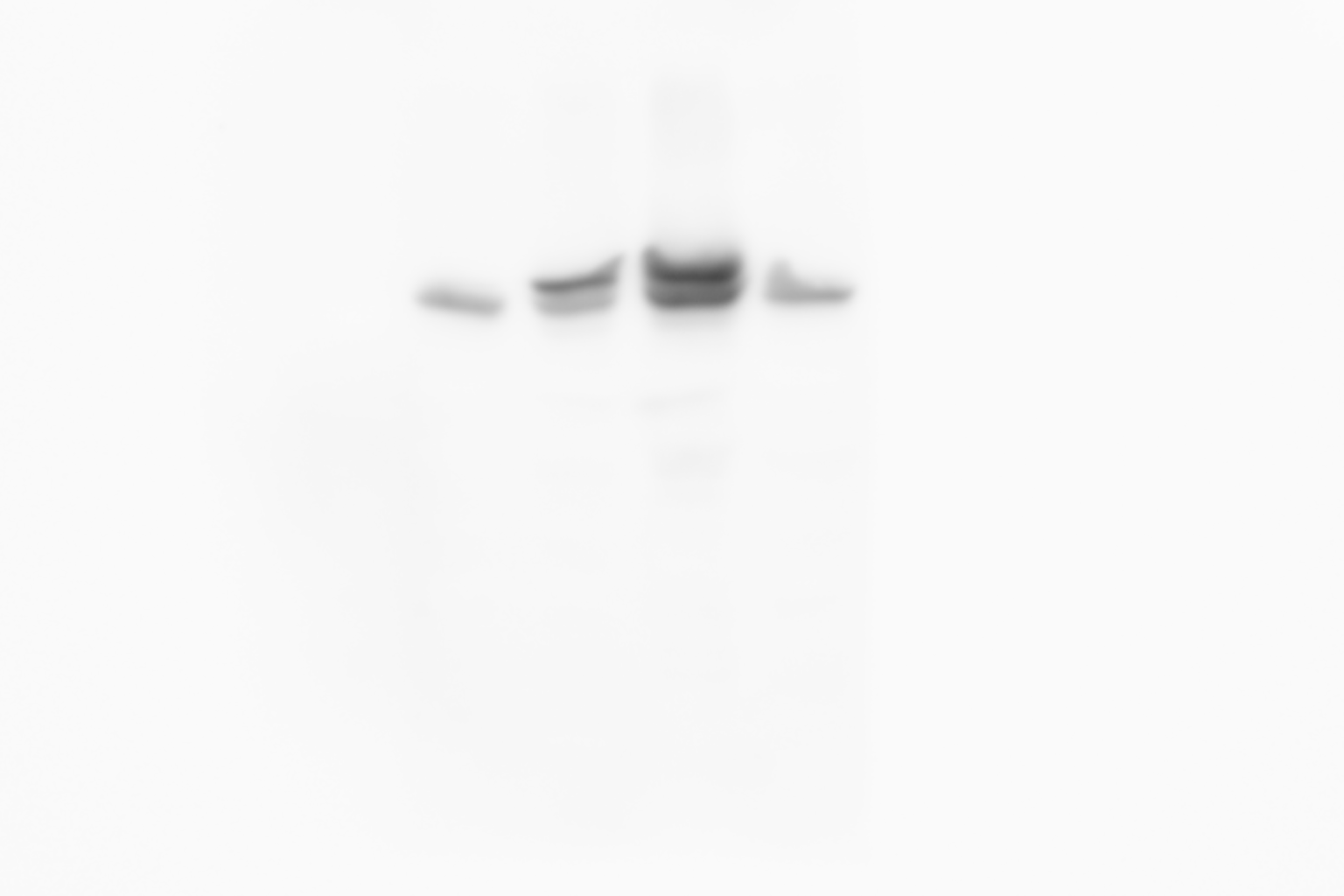

Supplement: Figure 5—source data 2. [file elife-93621-fig5-data2.zip › Figure 5B-Aim17_Bands.tif]

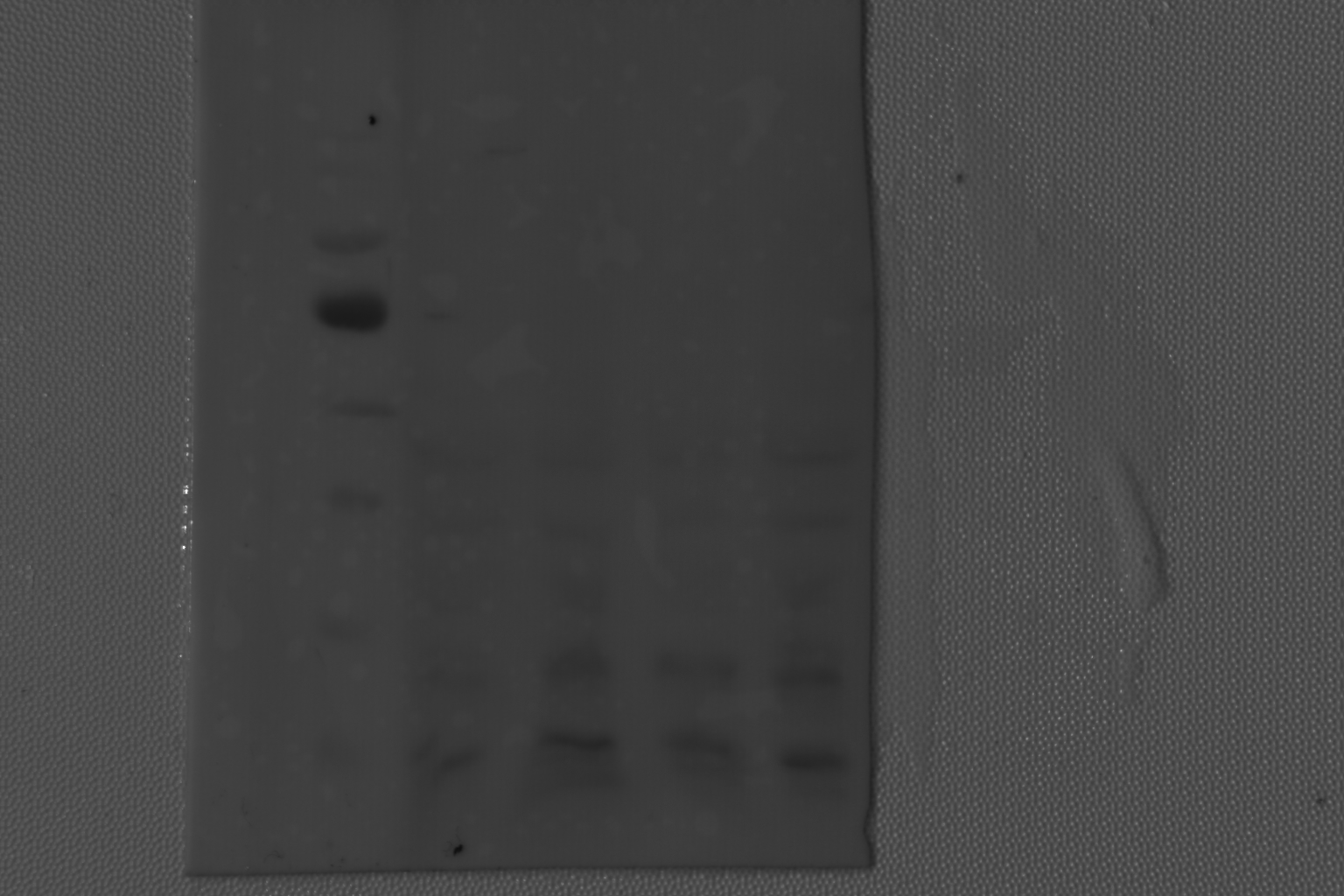

Supplement: Figure 5—source data 2. [file elife-93621-fig5-data2.zip › Figure 5B-Aim17_Protein ladder.tif]

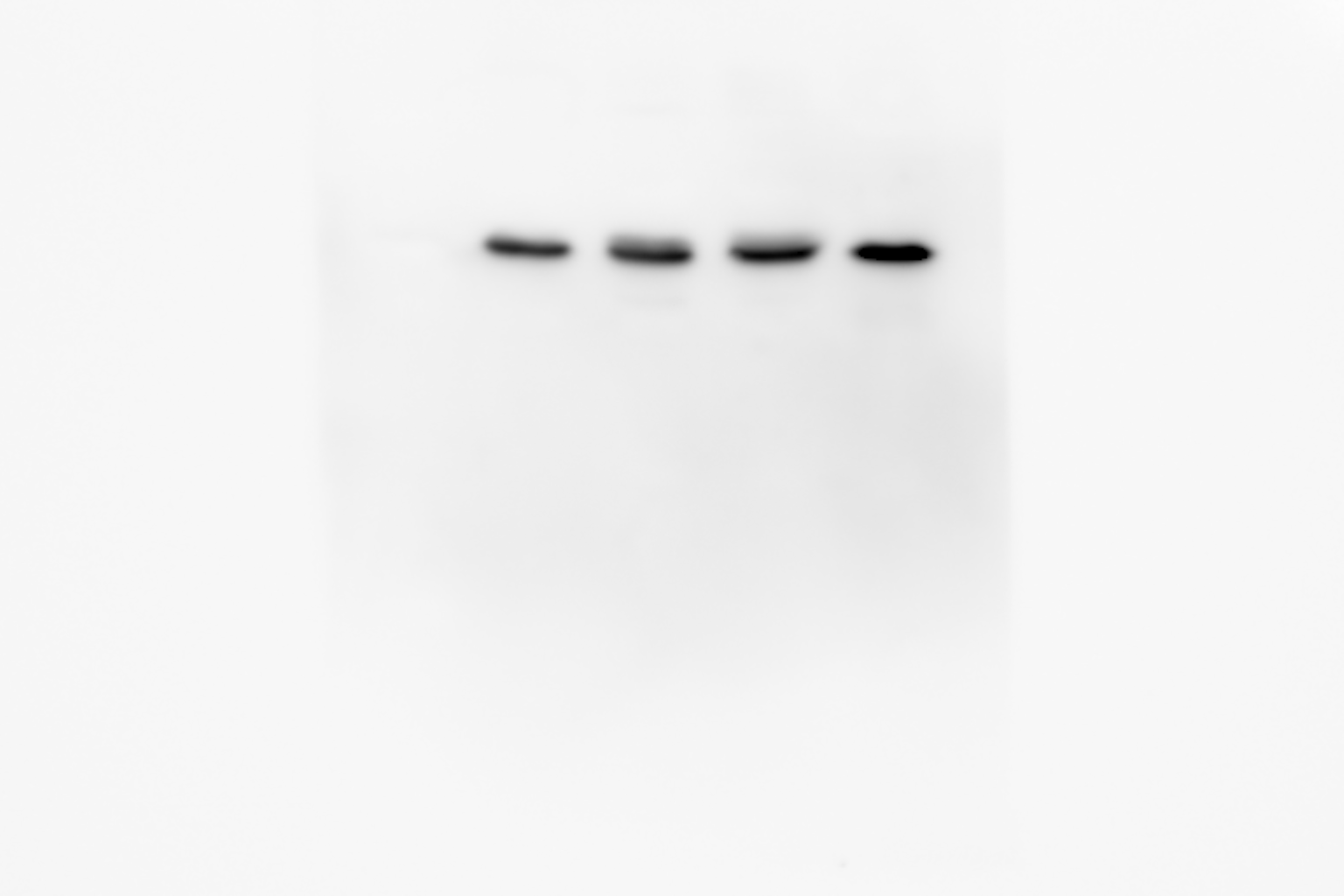

Supplement: Figure 5—source data 2. [file elife-93621-fig5-data2.zip › Figure 5B-Cox5a_Bands.tif]

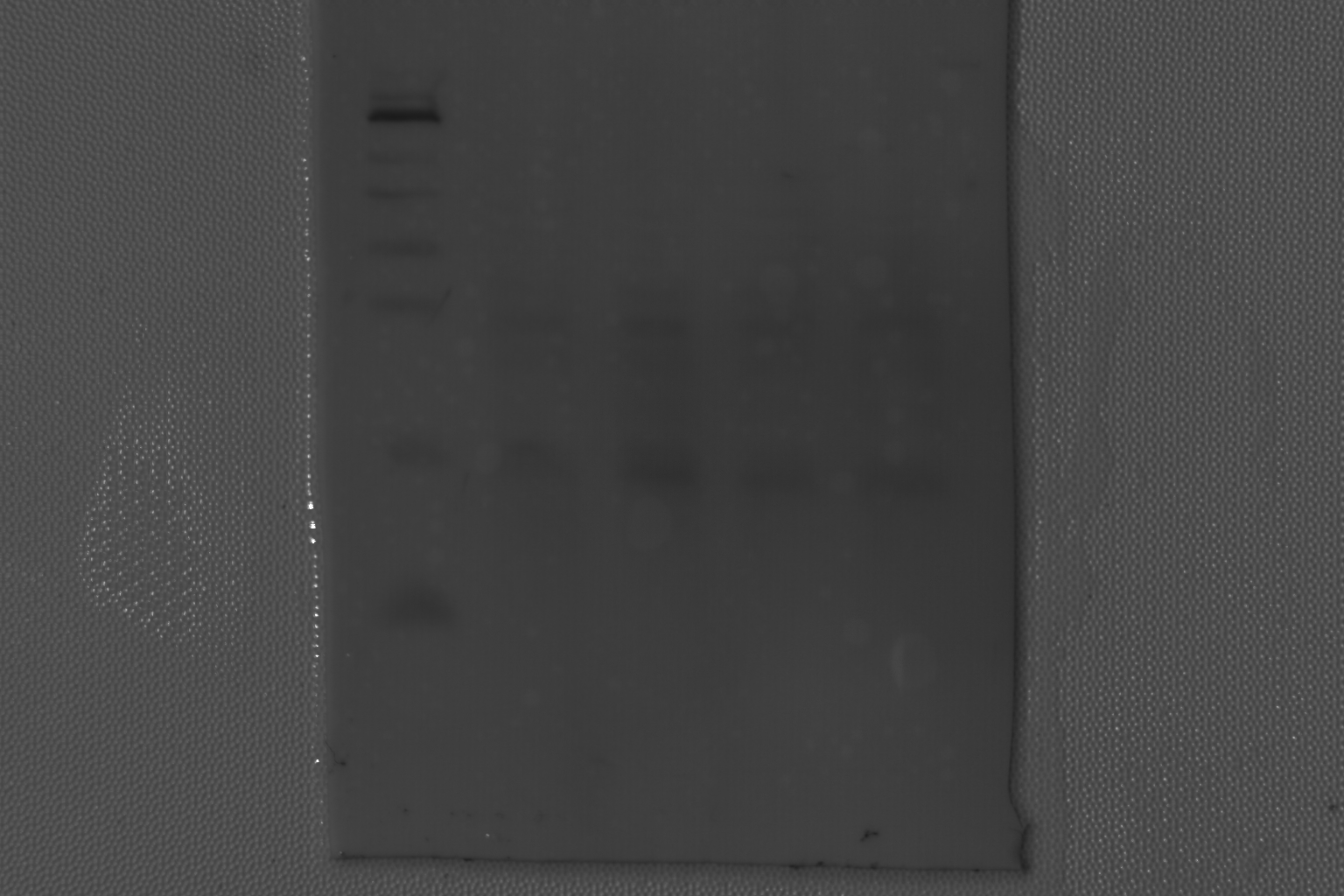

Supplement: Figure 5—source data 2. [file elife-93621-fig5-data2.zip › Figure 5B-Cox5a_Protein ladder.tif]

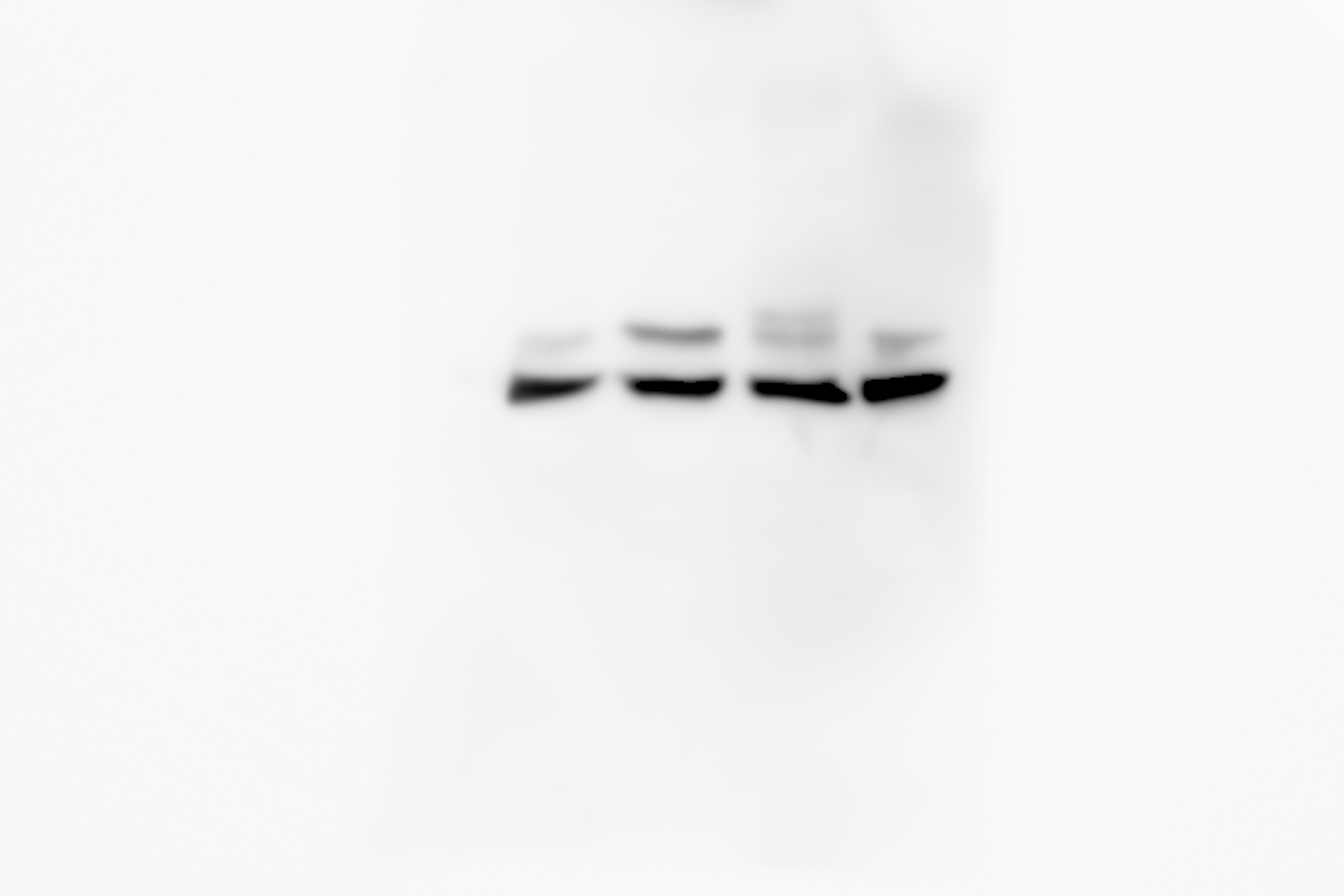

Supplement: Figure 5—source data 2. [file elife-93621-fig5-data2.zip › Figure 5B-Ilv6_Bands.tif]

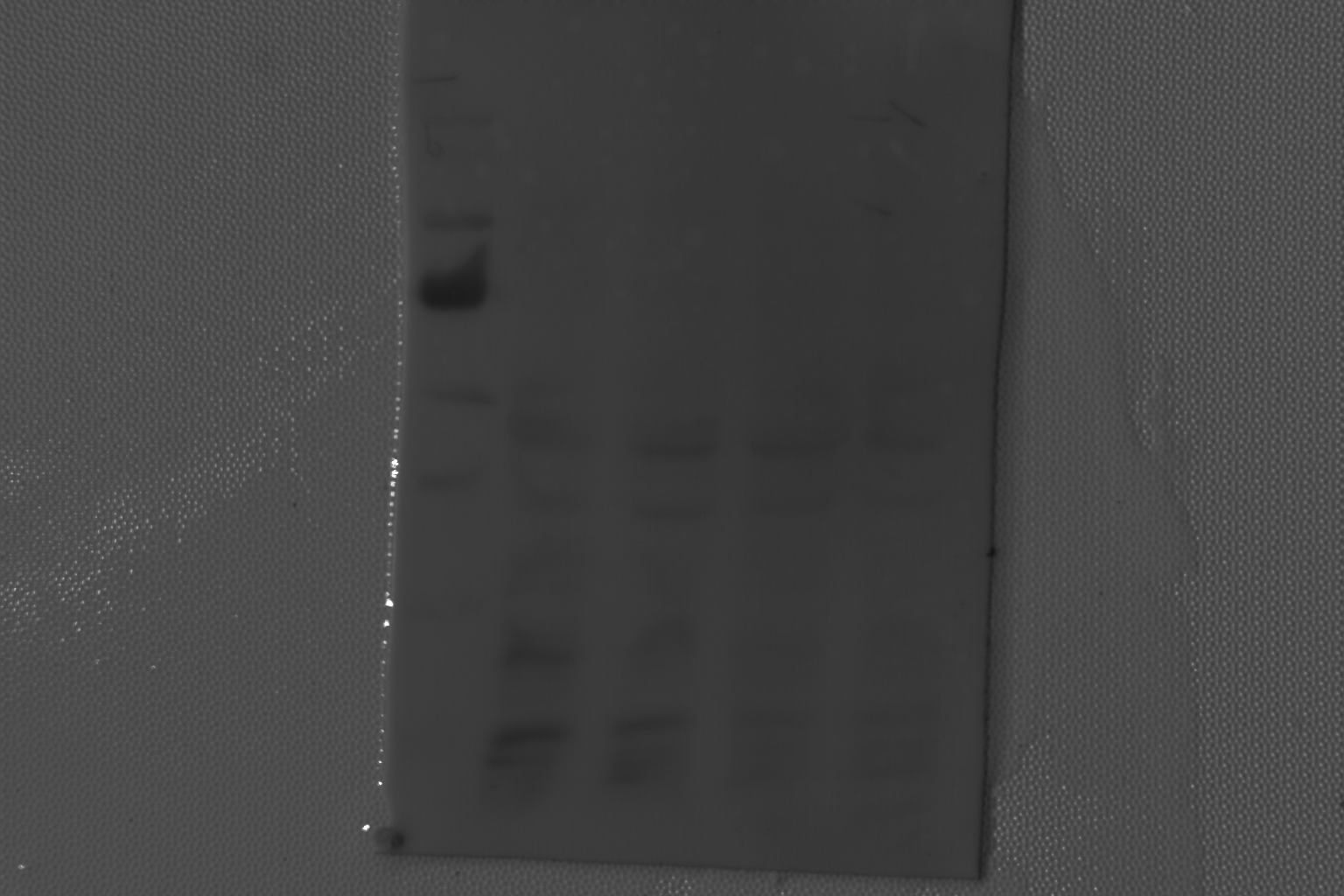

Supplement: Figure 5—source data 2. [file elife-93621-fig5-data2.zip › Figure 5B-Ilv6_Protein ladder.tif]

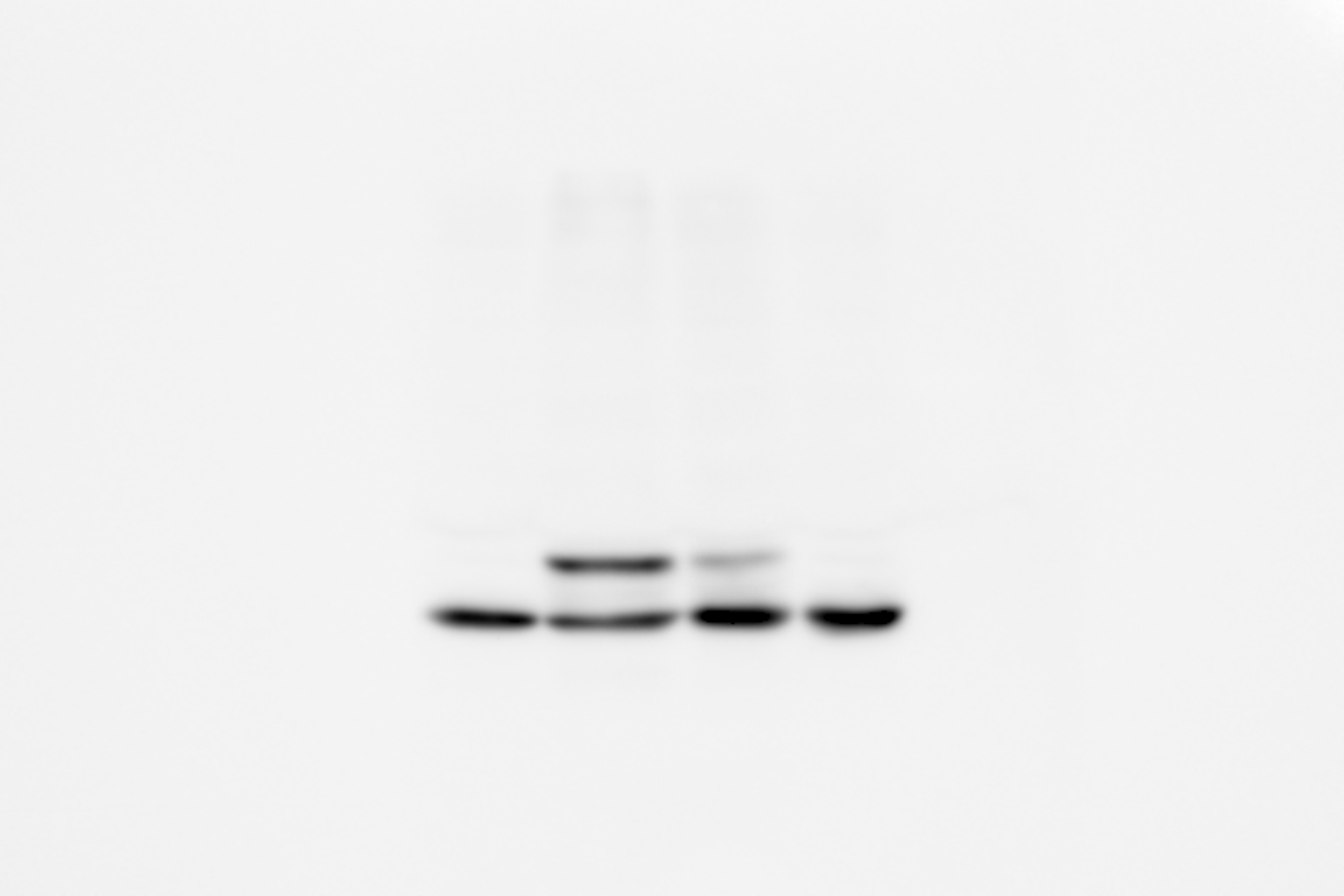

Supplement: Figure 5—source data 2. [file elife-93621-fig5-data2.zip › Figure 5B-Sdh4_Bands.tif]

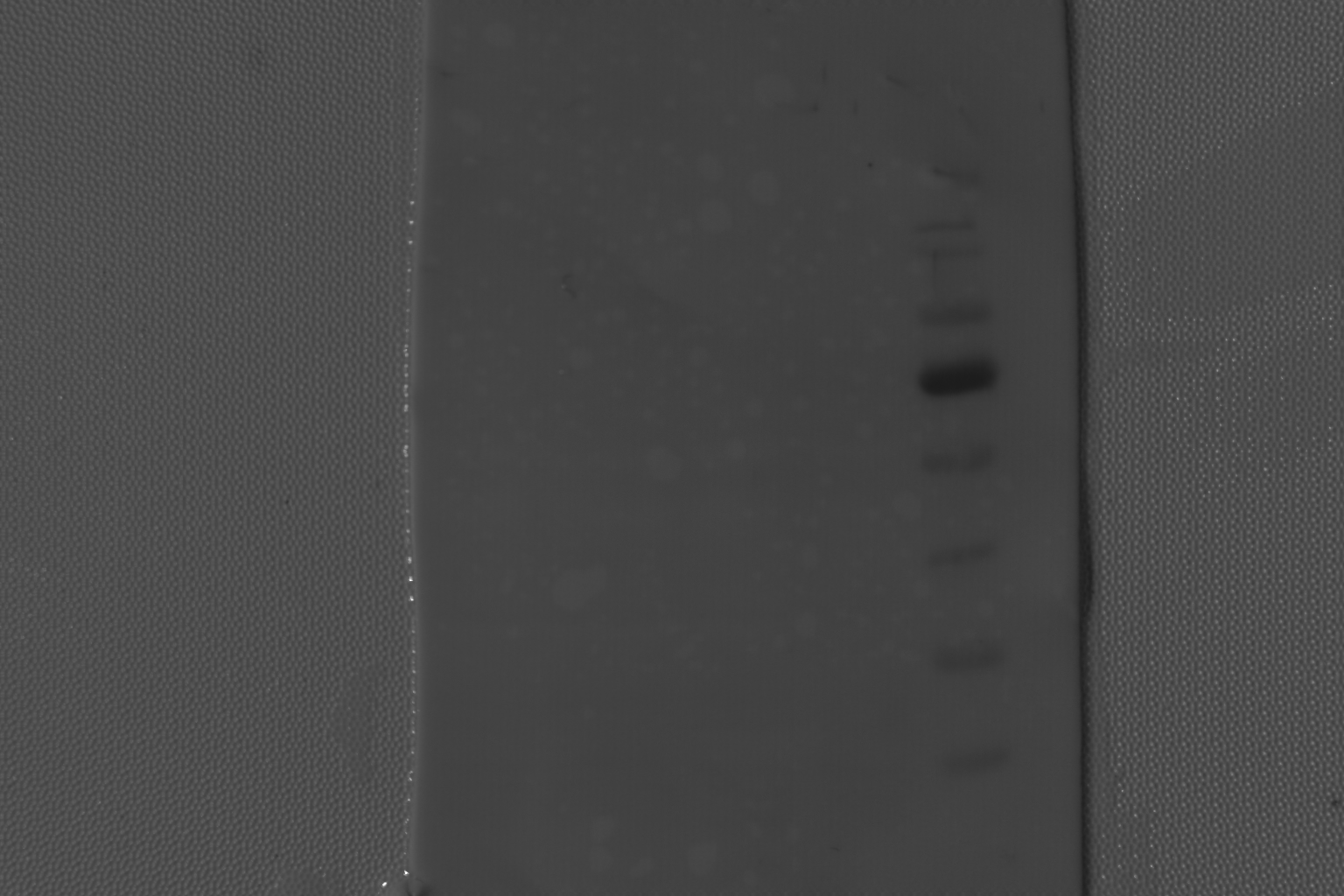

Supplement: Figure 5—source data 2. [file elife-93621-fig5-data2.zip › Figure 5B-Sdh4_Protein ladder.tif]

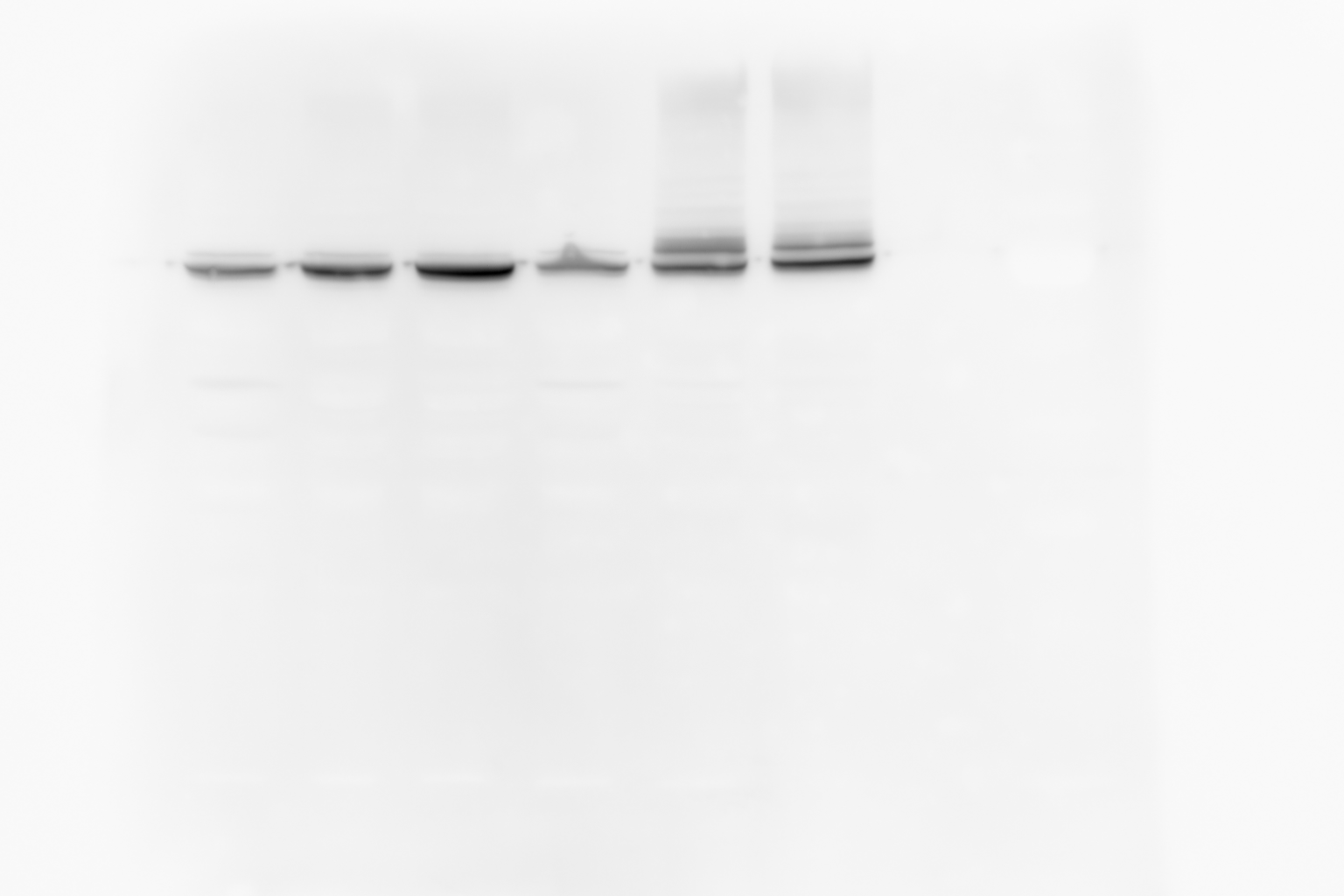

Supplement: Figure 5—source data 2. [file elife-93621-fig5-data2.zip › Figure 5D-Aim17_Bands.tif]

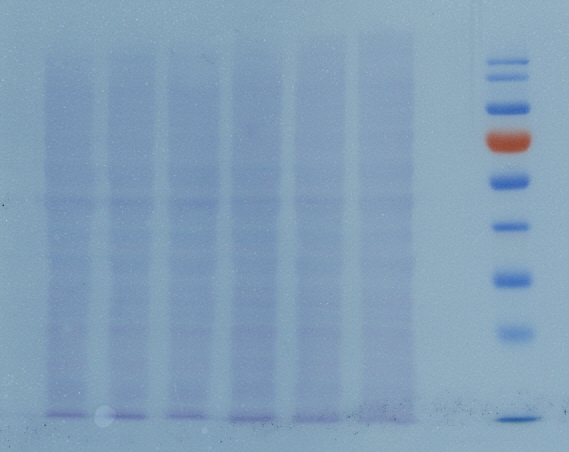

Supplement: Figure 5—source data 2. [file elife-93621-fig5-data2.zip › Figure 5D-Aim17_DB71.jpg]

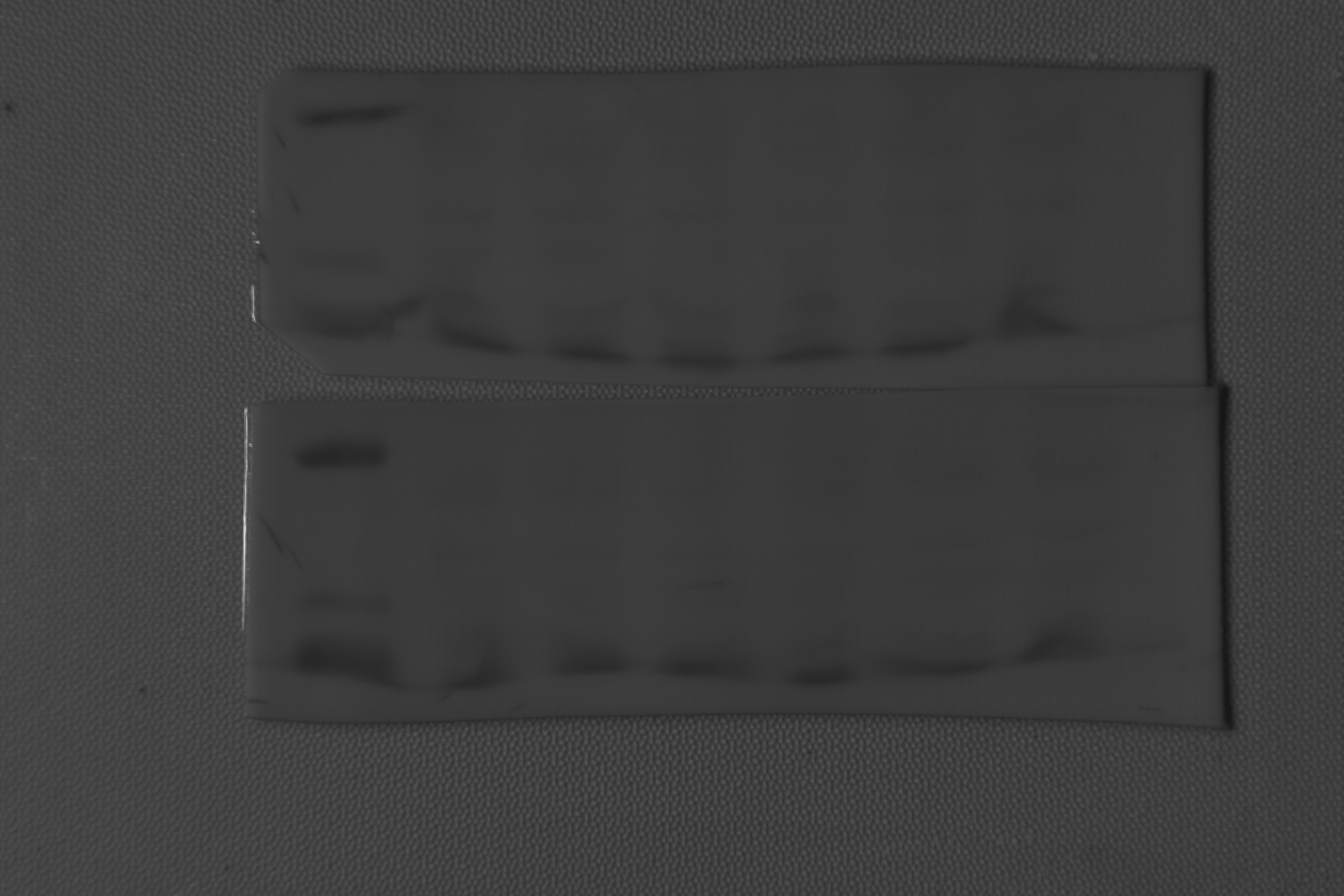

Supplement: Figure 5—source data 3. [file elife-93621-fig5-data3.zip › Figure 5G-WT_Pgk1_Protein ladder.tif]

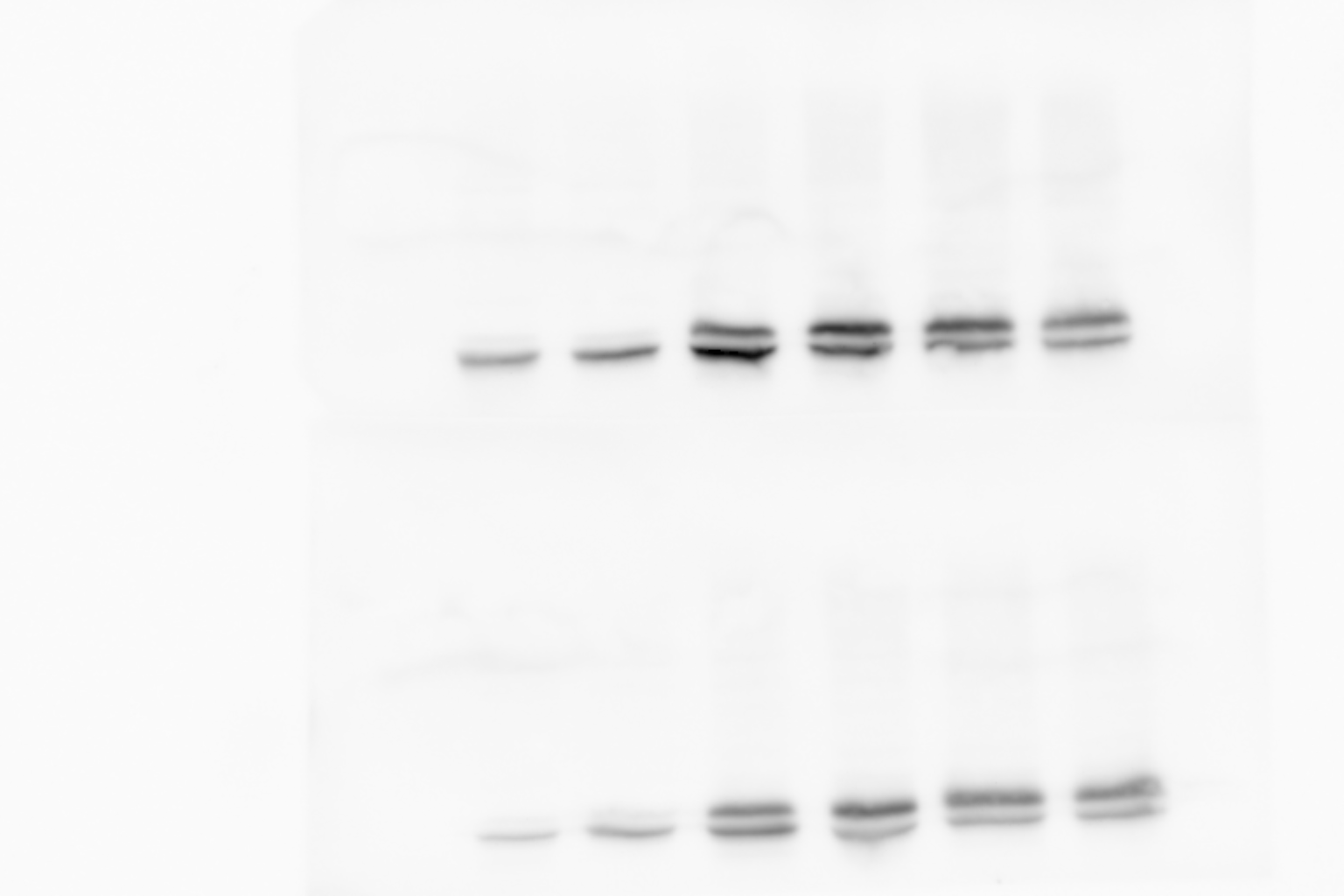

Supplement: Figure 5—source data 3. [file elife-93621-fig5-data3.zip › Figure 5G-WT_TAP_Bands.tif]

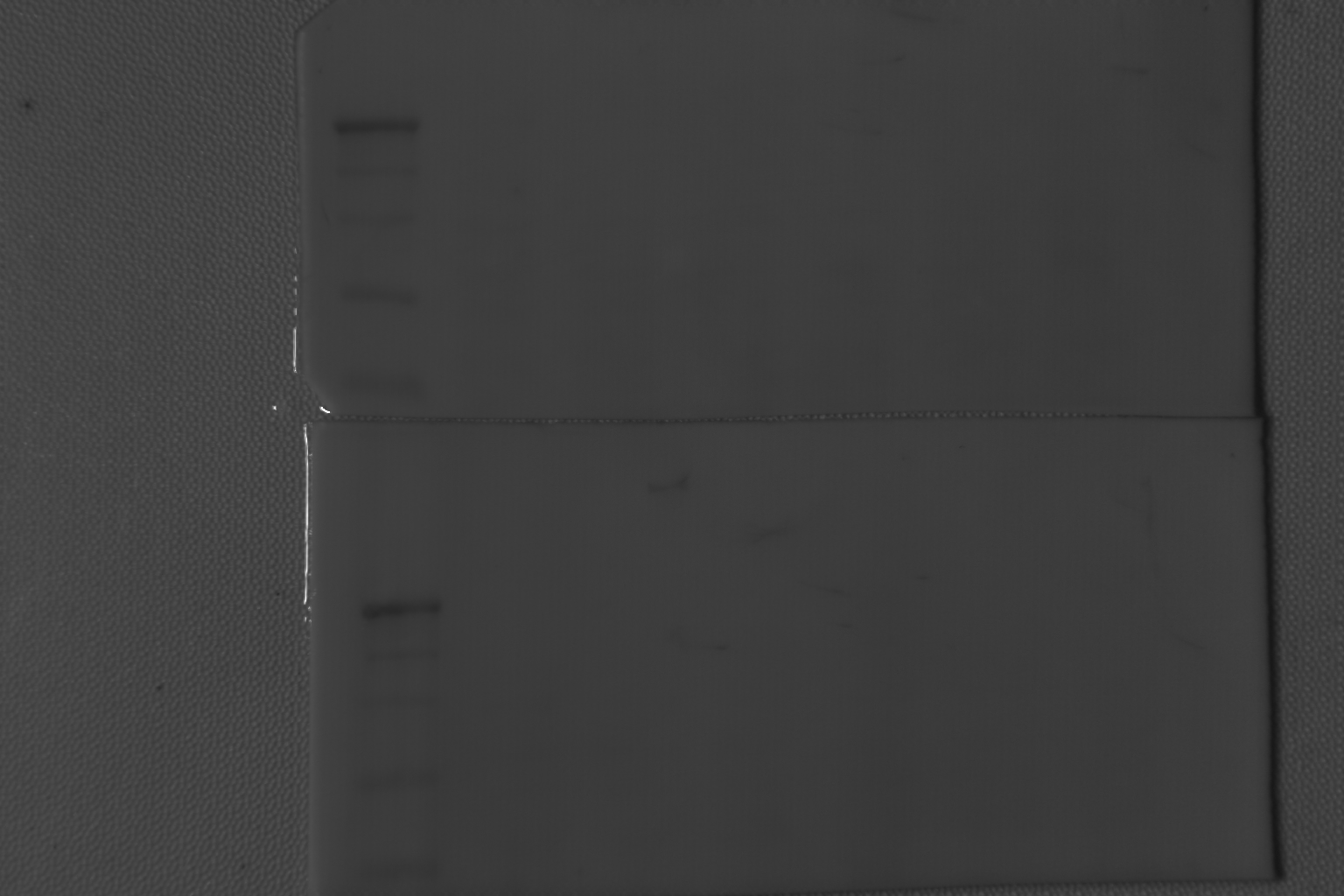

Supplement: Figure 5—source data 3. [file elife-93621-fig5-data3.zip › Figure 5G-WT_TAP_Protein ladder.tif]

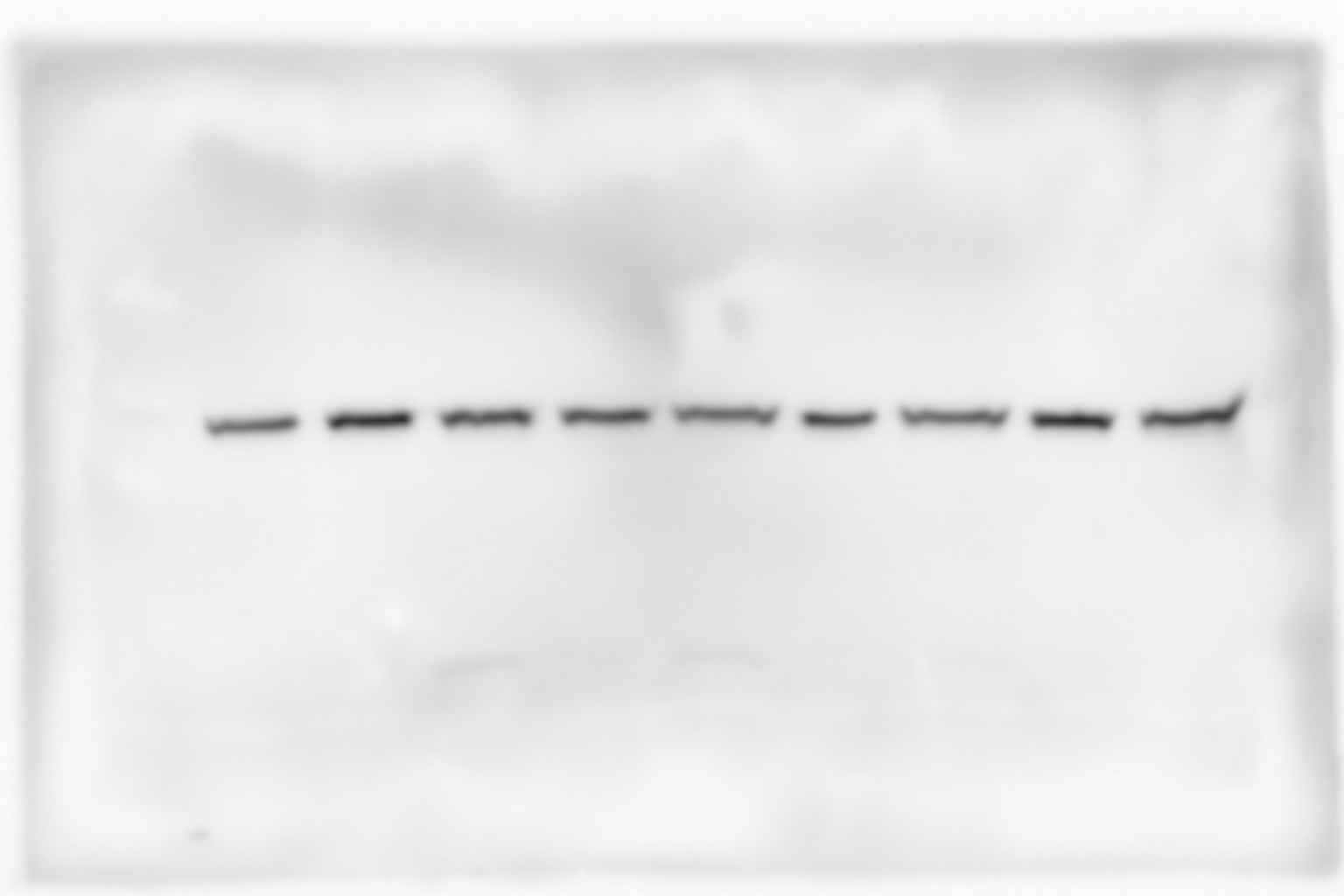

Supplement: Figure 5—source data 3. [file elife-93621-fig5-data3.zip › Figure 5H-Pgk1_Bands.tif]

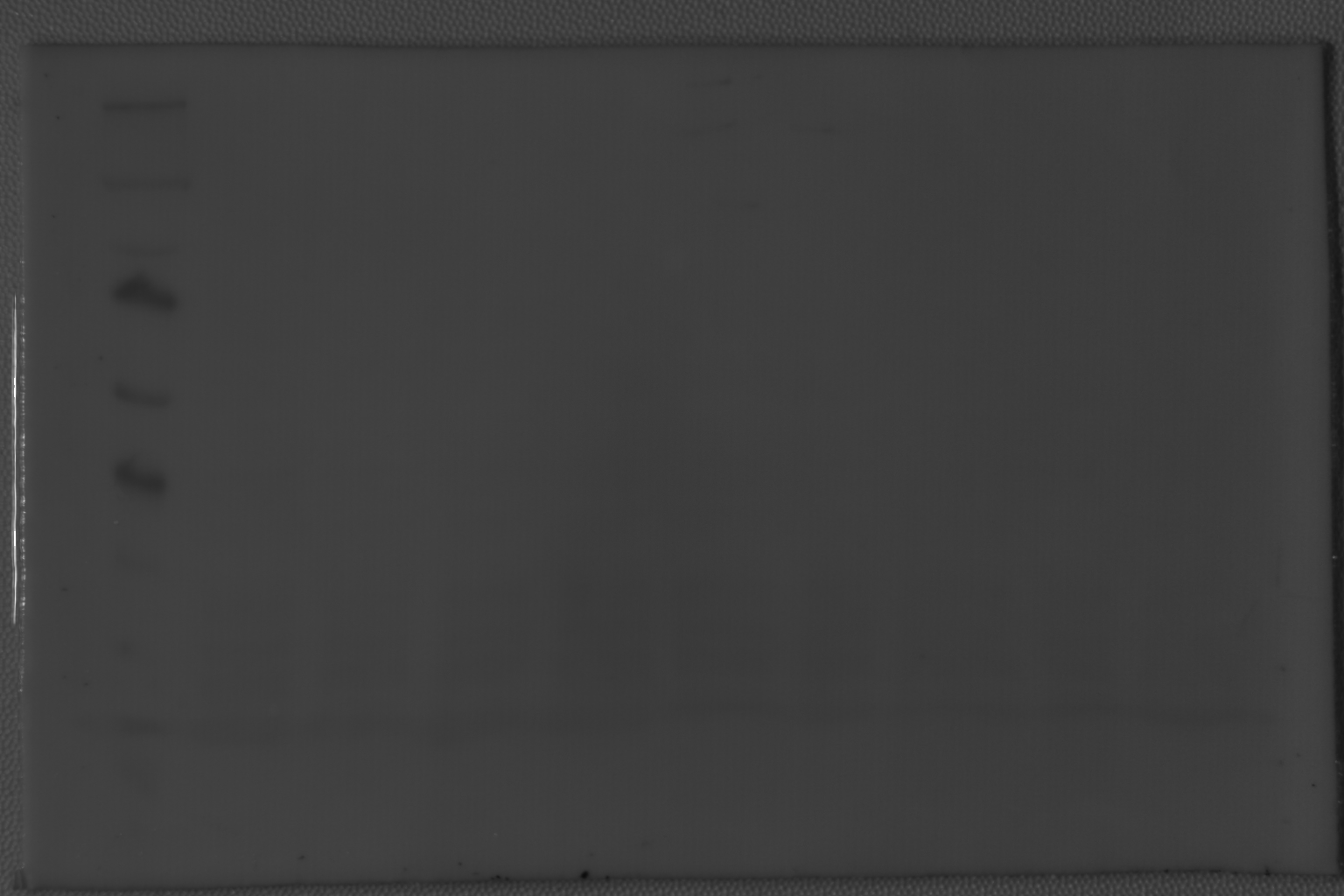

Supplement: Figure 5—source data 3. [file elife-93621-fig5-data3.zip › Figure 5H-Pgk1_Protein ladder.tif]

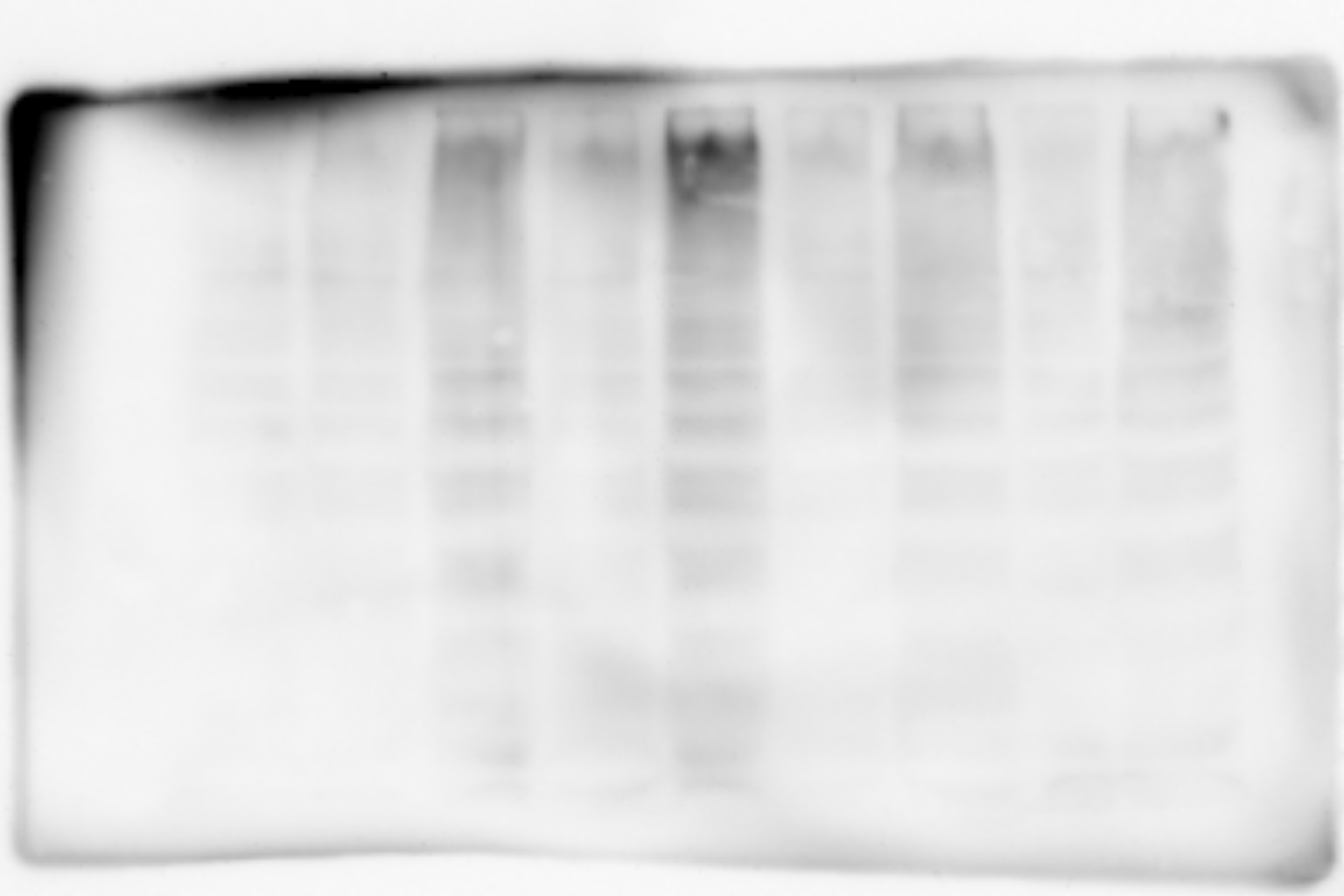

Supplement: Figure 5—source data 3. [file elife-93621-fig5-data3.zip › Figure 5H-TAP_Bands.tif]

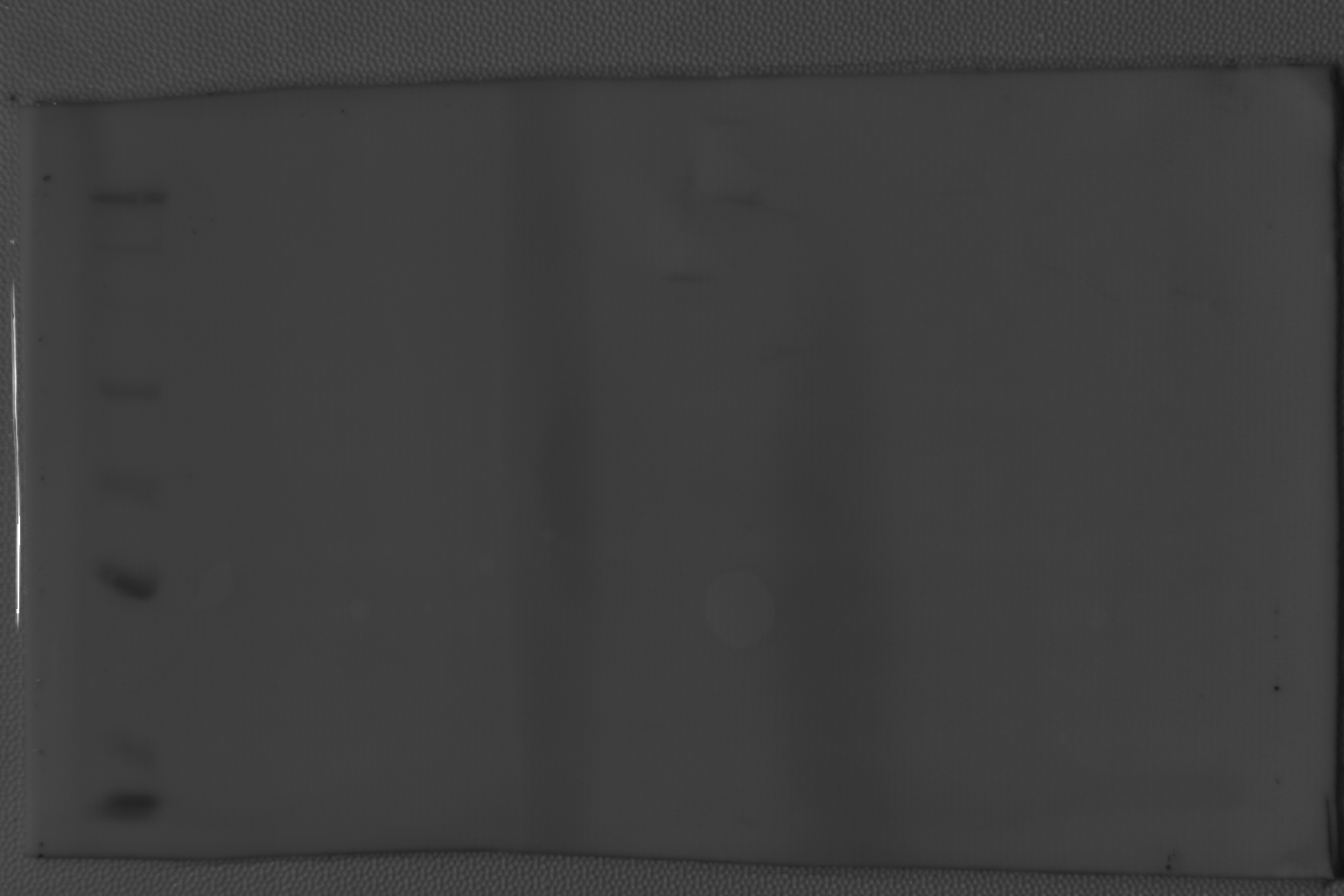

Supplement: Figure 5—source data 3. [file elife-93621-fig5-data3.zip › Figure 5H-TAP_Protein ladder.tif]

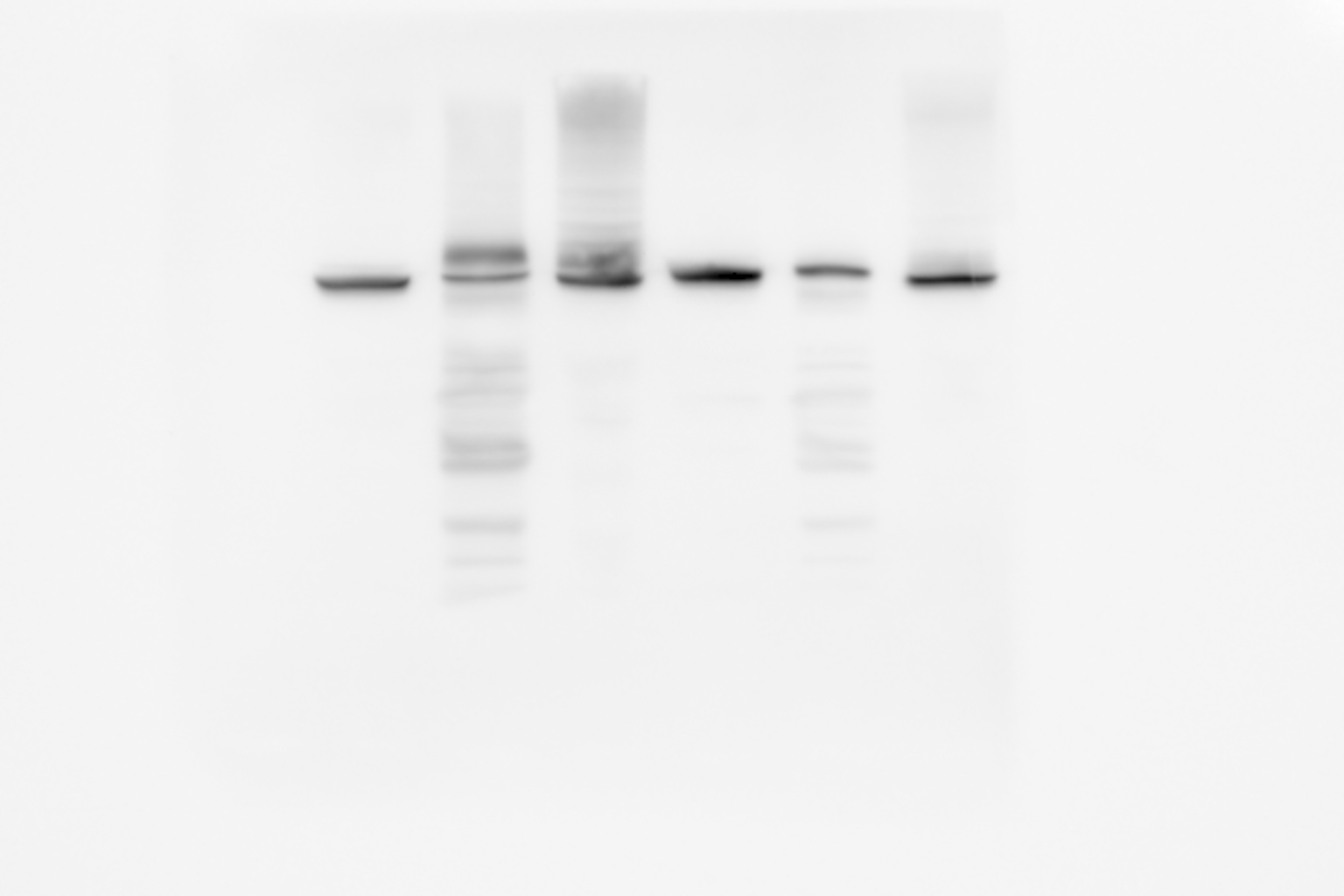

Supplement: Figure 5—source data 3. [file elife-93621-fig5-data3.zip › Figure 5E-Aim17_Bands.tif]

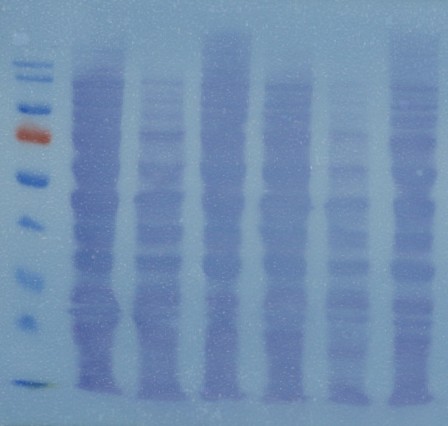

Supplement: Figure 5—source data 3. [file elife-93621-fig5-data3.zip › Figure 5E-Aim17_DB71.jpg]

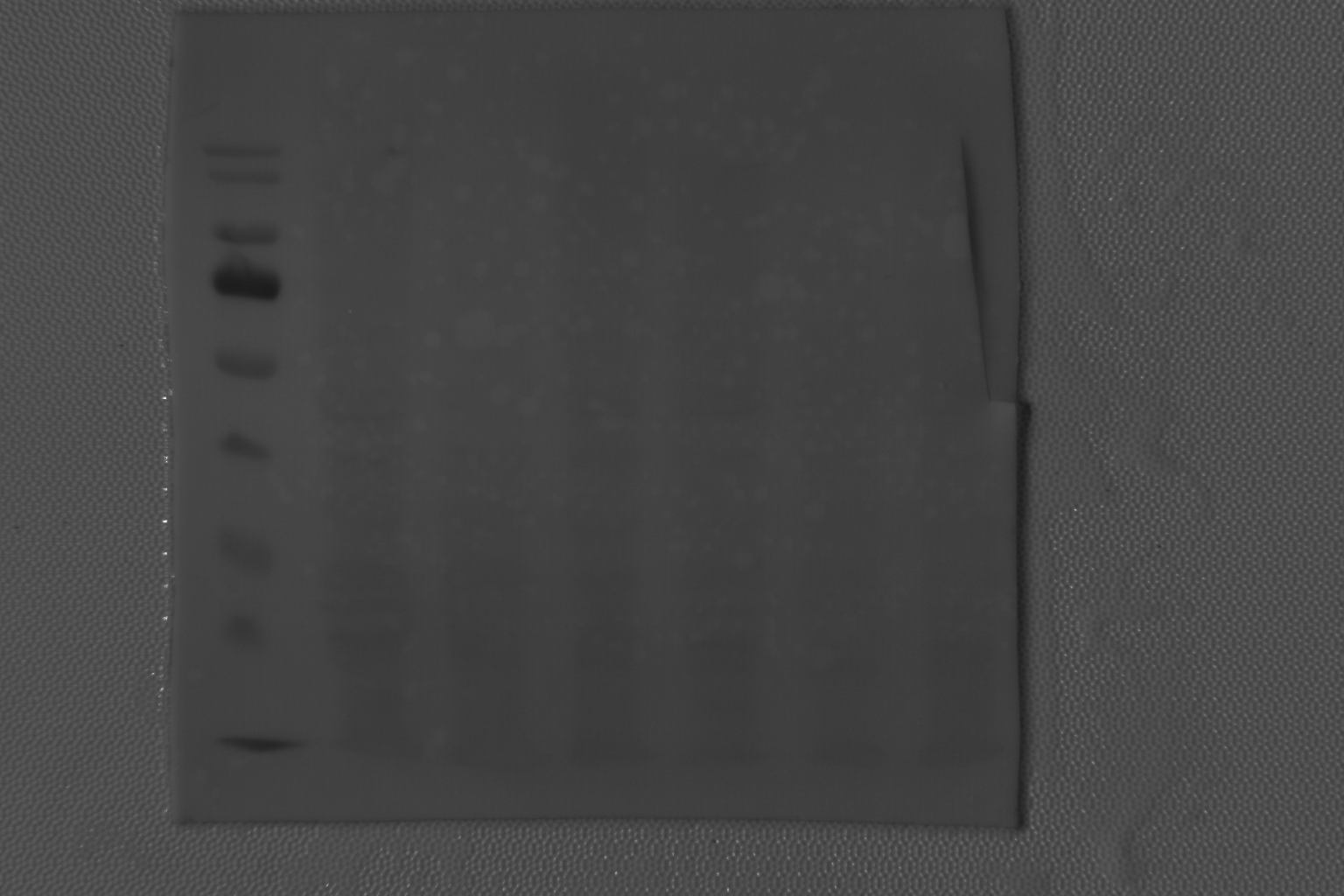

Supplement: Figure 5—source data 3. [file elife-93621-fig5-data3.zip › Figure 5E-Aim17_Protein ladder.tif]

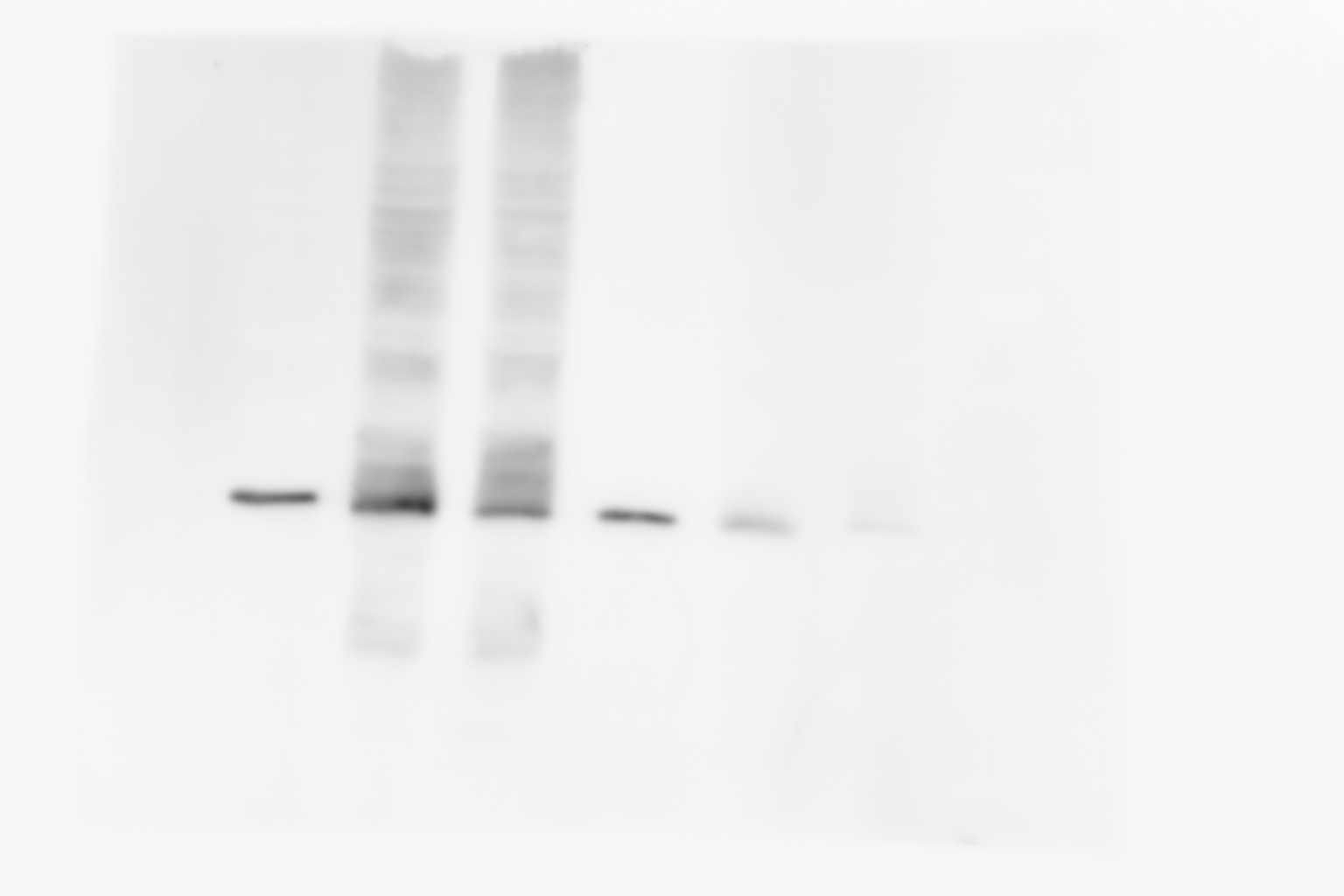

Supplement: Figure 5—source data 3. [file elife-93621-fig5-data3.zip › Figure 5E-Mpc3_Bands.tif]

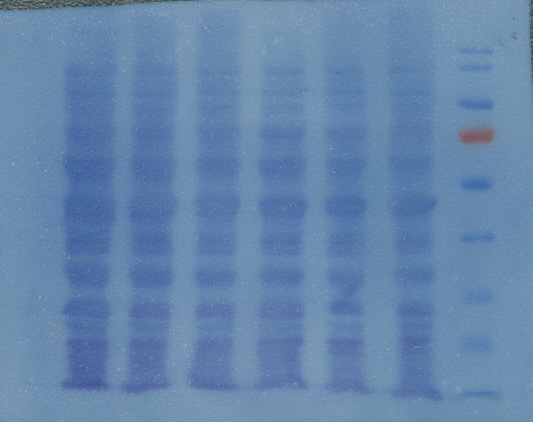

Supplement: Figure 5—source data 3. [file elife-93621-fig5-data3.zip › Figure 5E-Mpc3_DB71.jpg]

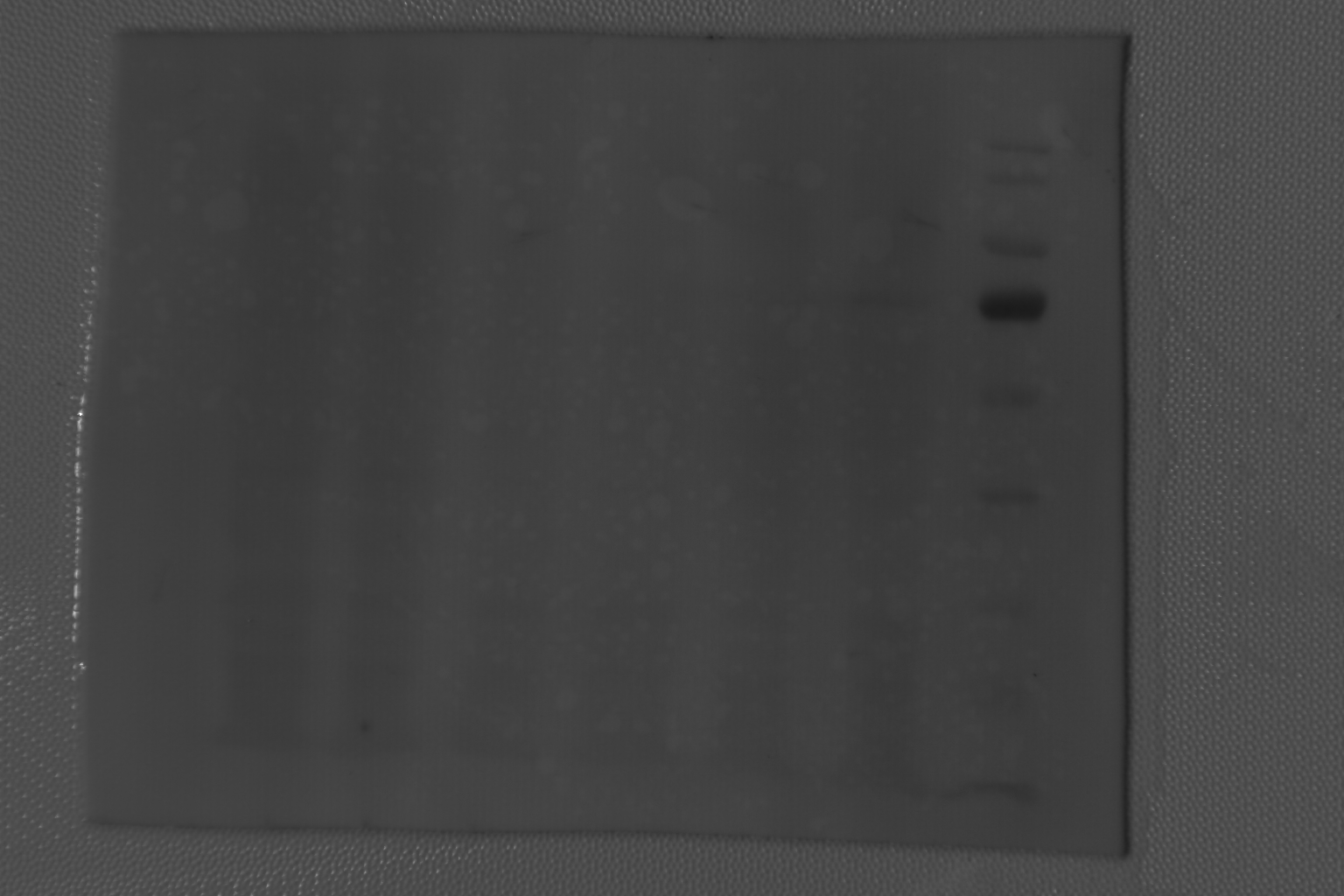

Supplement: Figure 5—source data 3. [file elife-93621-fig5-data3.zip › Figure 5E-Mpc3_Protein ladder.tif]

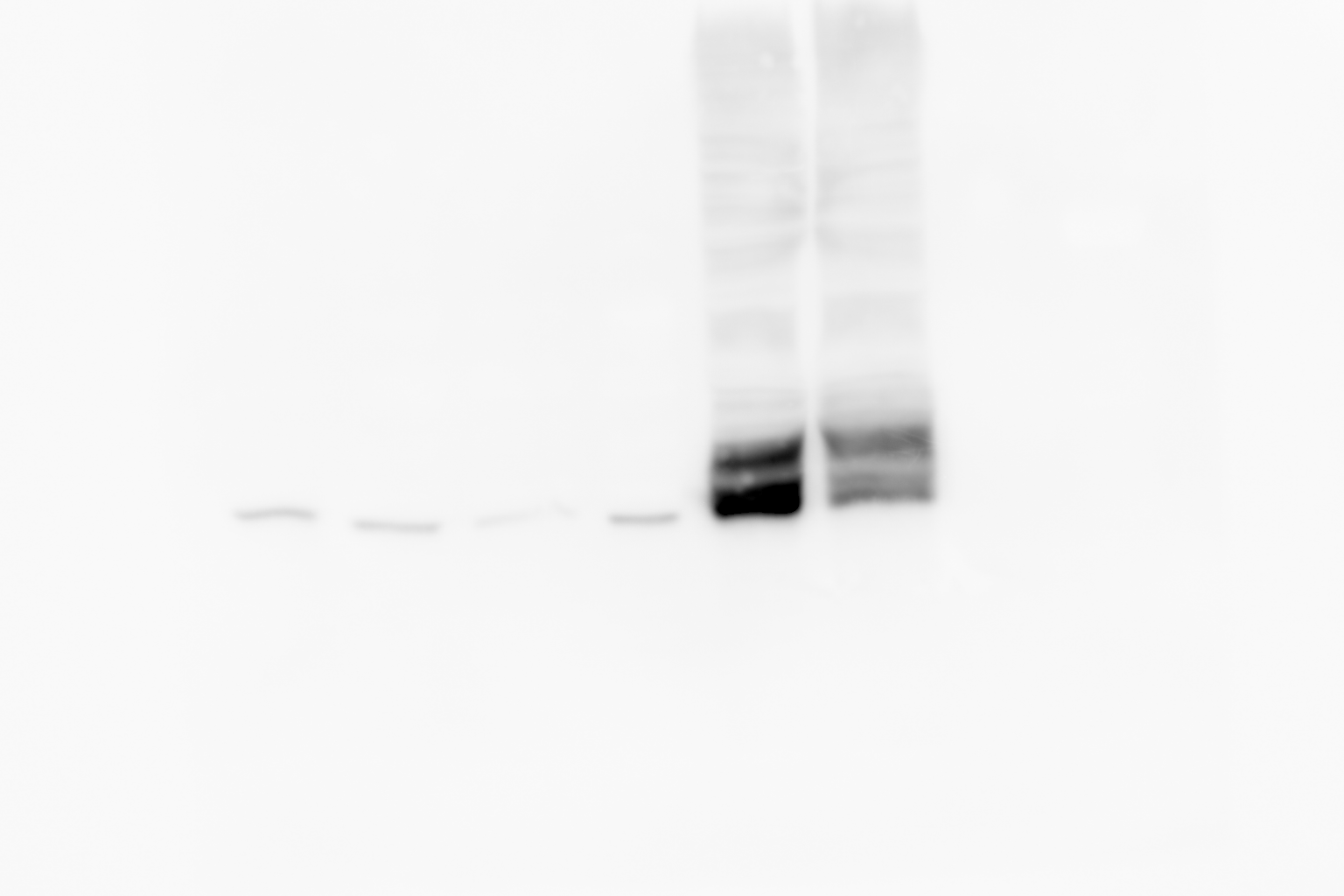

Supplement: Figure 5—source data 3. [file elife-93621-fig5-data3.zip › Figure 5F-Cis1_Bands.tif]

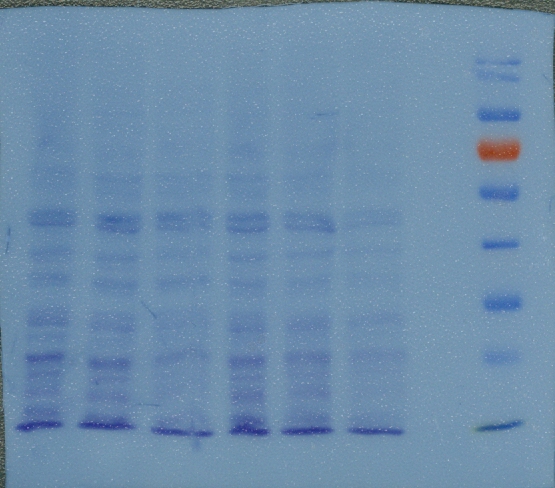

Supplement: Figure 5—source data 3. [file elife-93621-fig5-data3.zip › Figure 5F-Cis1_DB71.jpg]

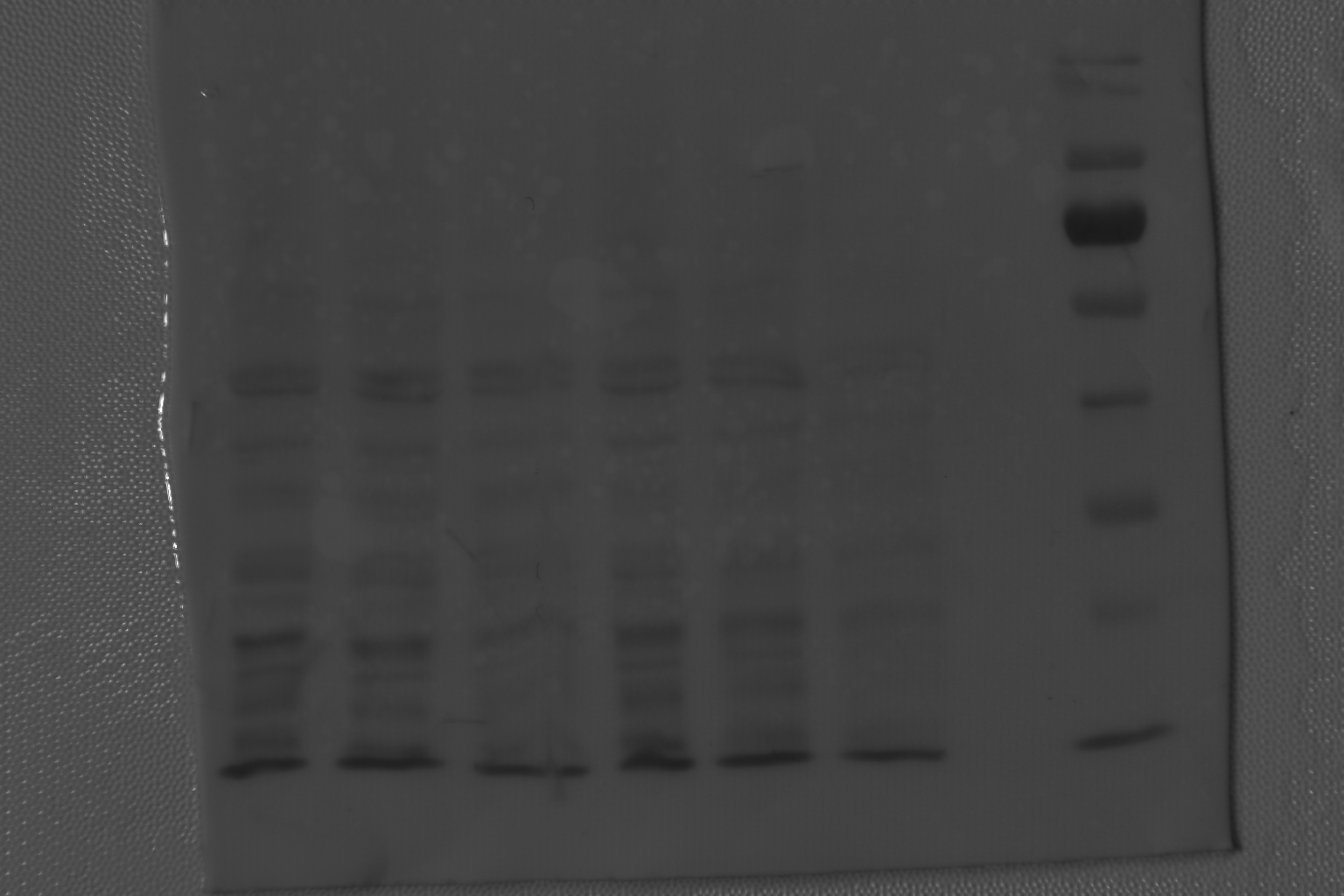

Supplement: Figure 5—source data 3. [file elife-93621-fig5-data3.zip › Figure 5F-Cis1_Protein ladder.tif]

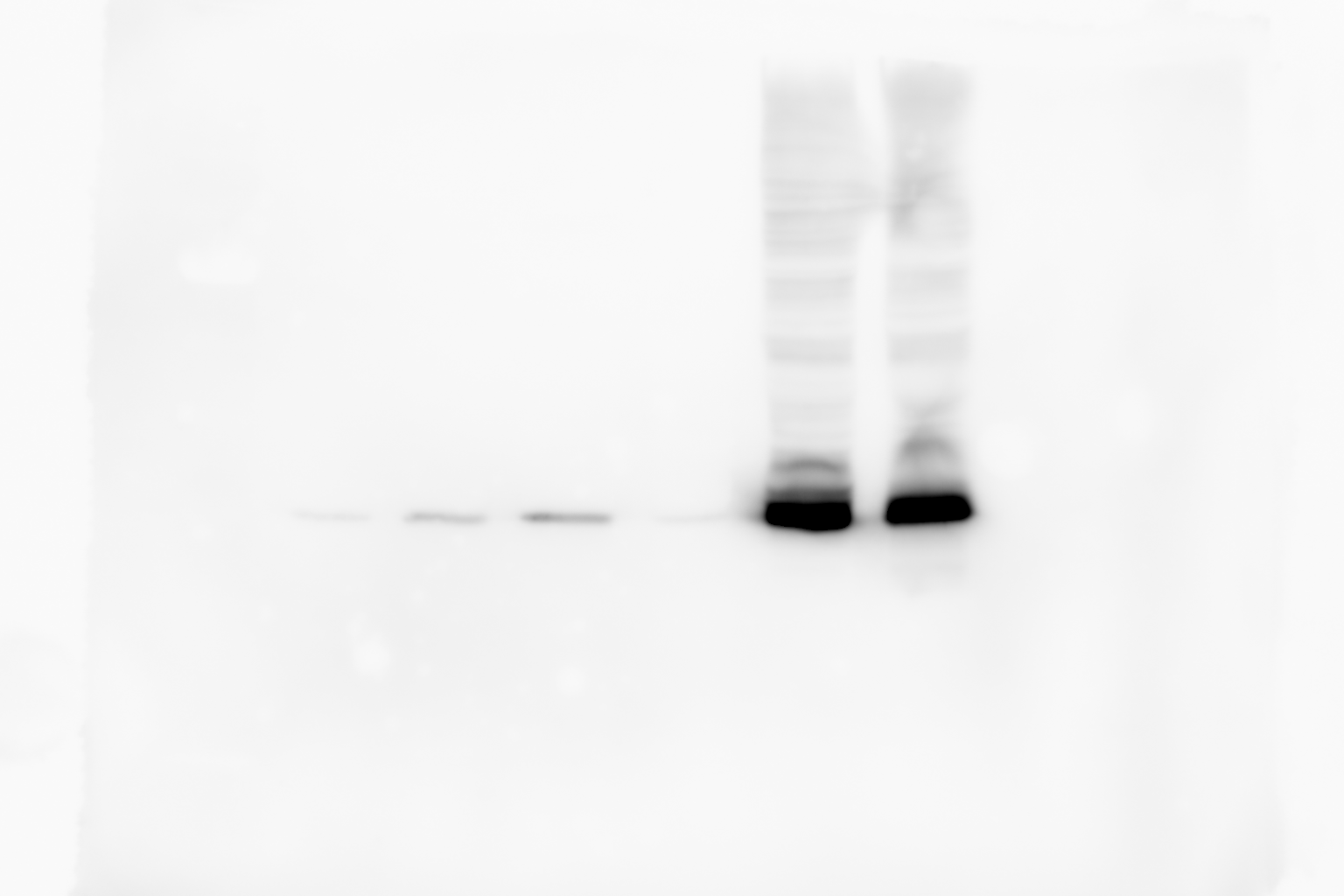

Supplement: Figure 5—source data 3. [file elife-93621-fig5-data3.zip › Figure 5F-Tma10_Bands.tif]

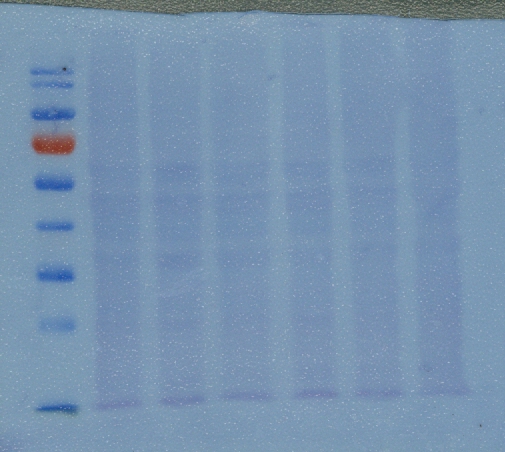

Supplement: Figure 5—source data 3. [file elife-93621-fig5-data3.zip › Figure 5F-Tma10_DB71.jpg]

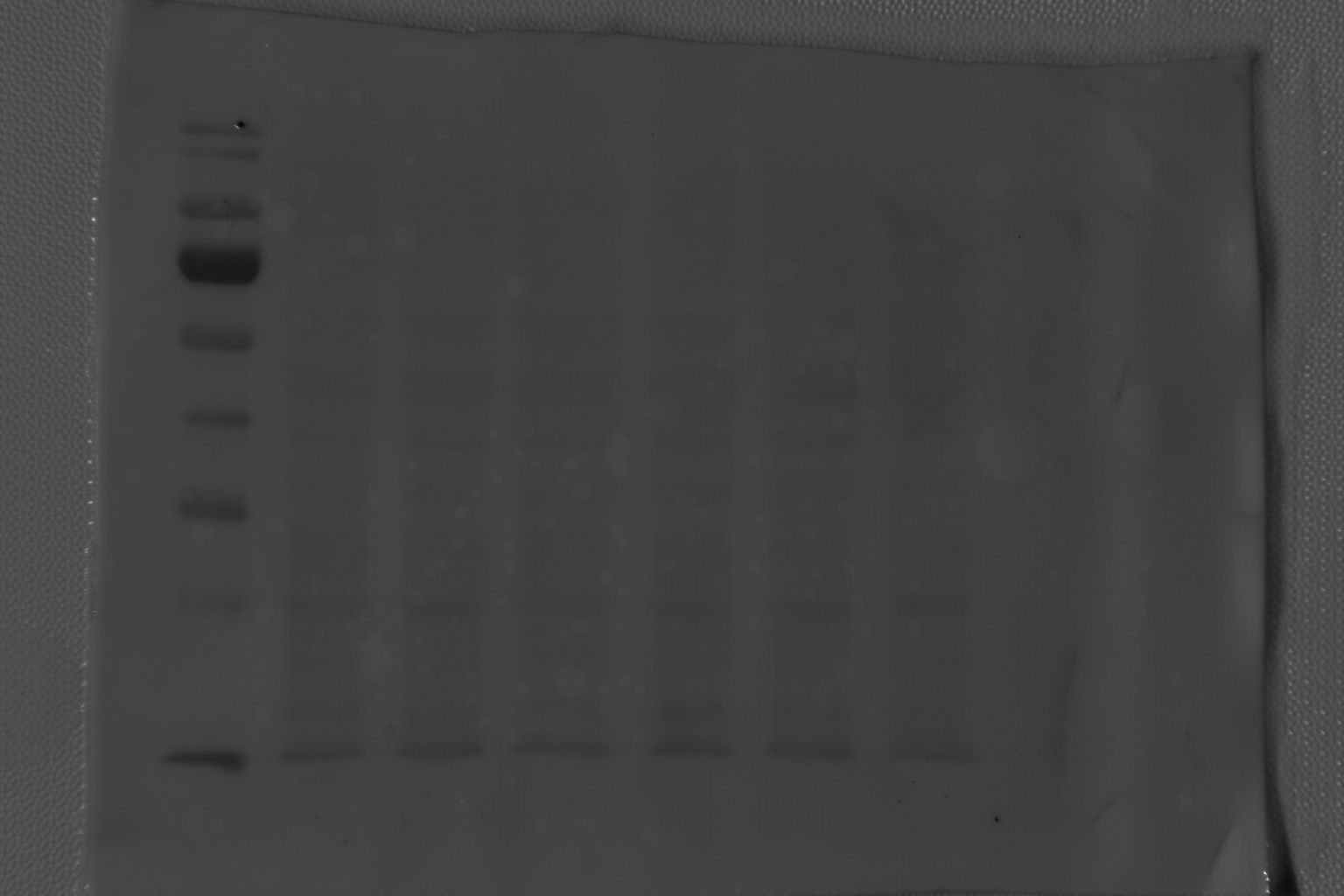

Supplement: Figure 5—source data 3. [file elife-93621-fig5-data3.zip › Figure 5F-Tma10_Protein ladder.tif]

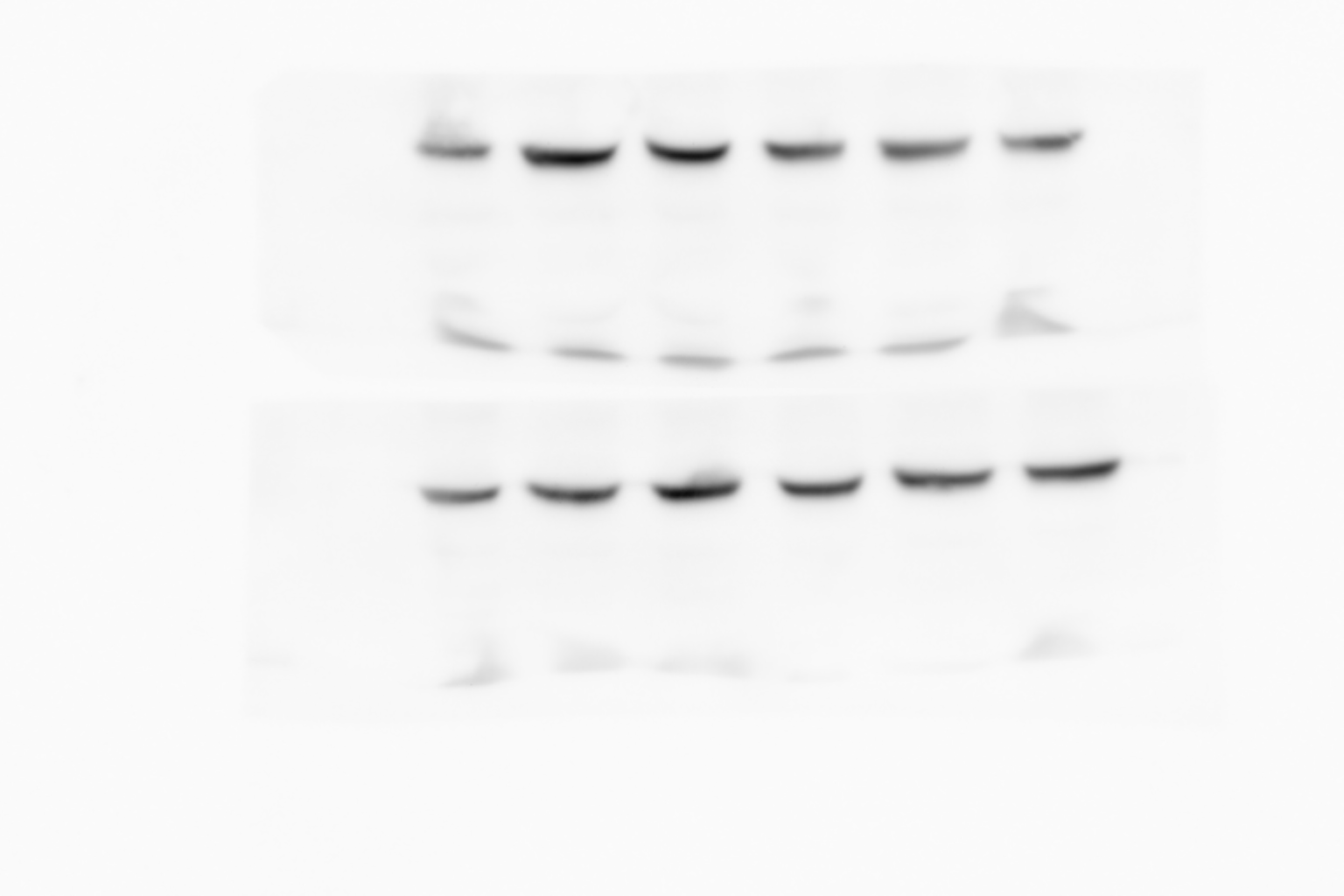

Supplement: Figure 5—source data 3. [file elife-93621-fig5-data3.zip › Figure 5G-hfd1_Pgk1_Bands.tif]

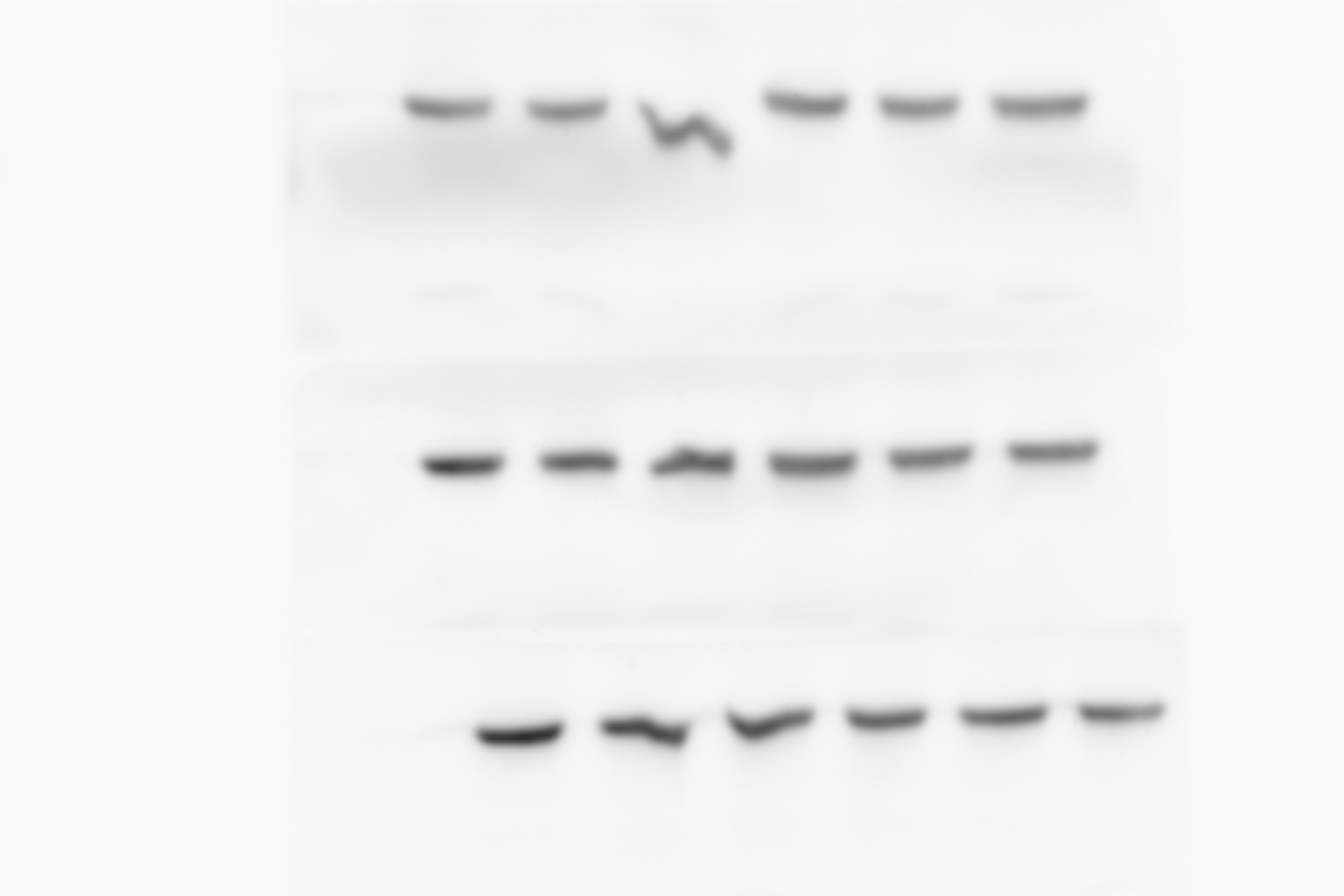

Supplement: Figure 5—source data 3. [file elife-93621-fig5-data3.zip › Figure 5G-TDH3p_Pgk1_Bands.tif]

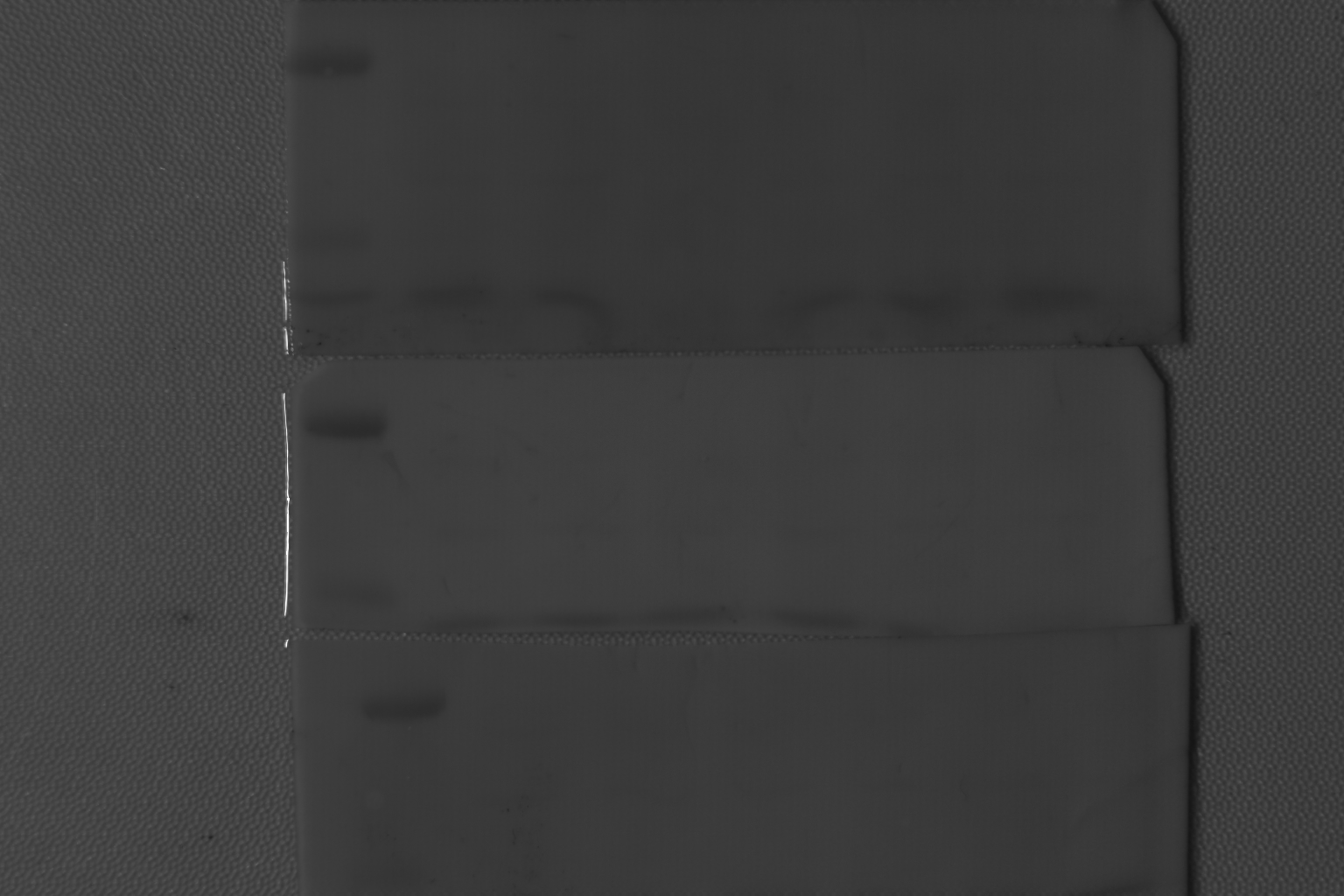

Supplement: Figure 5—source data 3. [file elife-93621-fig5-data3.zip › Figure 5G-TDH3p_Pgk1_Protein ladder.tif]

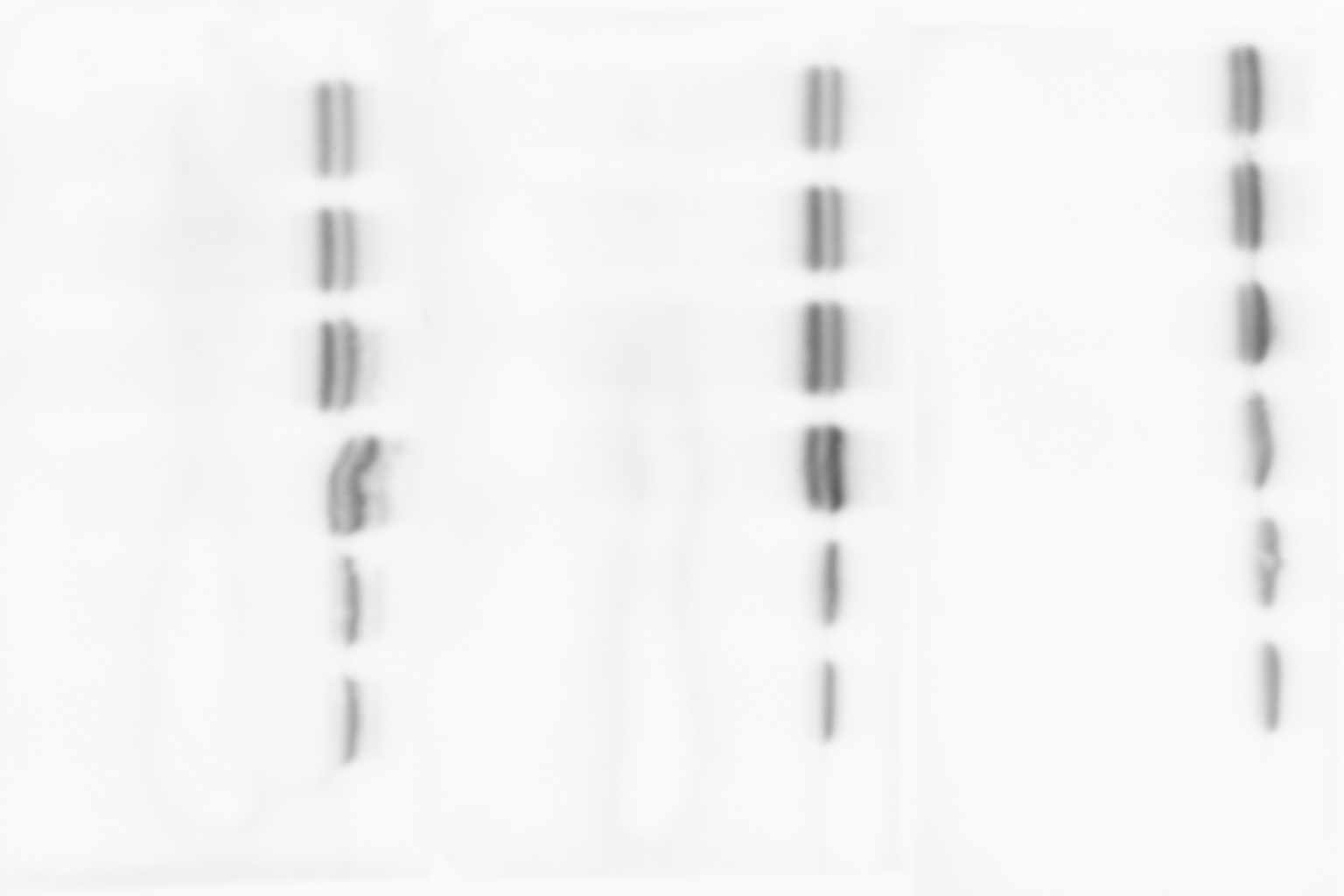

Supplement: Figure 5—source data 3. [file elife-93621-fig5-data3.zip › Figure 5G-TDH3p_TAP_Bands.tif]

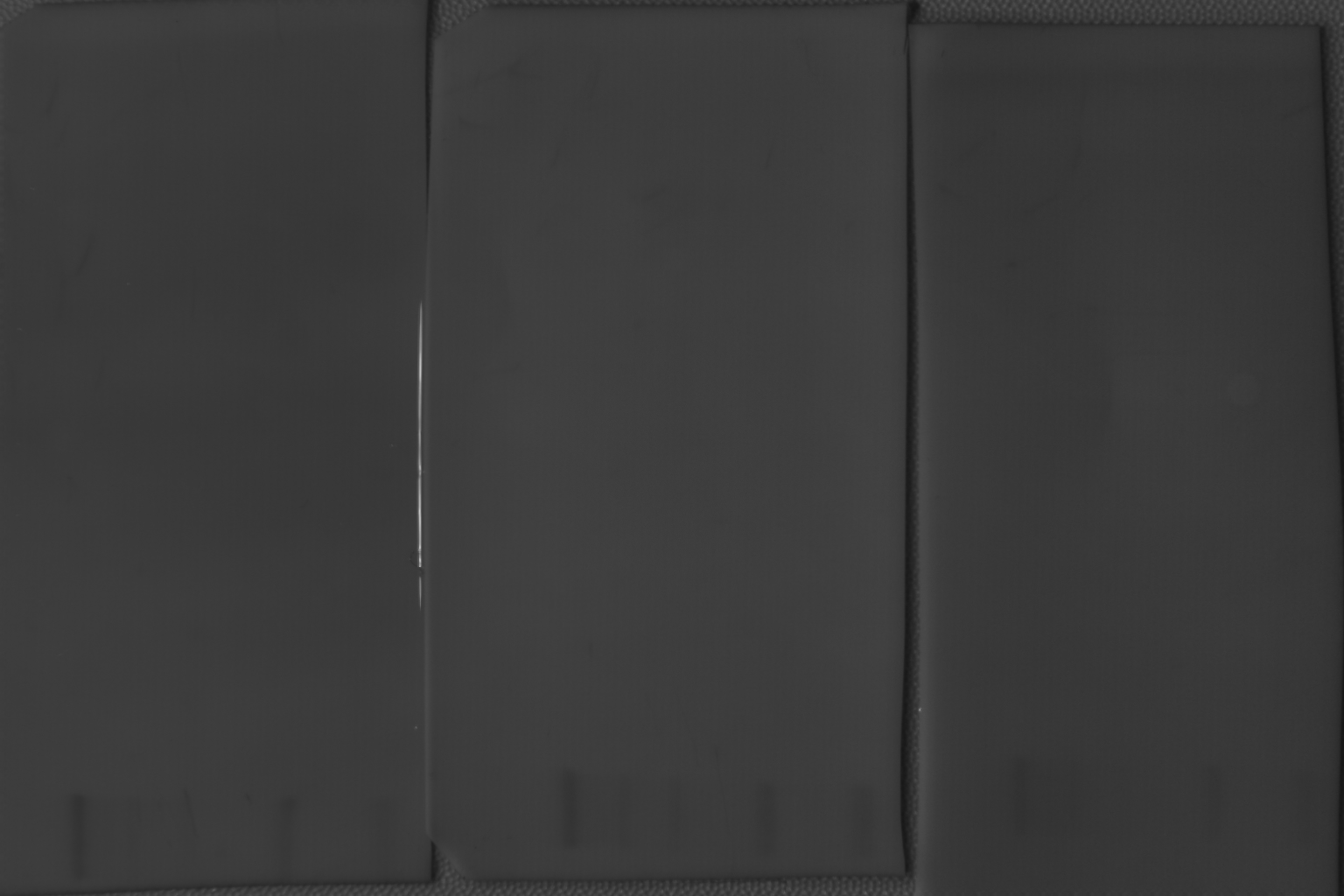

Supplement: Figure 5—source data 3. [file elife-93621-fig5-data3.zip › Figure 5G-TDH3p_TAP_Protein ladder.tif]

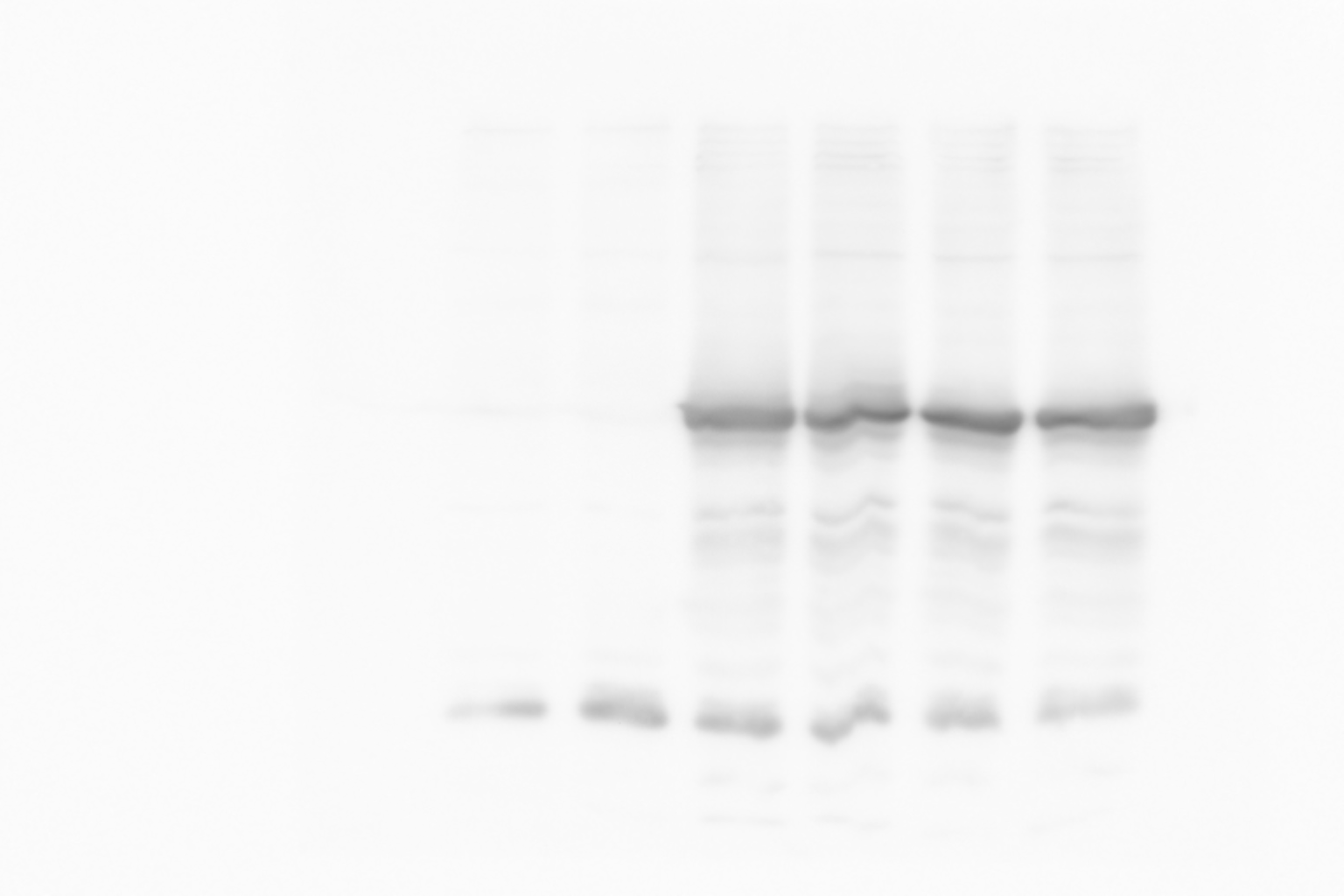

Supplement: Figure 8—source data 2. [file elife-93621-fig8-data2.zip › Figure 8C-Lowerpanel_Input_Bands.tif]

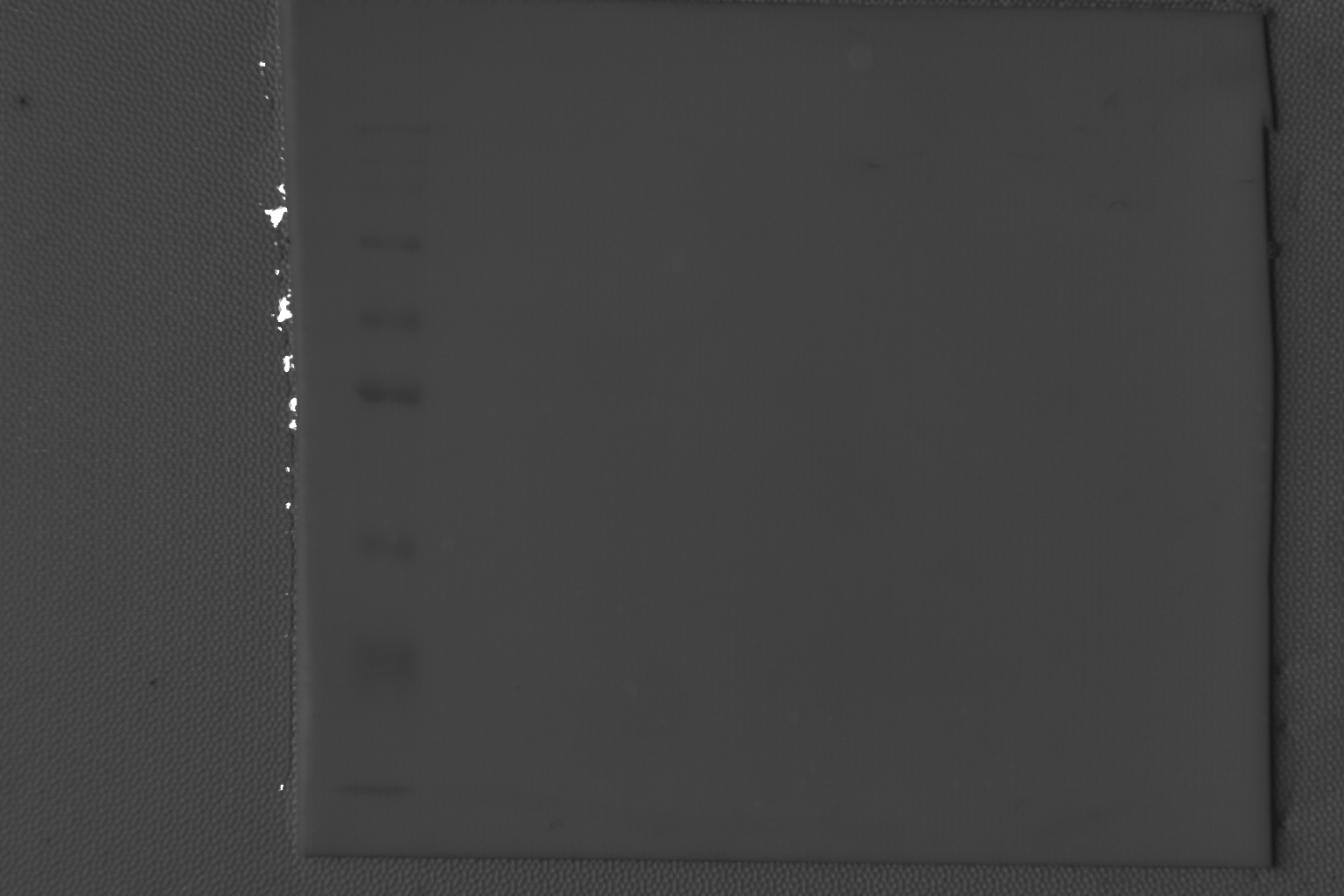

Supplement: Figure 8—source data 2. [file elife-93621-fig8-data2.zip › Figure 8C-Lowerpanel_Input_Protein ladder.tif]

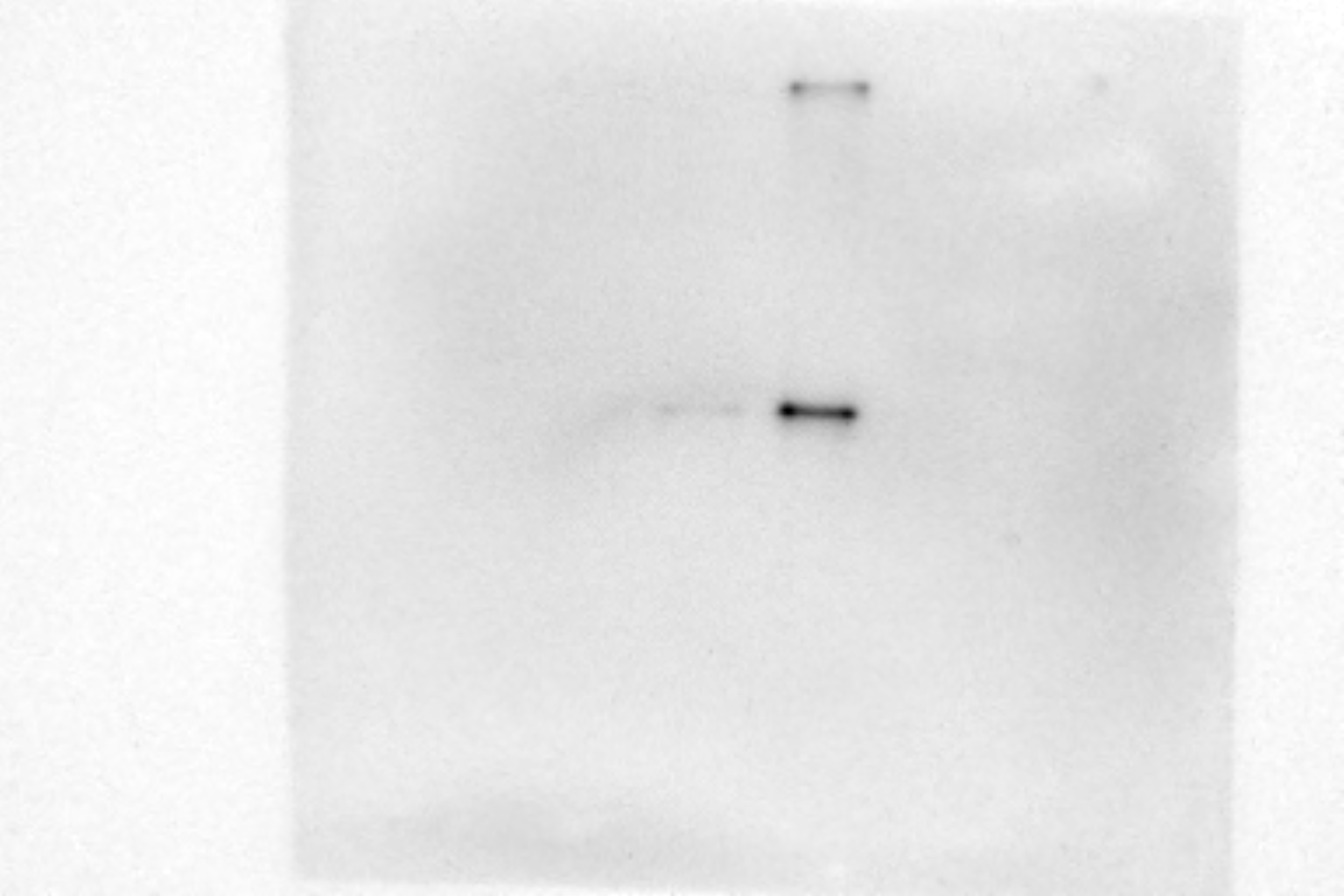

Supplement: Figure 8—source data 2. [file elife-93621-fig8-data2.zip › Figure 8C-Lowerpanel_Pulldown_Bands.tif]

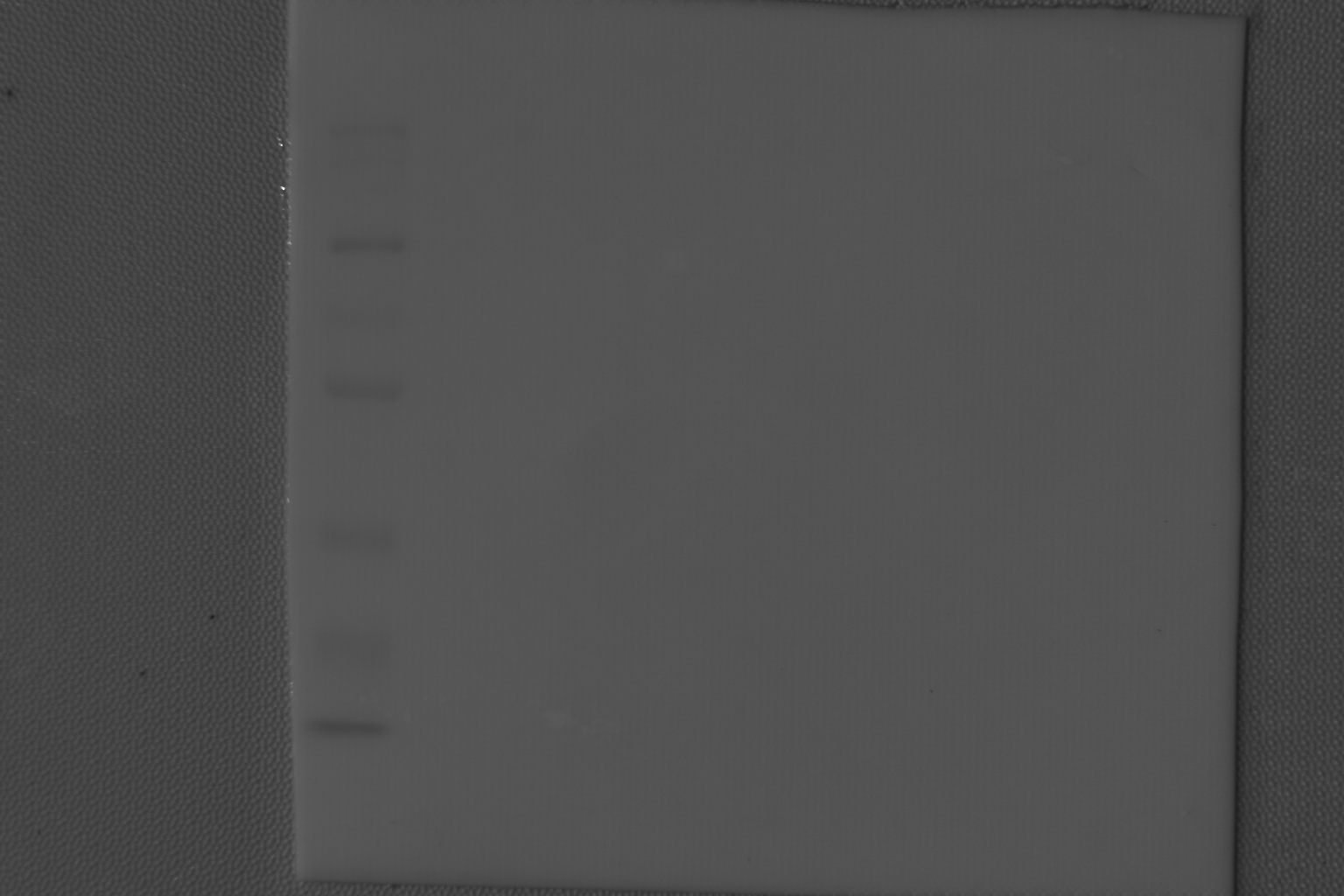

Supplement: Figure 8—source data 2. [file elife-93621-fig8-data2.zip › Figure 8C-Lowerpanel_Pulldown_Protein ladder.tif]

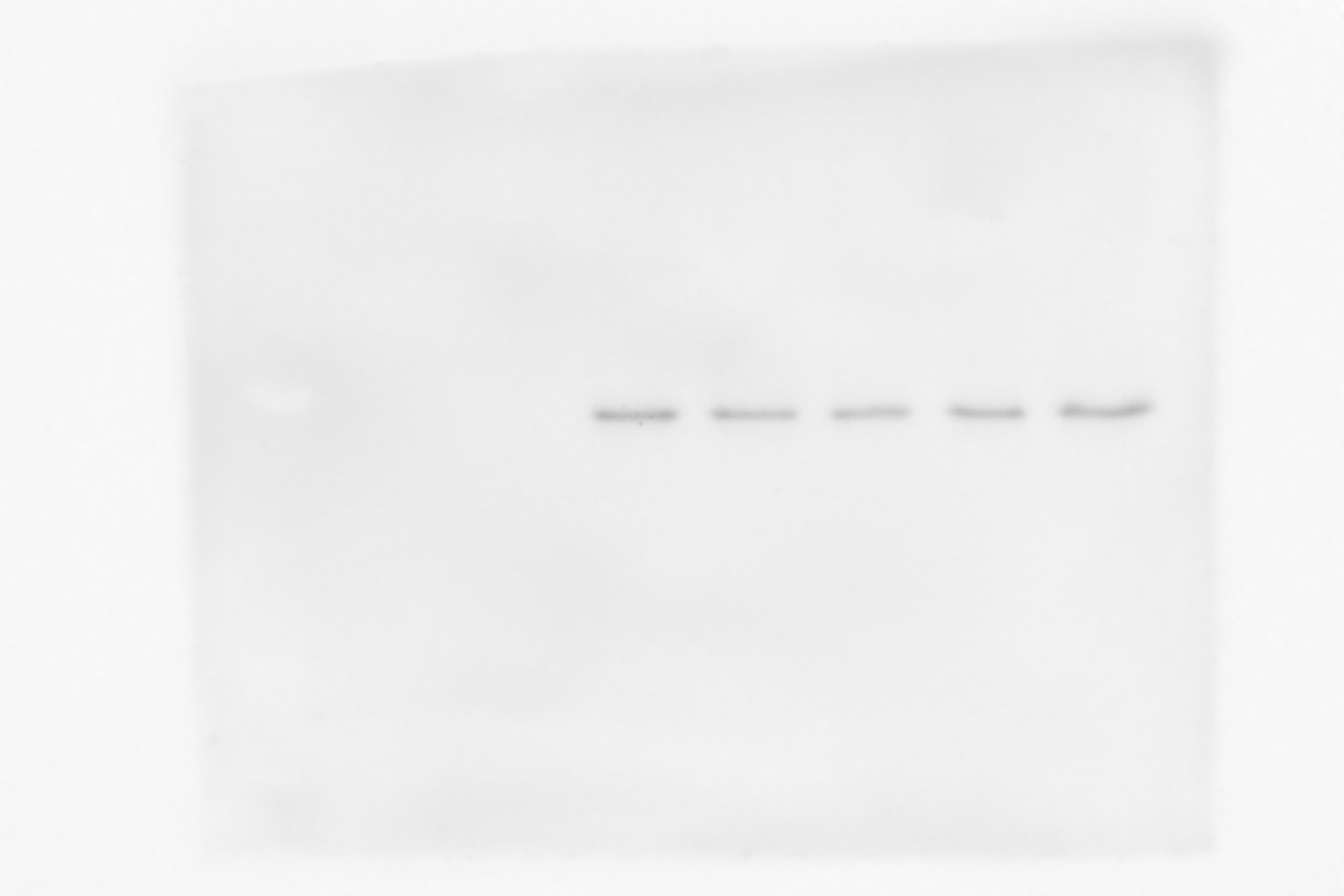

Supplement: Figure 8—source data 2. [file elife-93621-fig8-data2.zip › Figure 8C-Upperpanel_Input_Bands.tif]

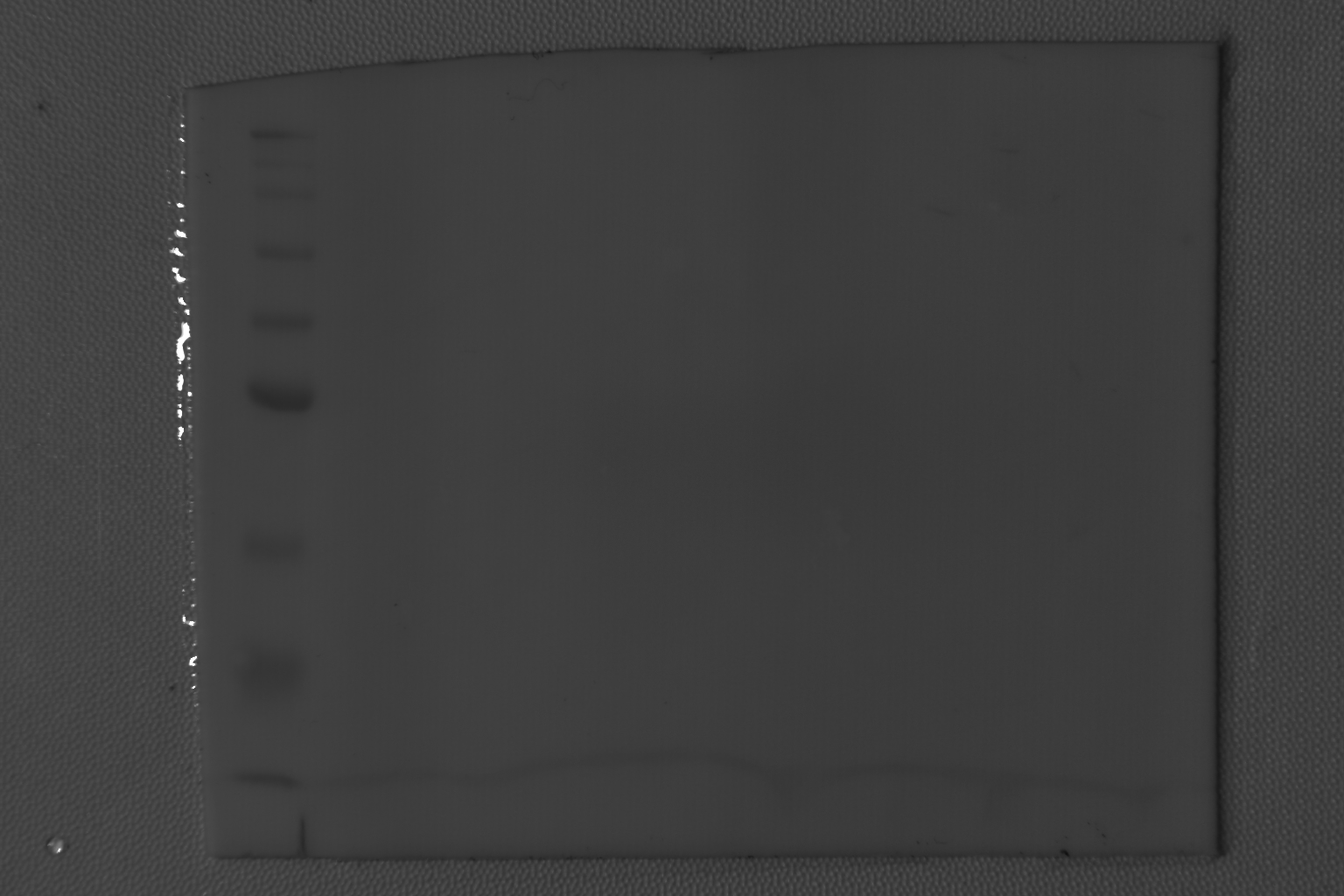

Supplement: Figure 8—source data 2. [file elife-93621-fig8-data2.zip › Figure 8C-Upperpanel_Input_Protein ladder.tif]

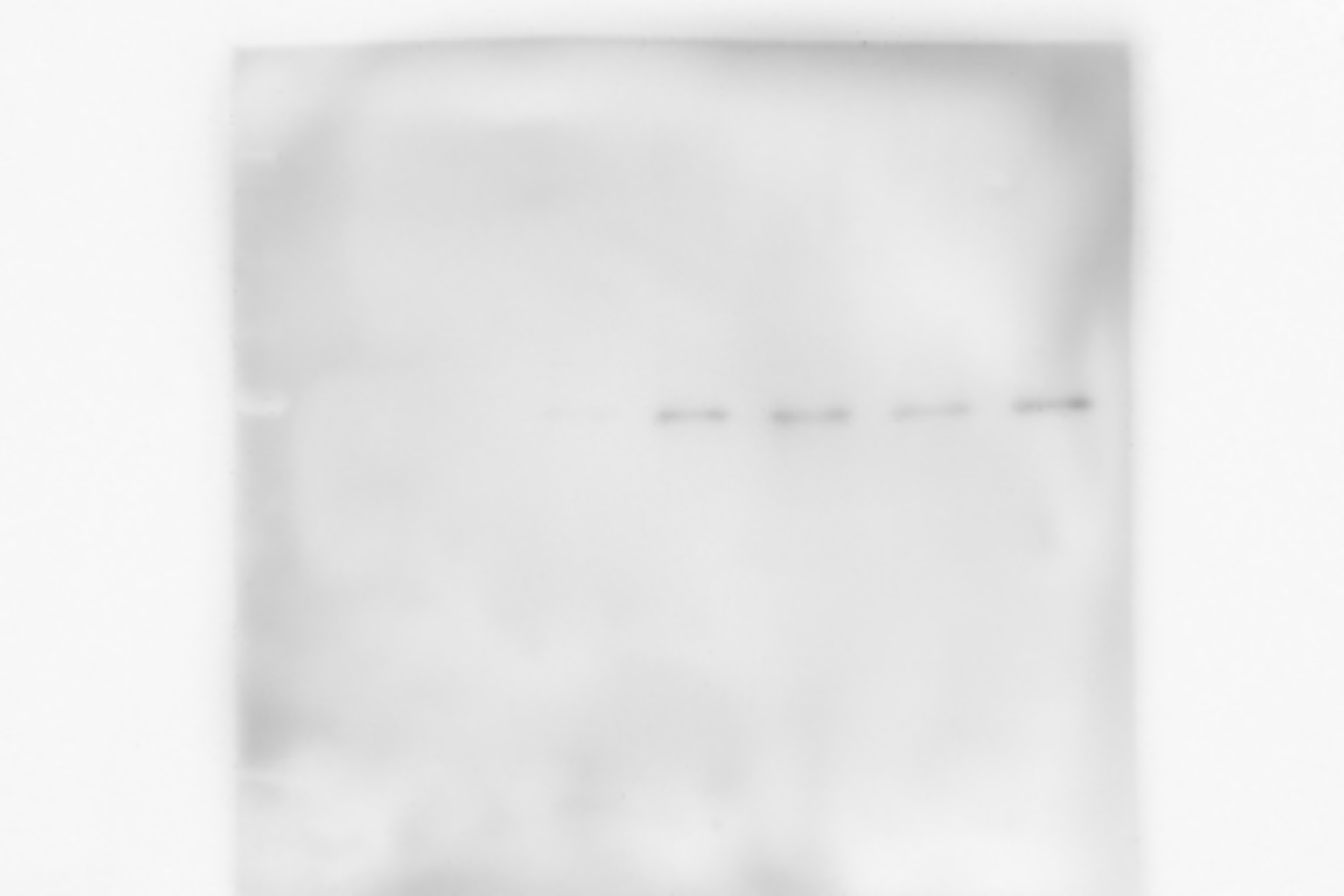

Supplement: Figure 8—source data 2. [file elife-93621-fig8-data2.zip › Figure 8C-Upperpanel_Pulldown_Bands.tif]

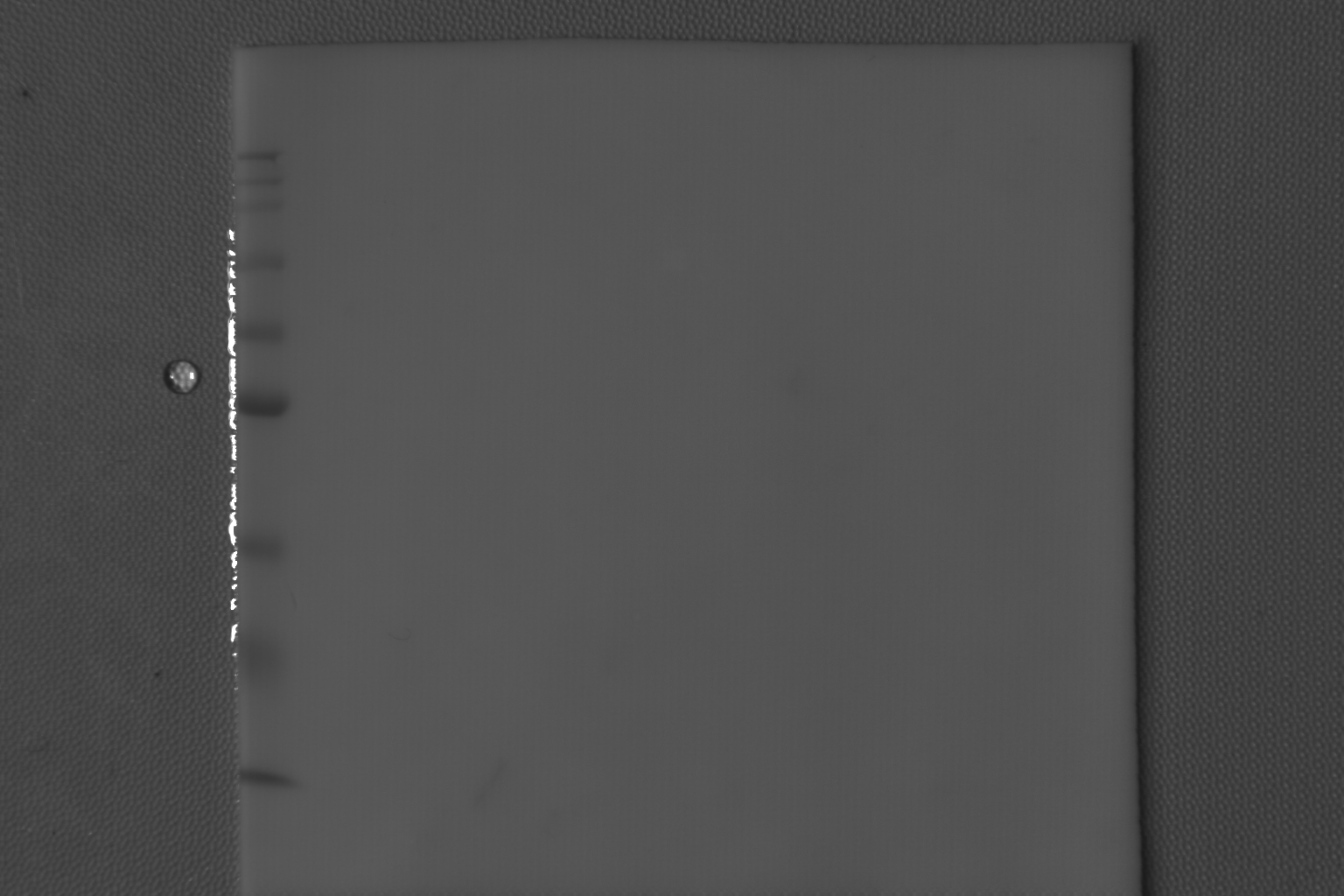

Supplement: Figure 8—source data 2. [file elife-93621-fig8-data2.zip › Figure 8C-Upperpanel_Pulldown_Protein ladder.tif]

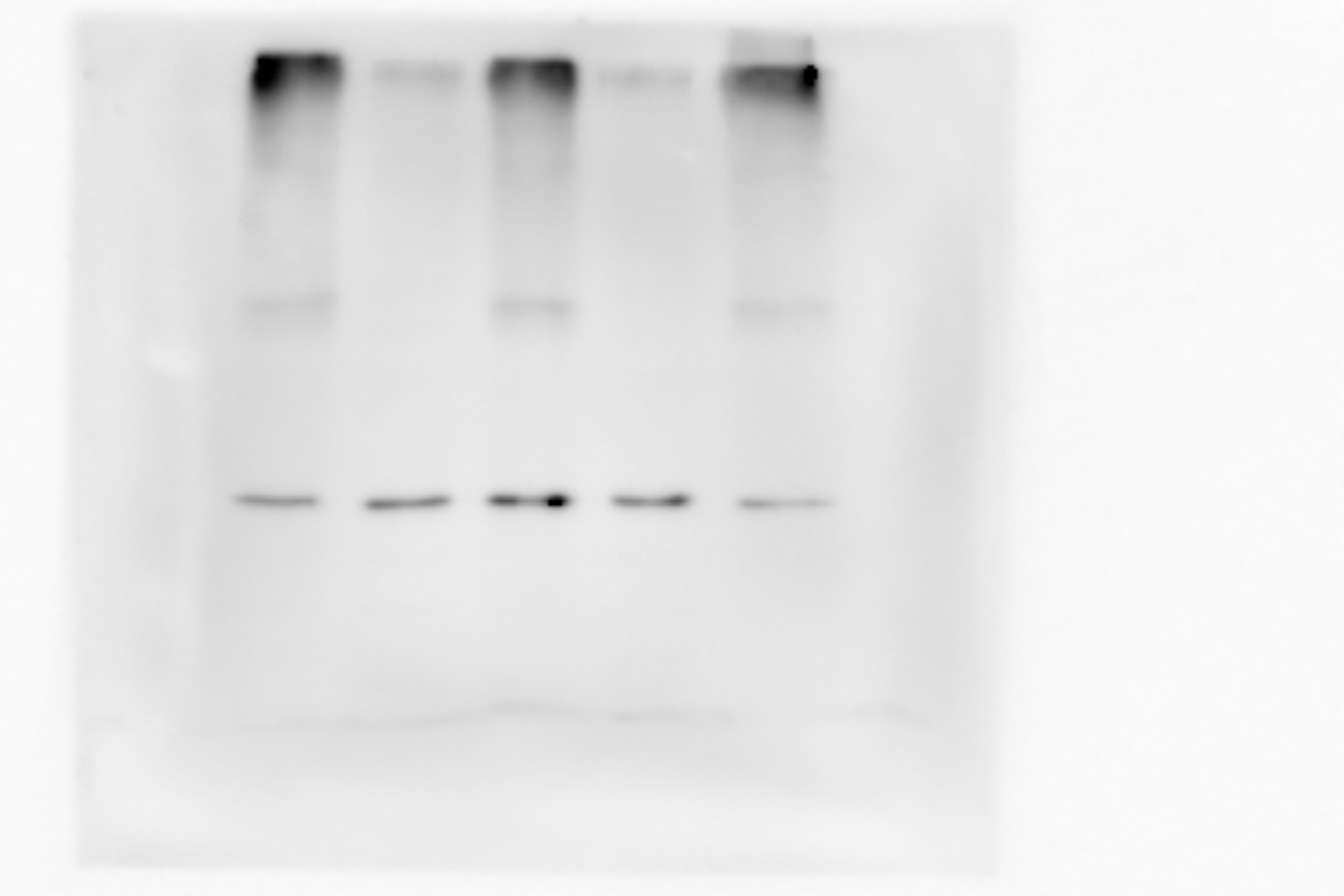

Supplement: Figure 8—source data 2. [file elife-93621-fig8-data2.zip › Figure 8A-Input_HA_Bands.tif]

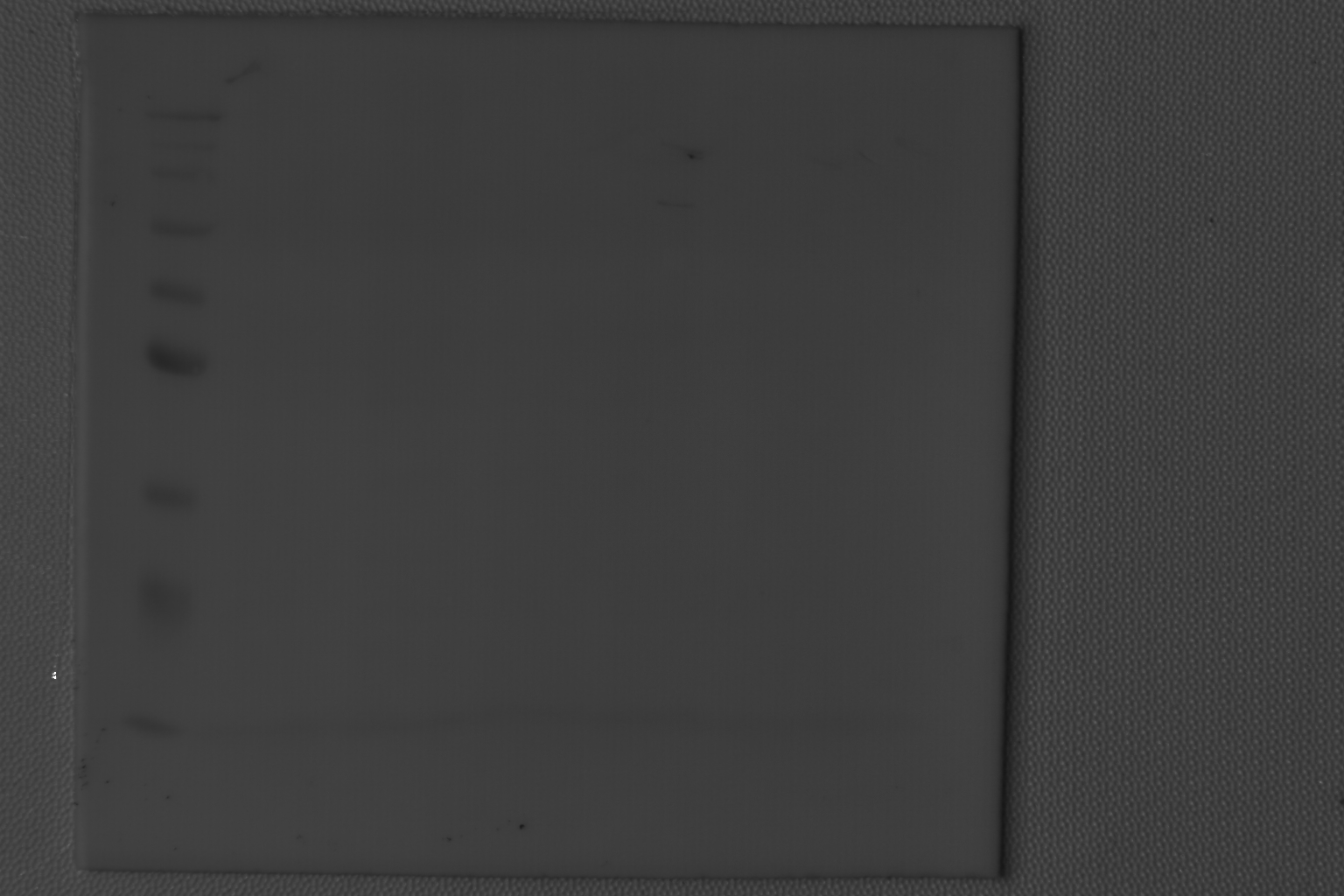

Supplement: Figure 8—source data 2. [file elife-93621-fig8-data2.zip › Figure 8A-Input_HA_Protein ladder.tif]

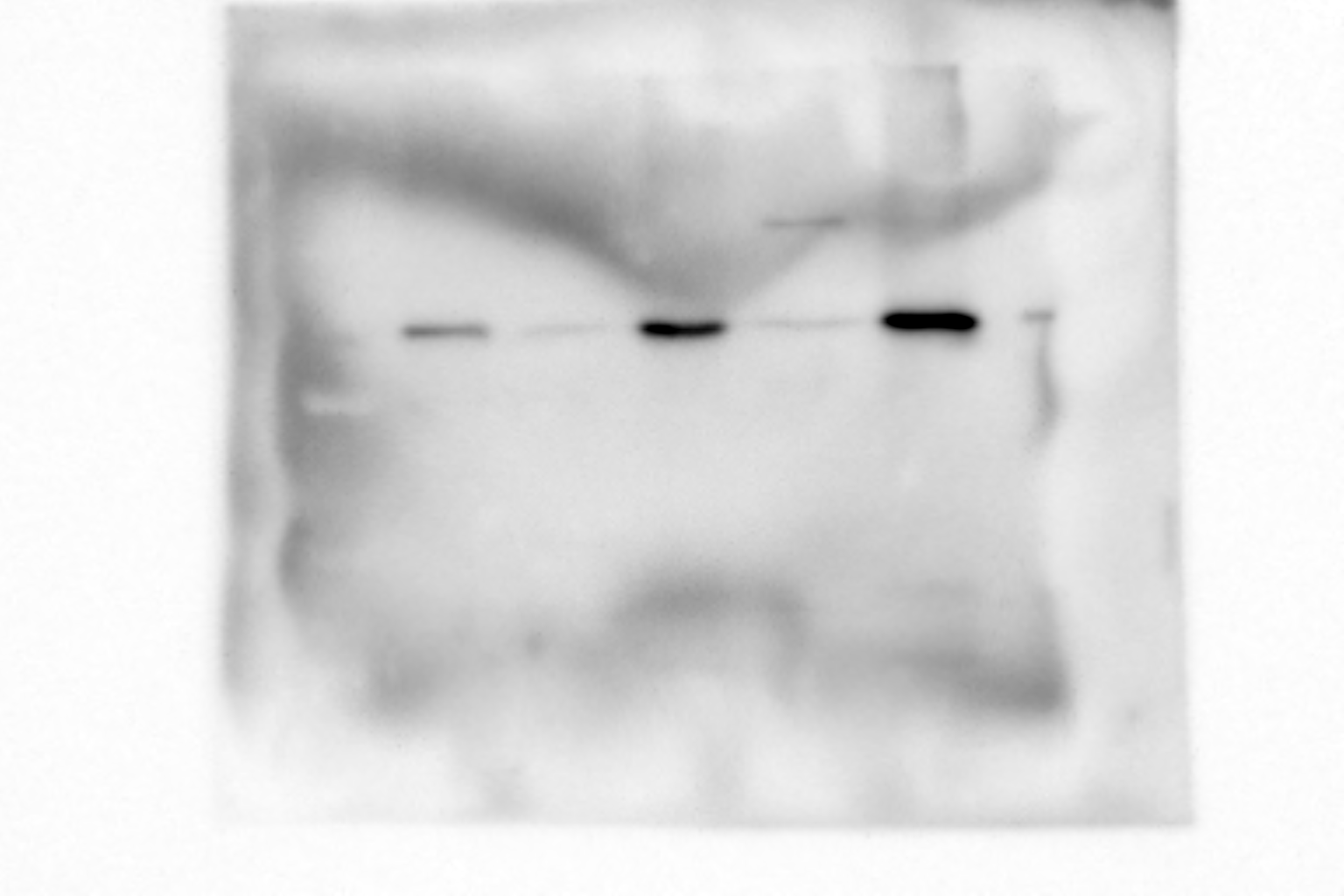

Supplement: Figure 8—source data 2. [file elife-93621-fig8-data2.zip › Figure 8A-Pulldown_HA_Bands.tif]

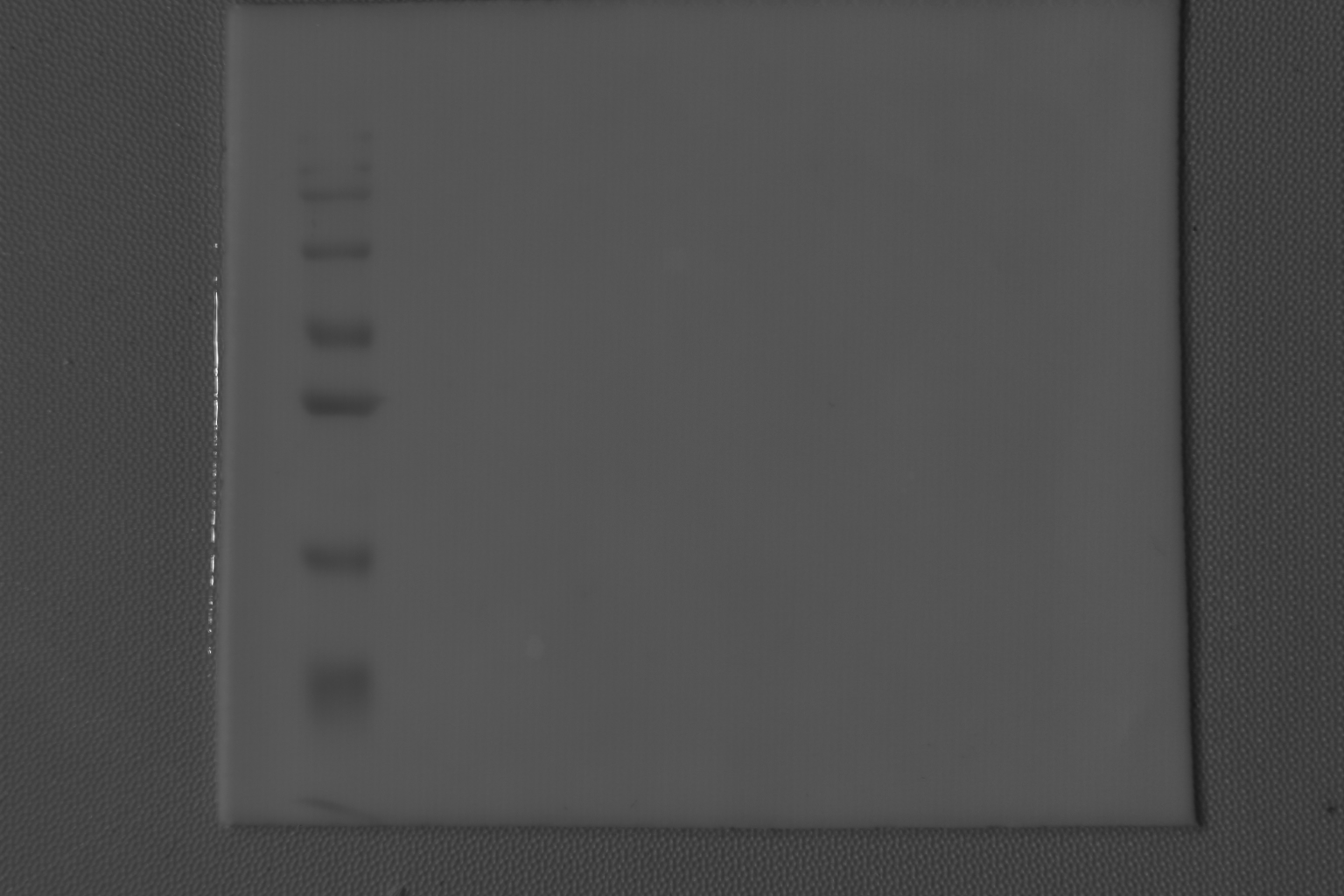

Supplement: Figure 8—source data 2. [file elife-93621-fig8-data2.zip › Figure 8A-Pulldown_HA_Protein ladder.tif]

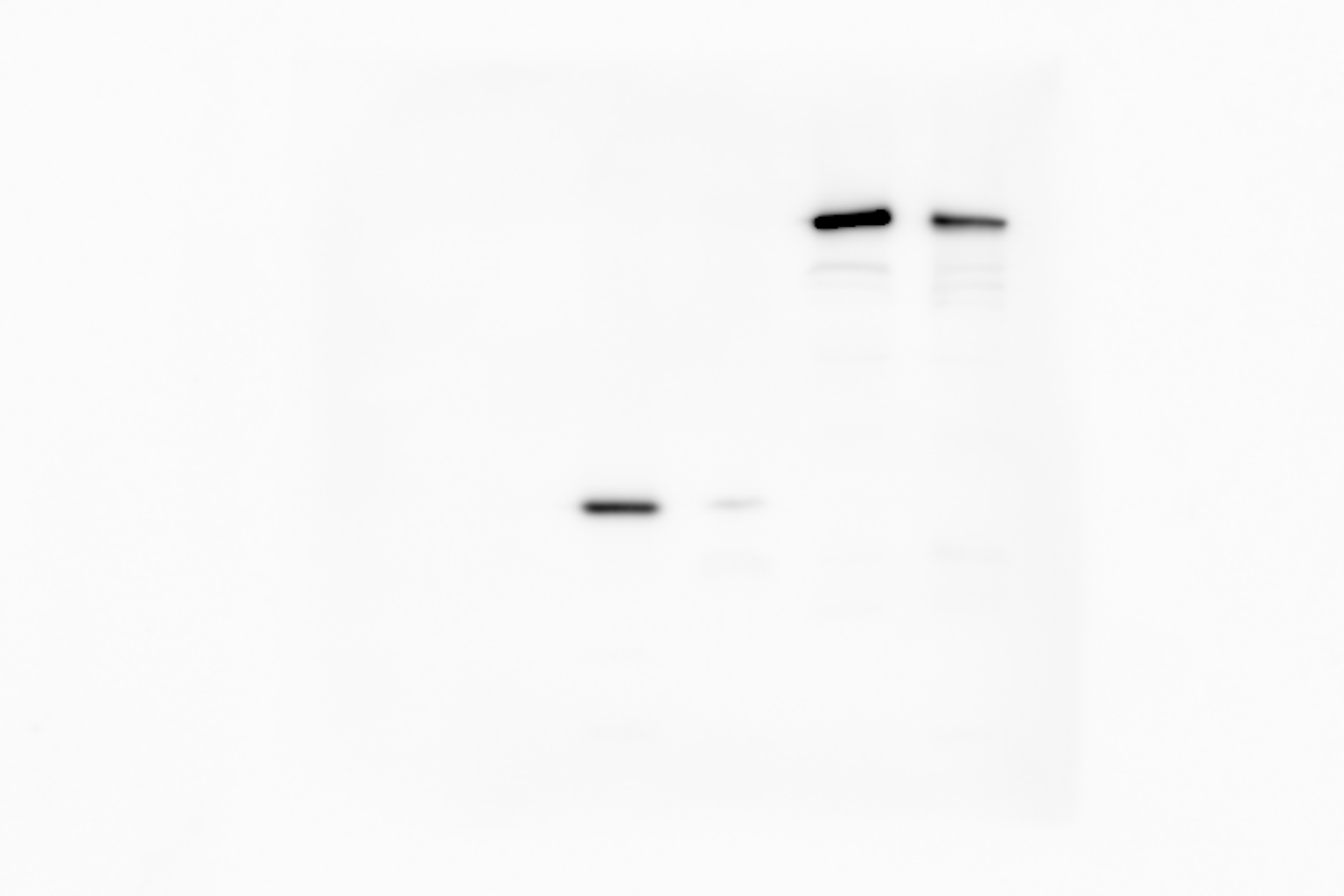

Supplement: Figure 8—source data 2. [file elife-93621-fig8-data2.zip › Figure 8A-Pulldown_TAP_Bands.tif]

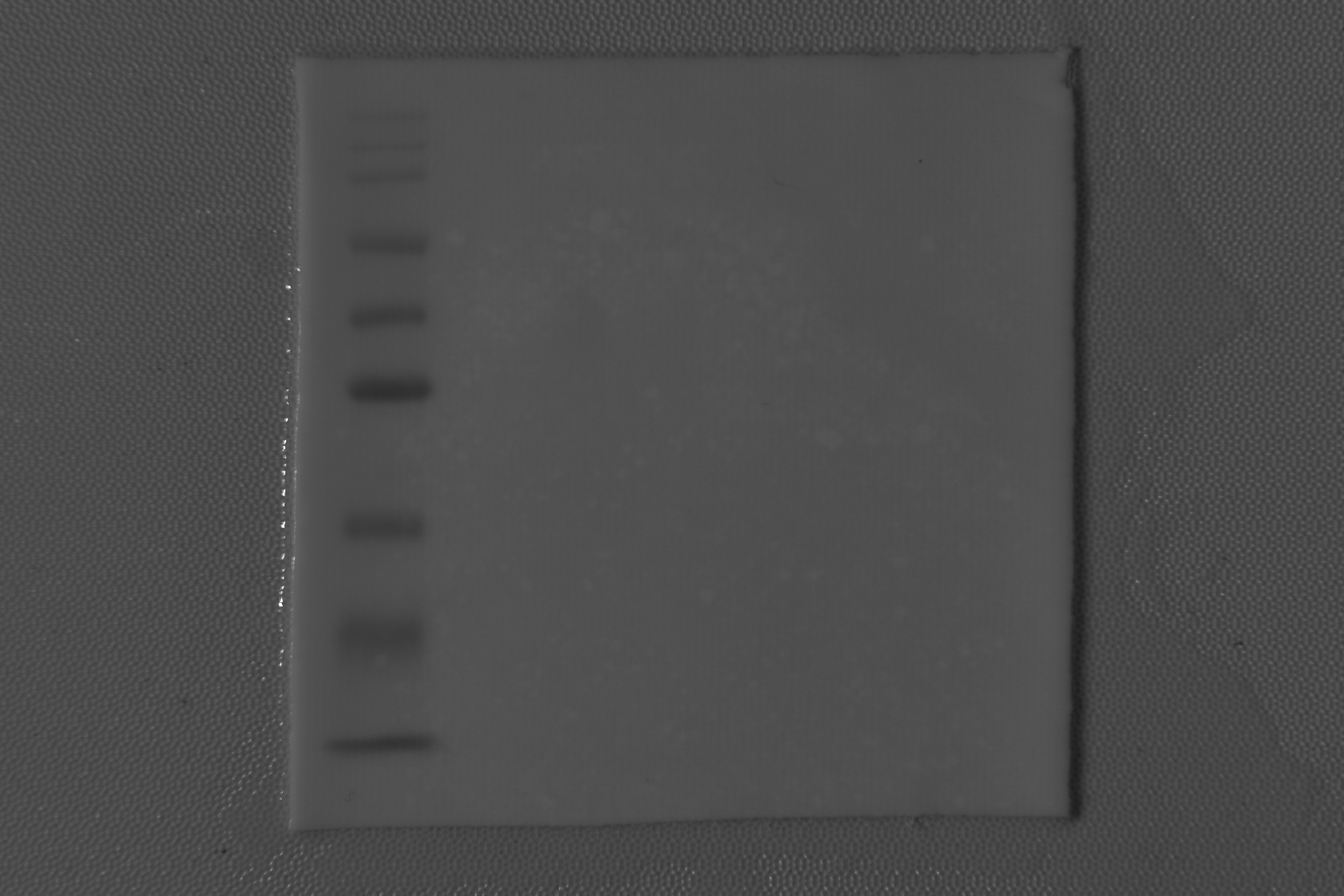

Supplement: Figure 8—source data 2. [file elife-93621-fig8-data2.zip › Figure 8A-Pulldown_TAP_Protein ladder.tif]

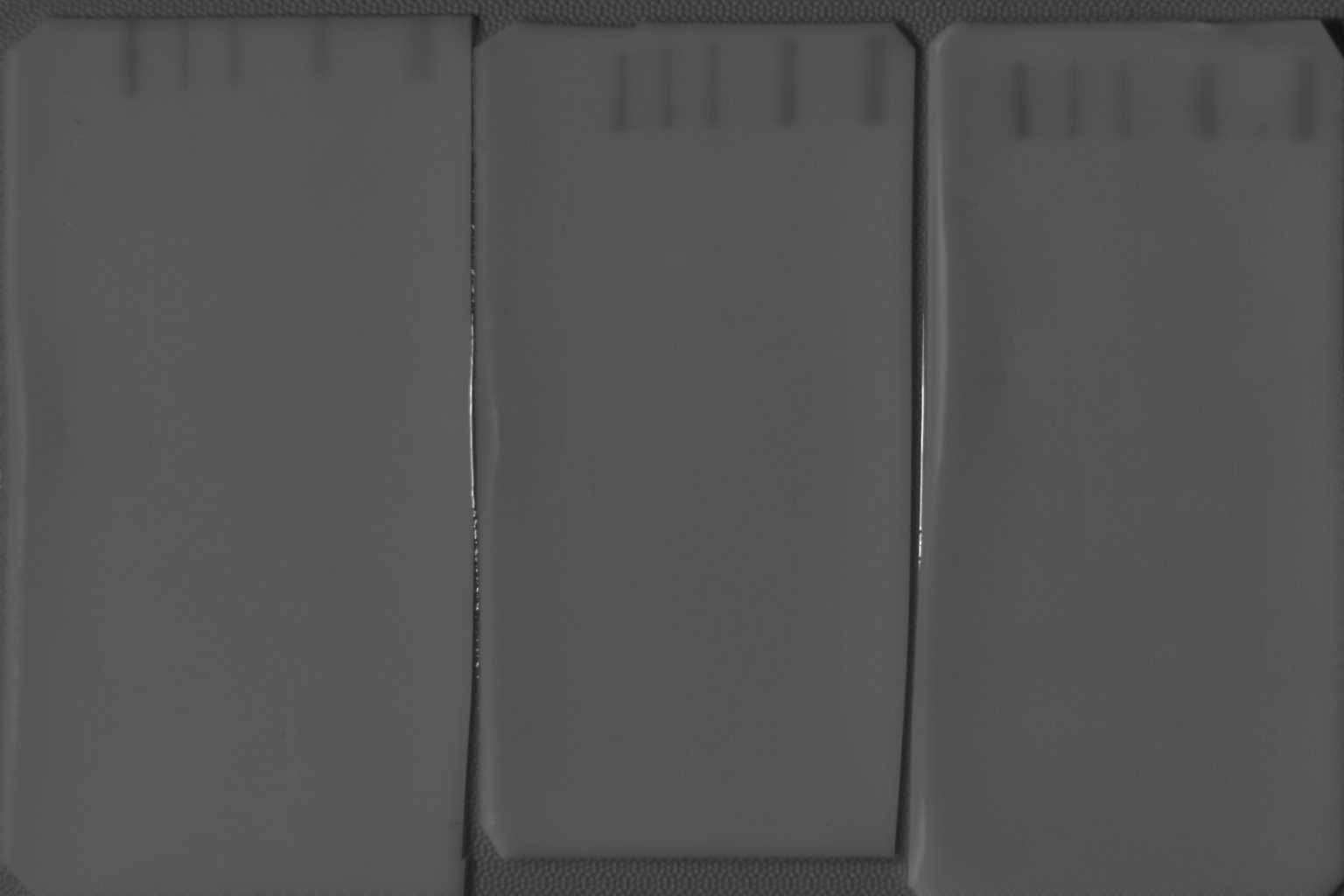

Supplement: Figure 8—figure supplement 1—source data 2. [file elife-93621-fig8-figsupp1-data2.zip › Figure 8 - Figure Supplement 1-WT_TAP_Protein ladder.tif]

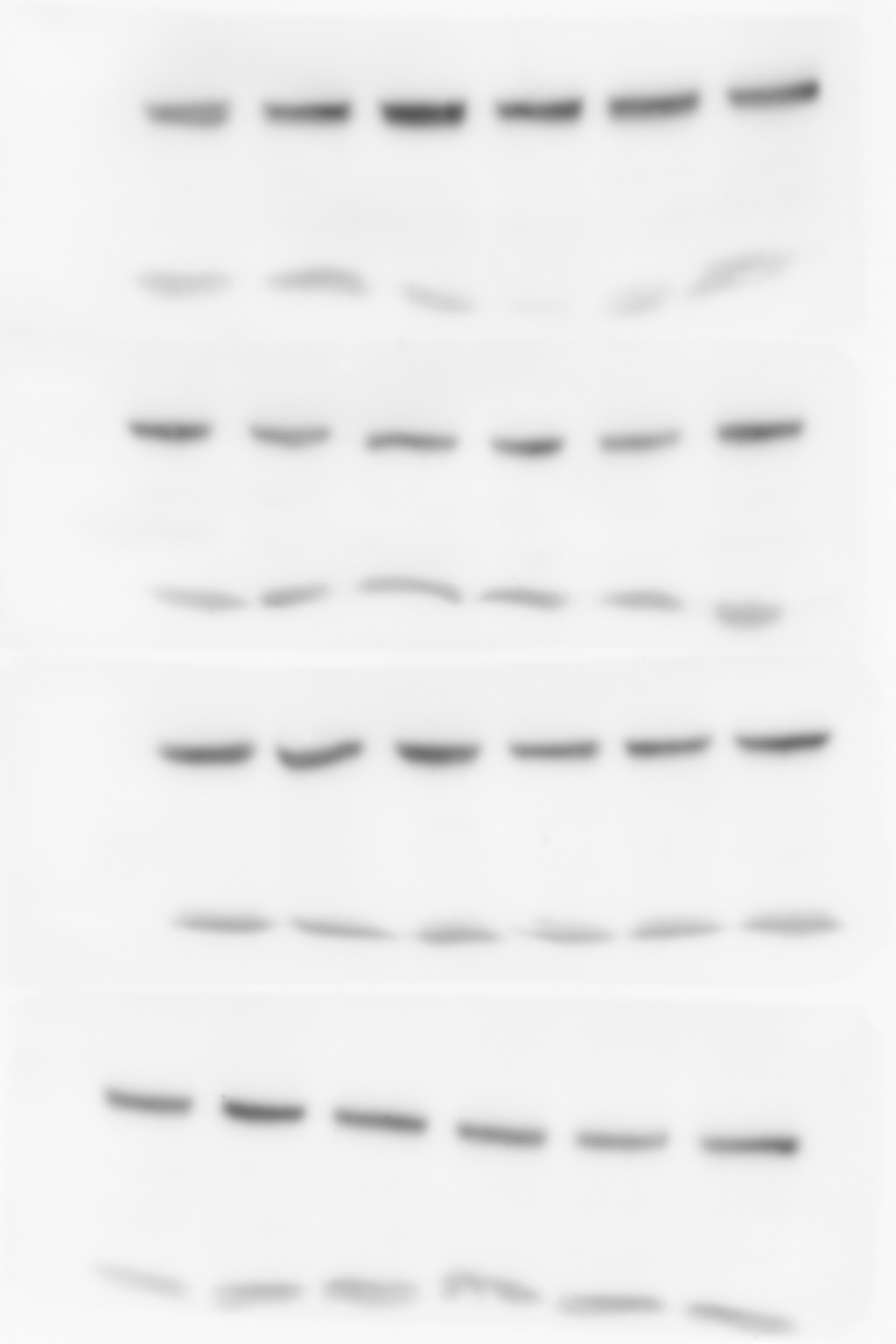

Supplement: Figure 8—figure supplement 1—source data 2. [file elife-93621-fig8-figsupp1-data2.zip › Figure 8 - Figure Supplement 1-tom20_Pgk1_Bands.tif]

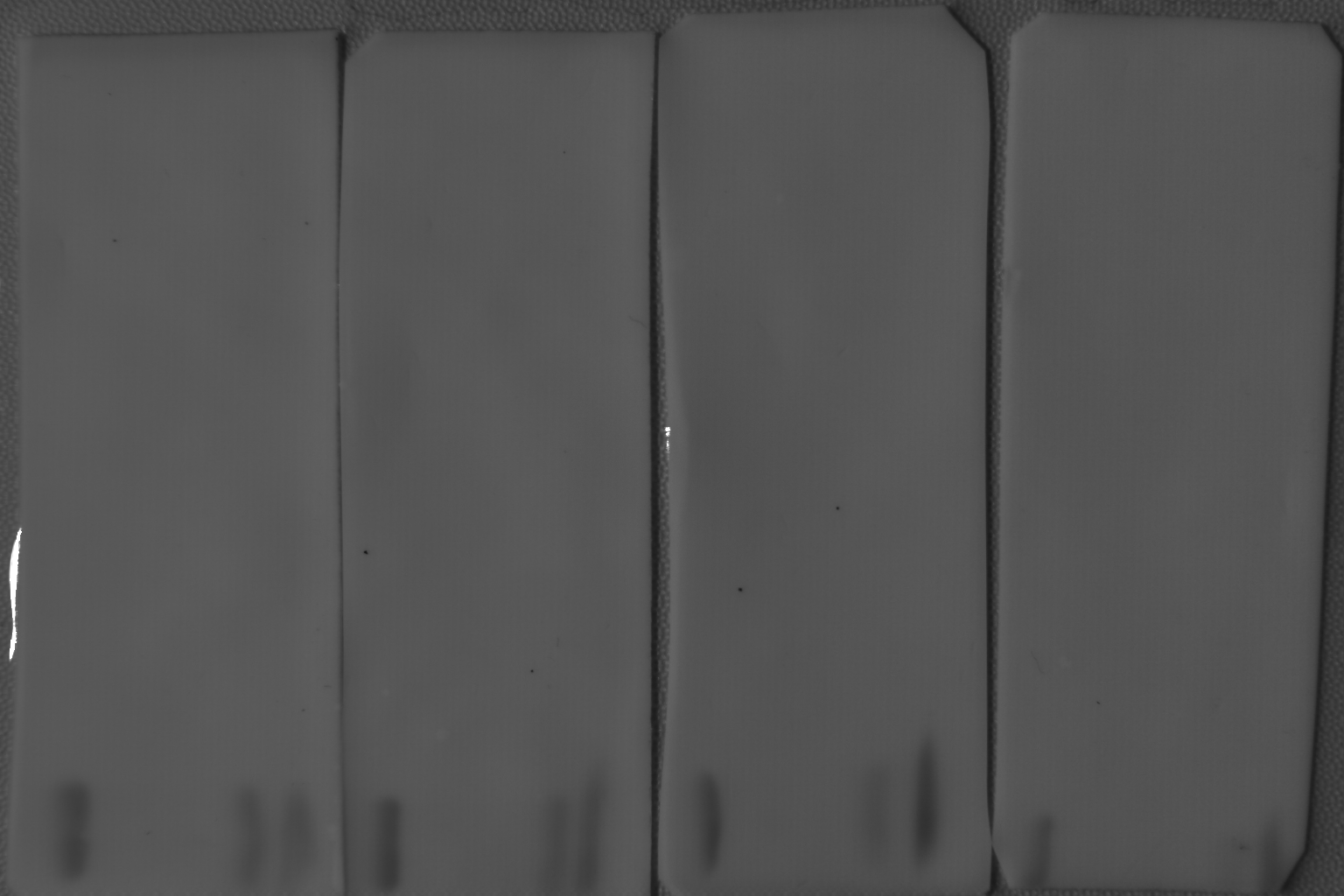

Supplement: Figure 8—figure supplement 1—source data 2. [file elife-93621-fig8-figsupp1-data2.zip › Figure 8 - Figure Supplement 1-tom20_Pgk1_Protein ladder.tif]

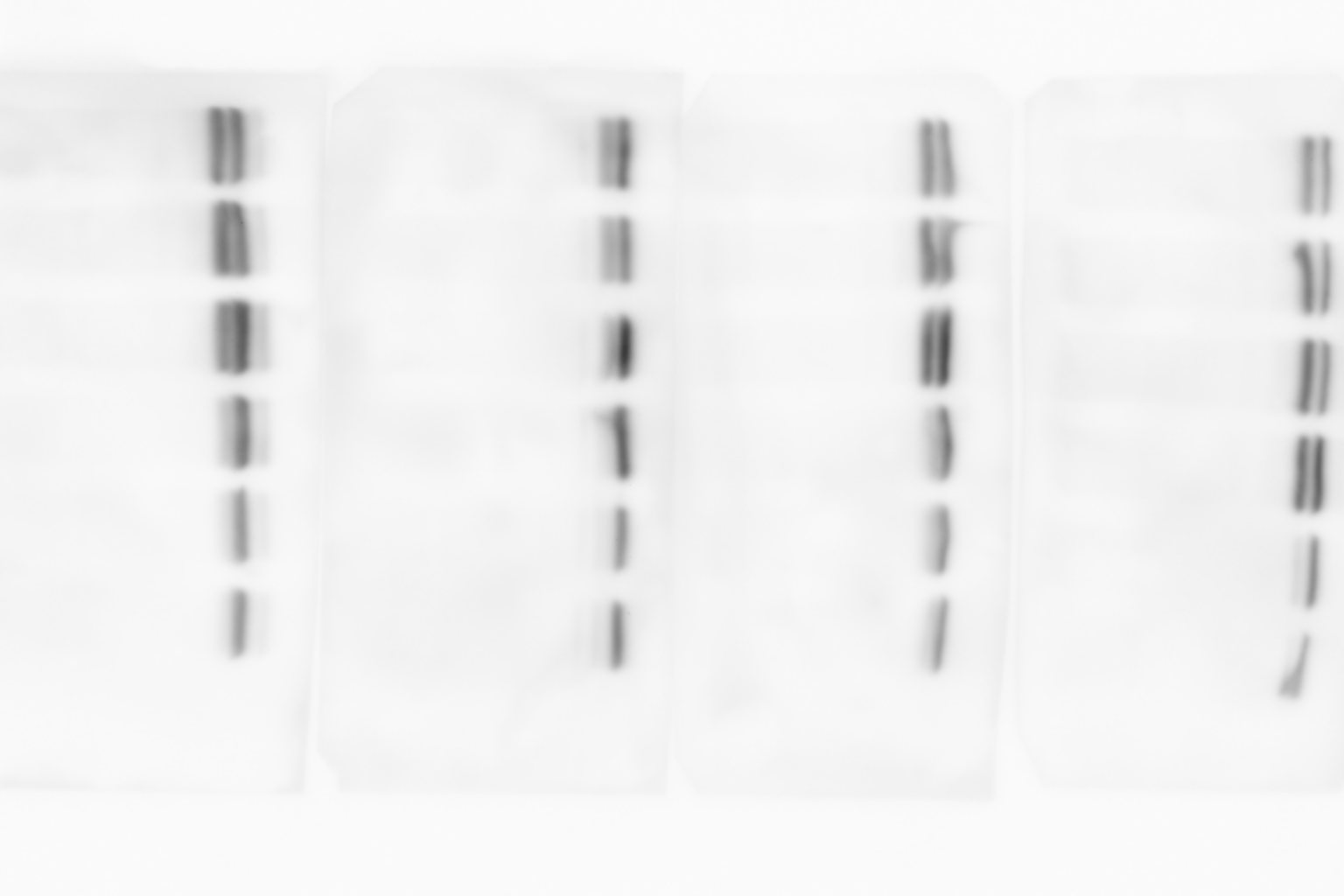

Supplement: Figure 8—figure supplement 1—source data 2. [file elife-93621-fig8-figsupp1-data2.zip › Figure 8 - Figure Supplement 1-tom20_TAP_Bands.tif]

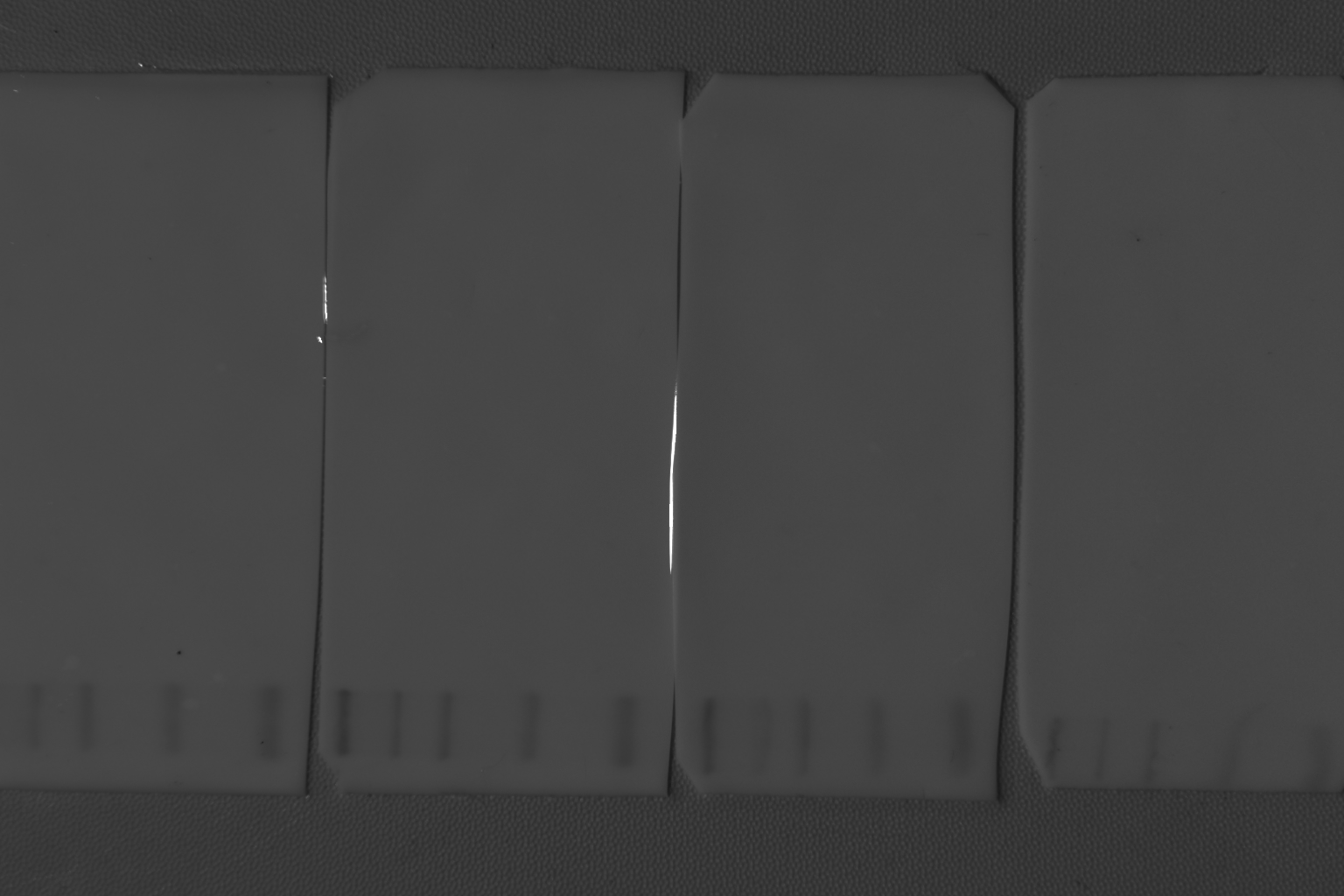

Supplement: Figure 8—figure supplement 1—source data 2. [file elife-93621-fig8-figsupp1-data2.zip › Figure 8 - Figure Supplement 1-tom20_TAP_Protein ladder.tif]

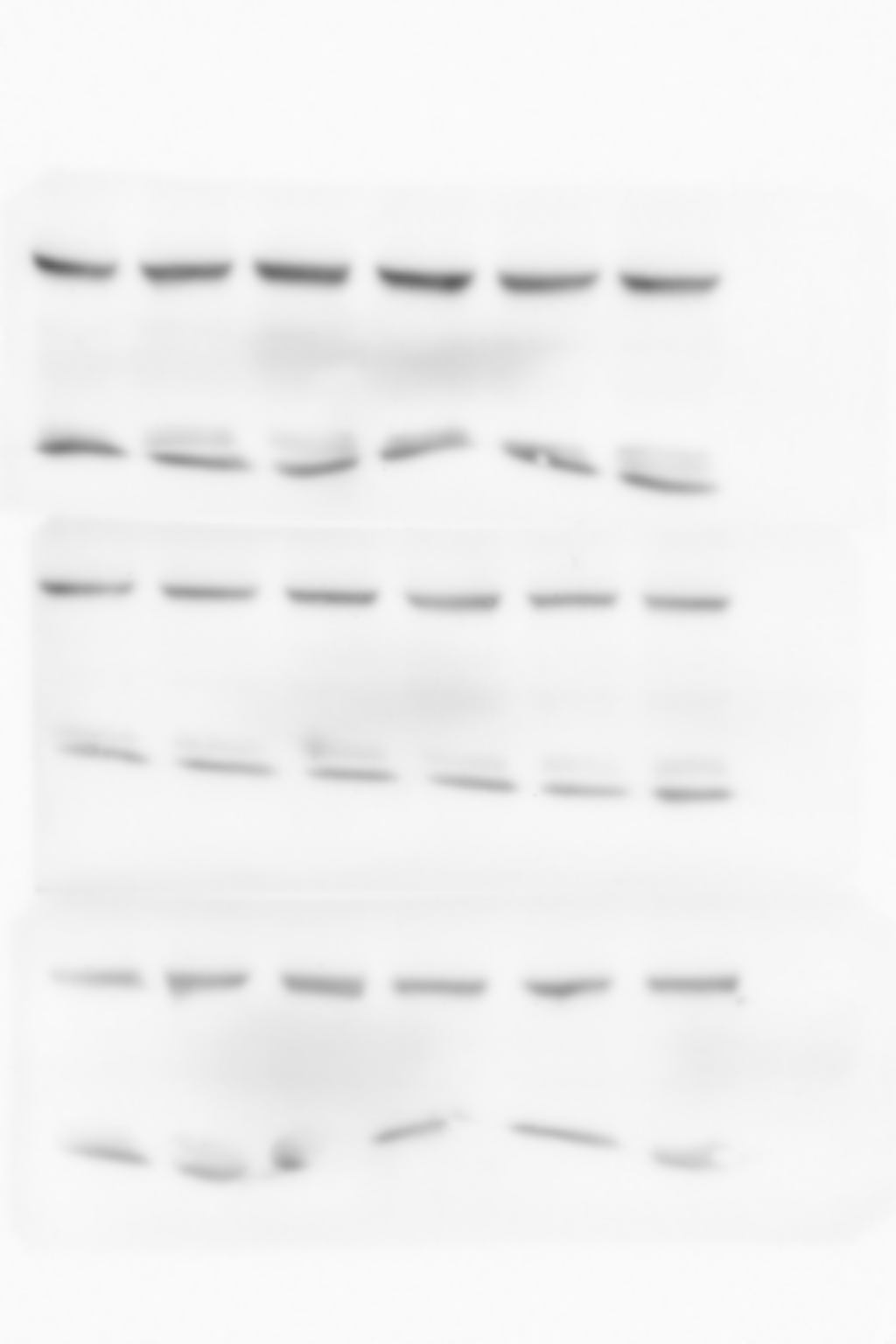

Supplement: Figure 8—figure supplement 1—source data 2. [file elife-93621-fig8-figsupp1-data2.zip › Figure 8 - Figure Supplement 1-WT_Pgk1_Bands.tif]

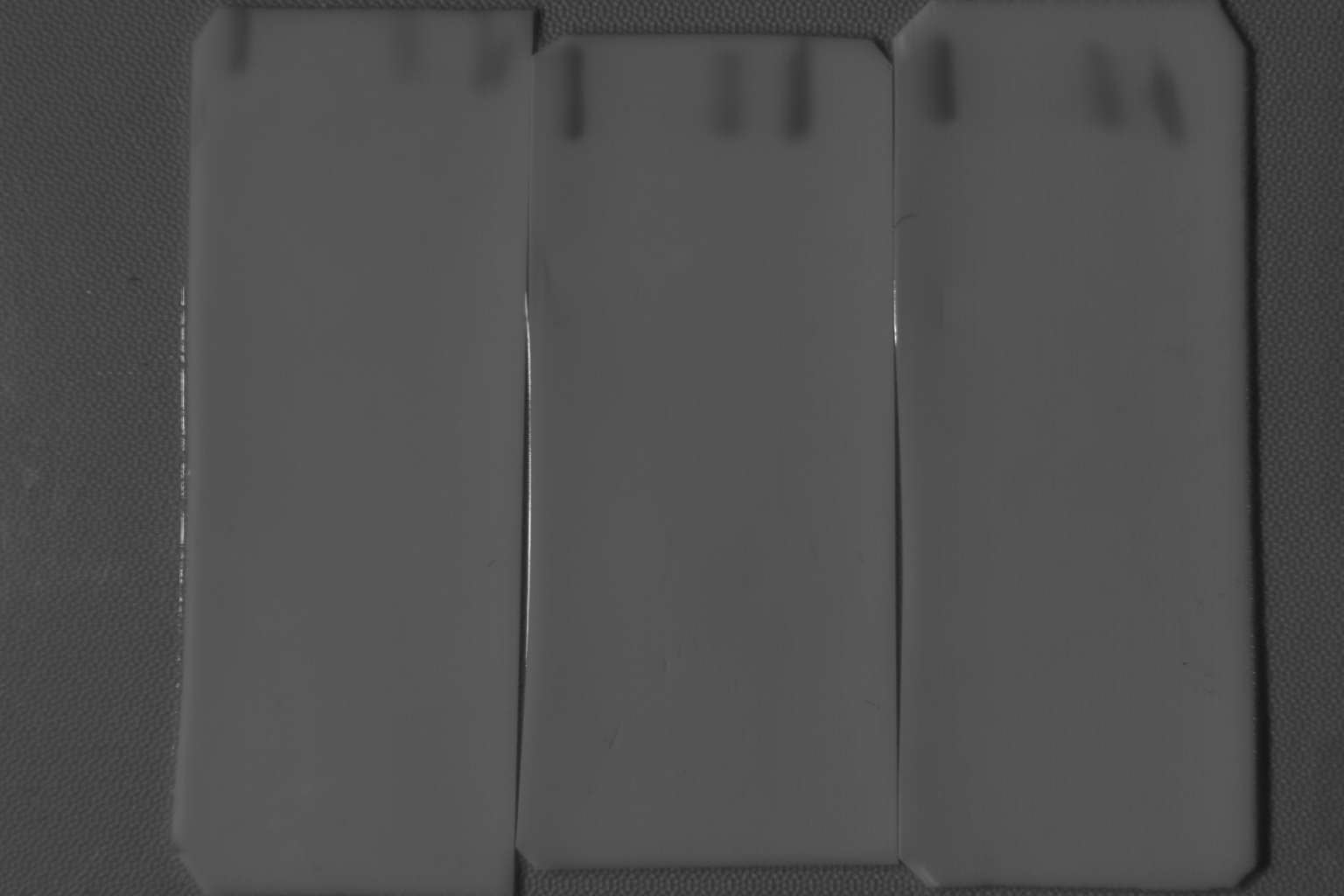

Supplement: Figure 8—figure supplement 1—source data 2. [file elife-93621-fig8-figsupp1-data2.zip › Figure 8 - Figure Supplement 1-WT_Pgk1_Protein ladder.tif]

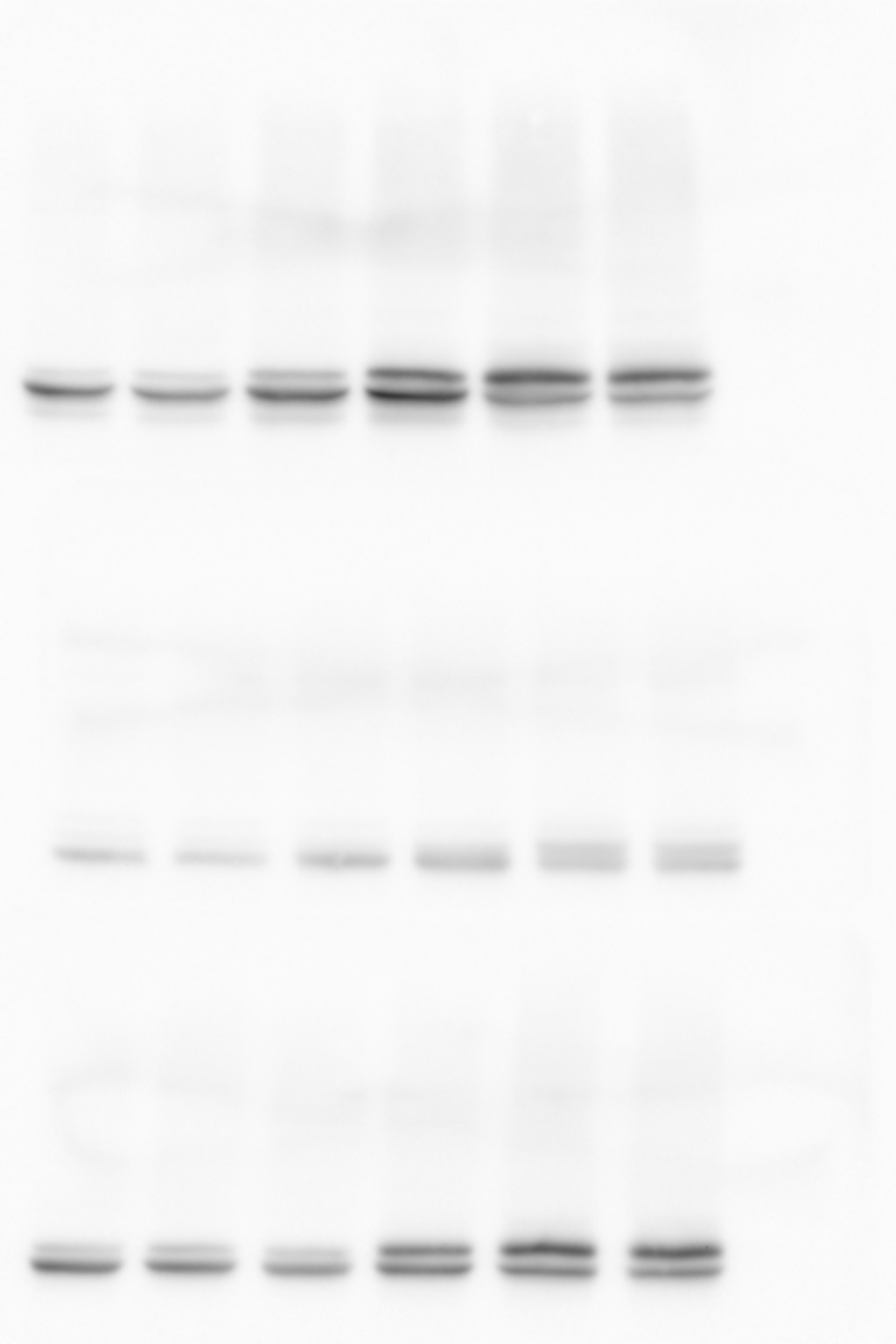

Supplement: Figure 8—figure supplement 1—source data 2. [file elife-93621-fig8-figsupp1-data2.zip › Figure 8 - Figure Supplement 1-WT_TAP_Bands.tif]
