# Supplementary material for: Inhibition of mitochondrial protein import and proteostasis by a pro-apoptotic lipid
Source: eLife. 2025 May 30;13:RP93621. doi: 10.7554/eLife.93621 (PMC12124835; doi:10.7554/eLife.93621)
Supplement: Figure 5—source data 1. [file elife-93621-fig5-data1.pdf]

Figure 5A - Aim17

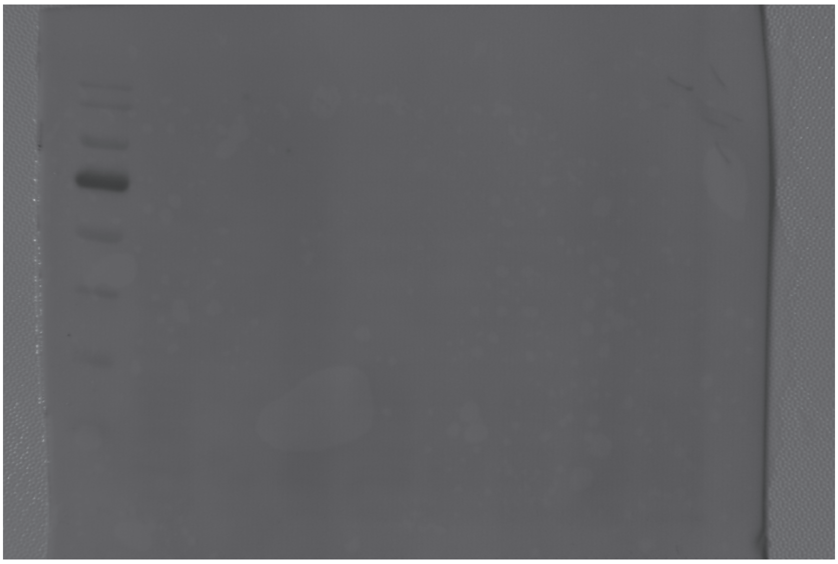

Protein ladder

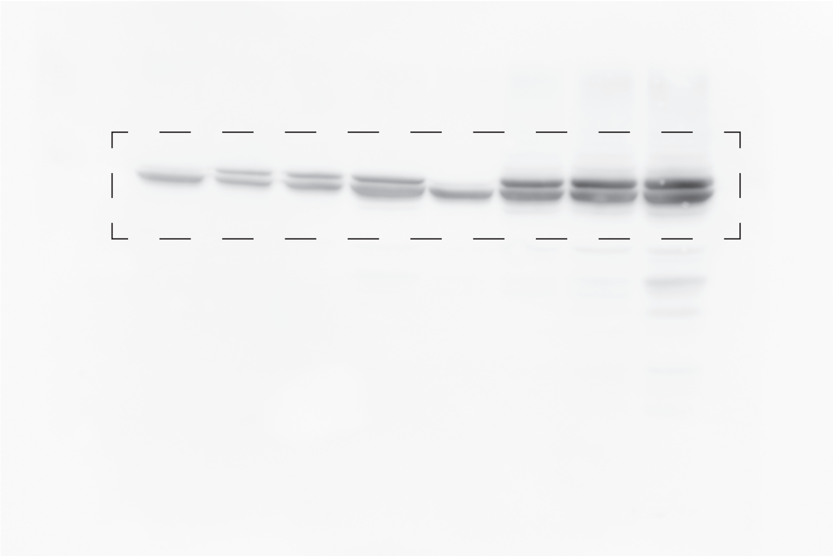

Relevant bands

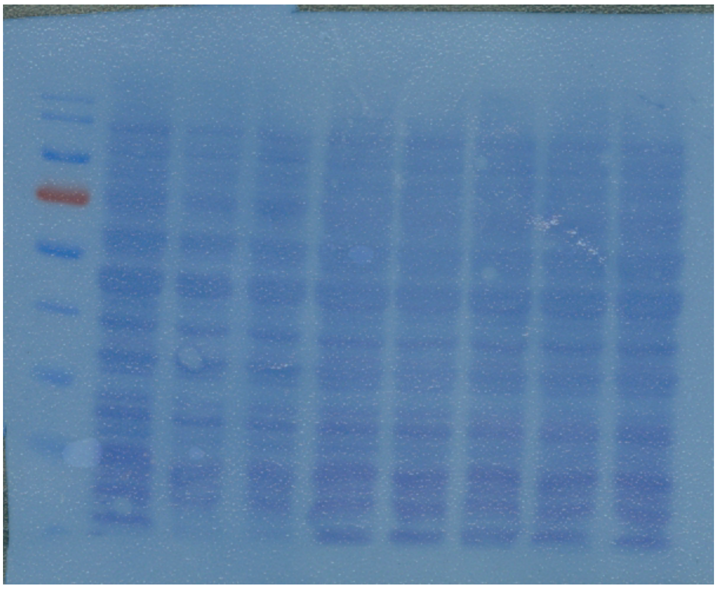

DB71 stain

Figure 5A - Cox5a

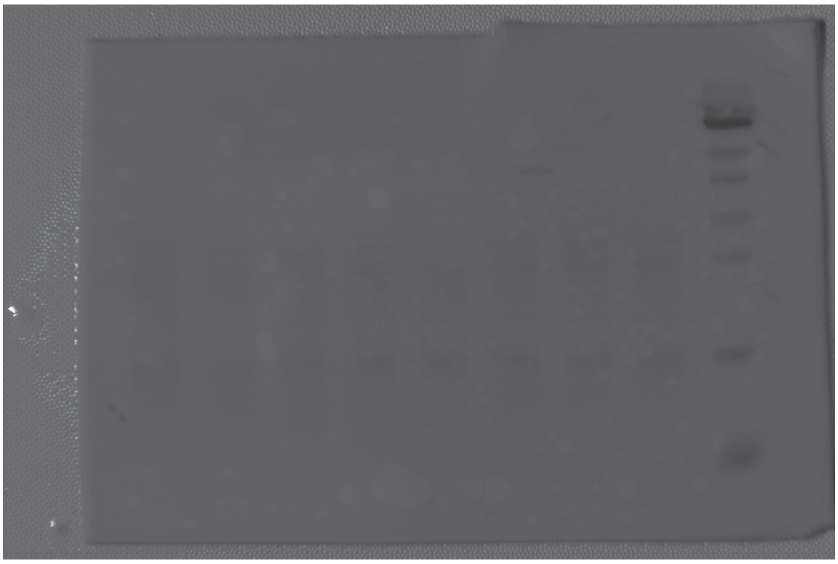

Protein ladder

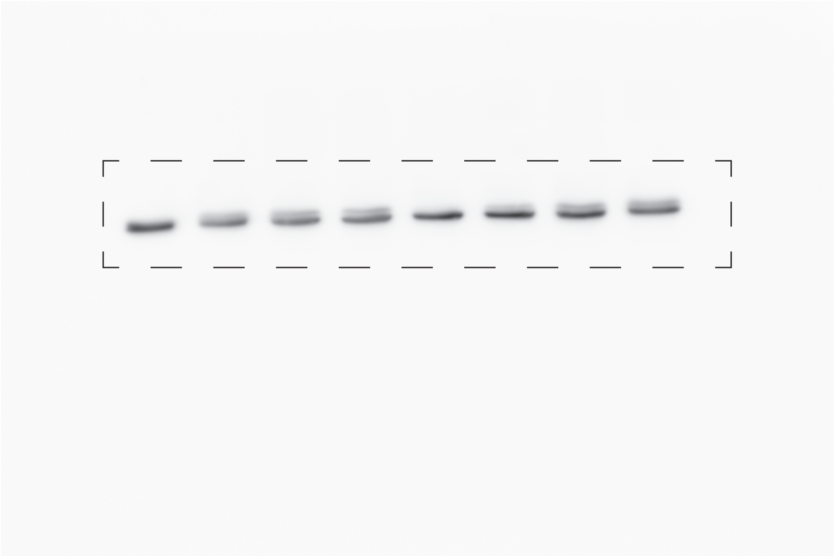

Relevant bands

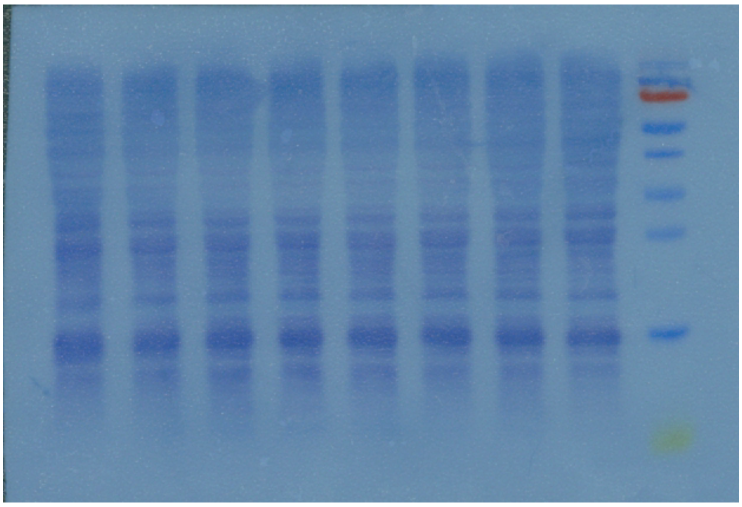

DB71 stain

Figure 5A - Ilv6

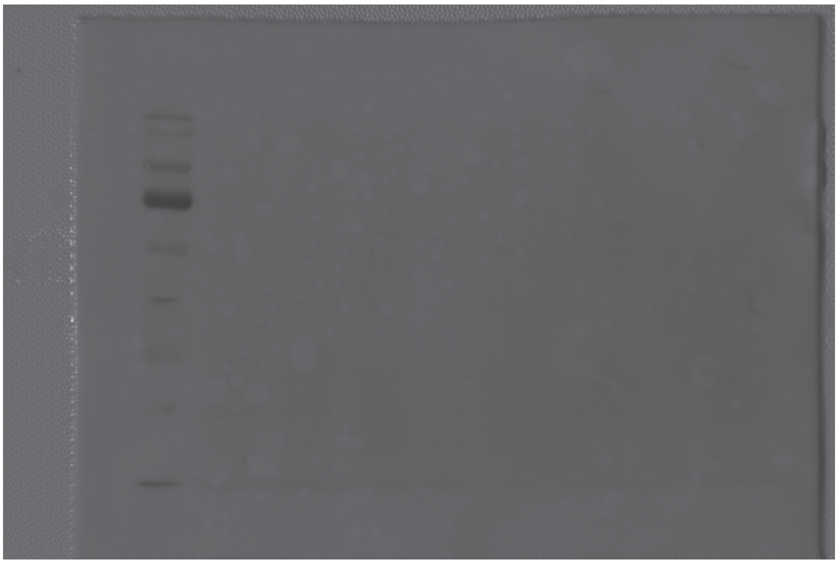

Protein ladder

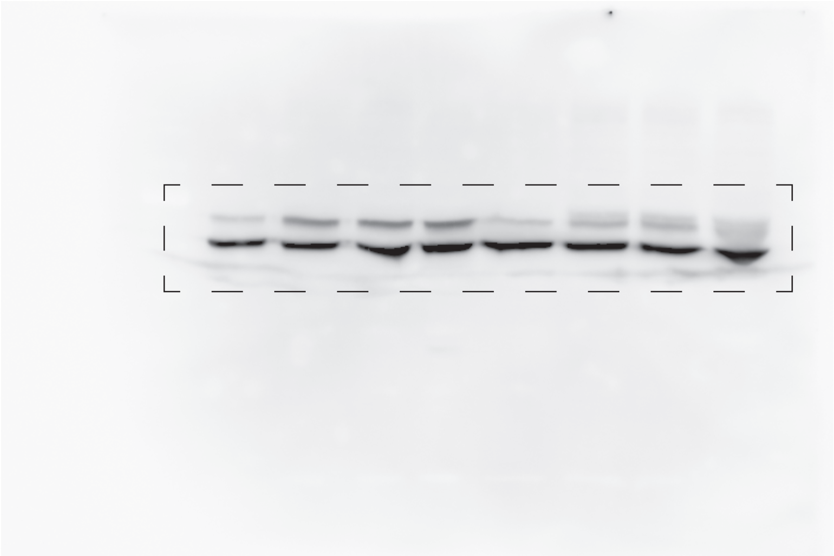

Relevant bands

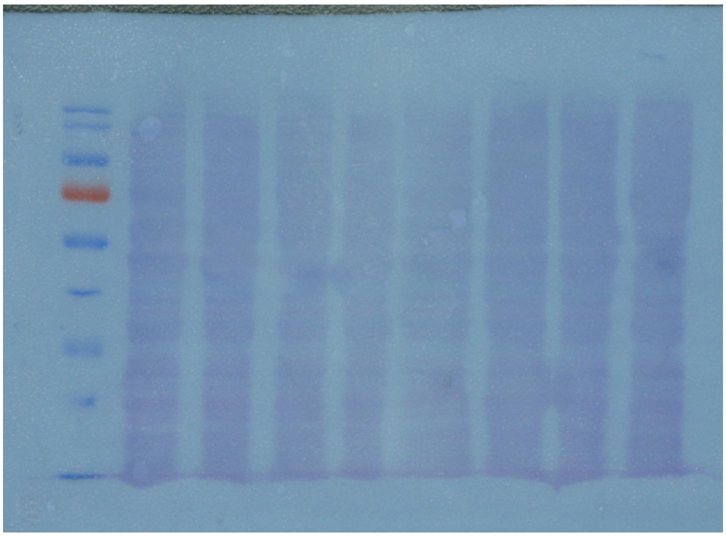

DB71 stain

Figure 5B - Aim17

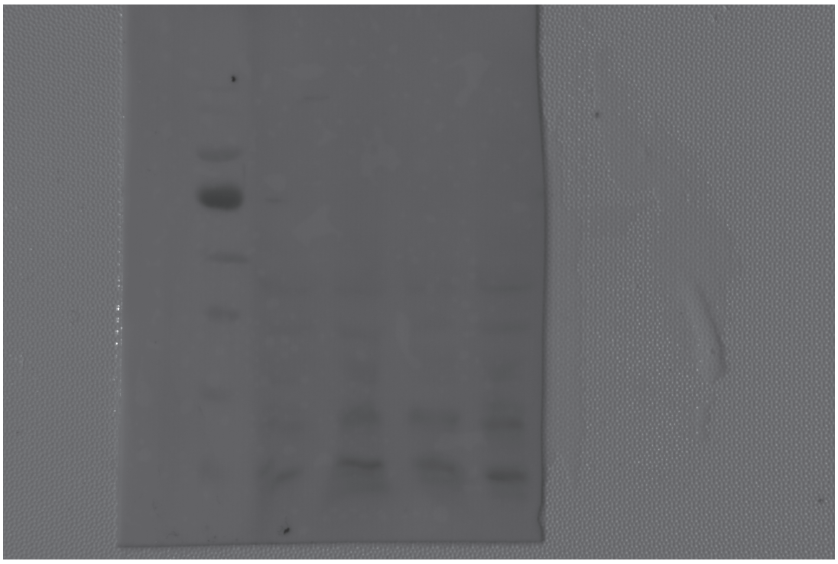

Protein ladder

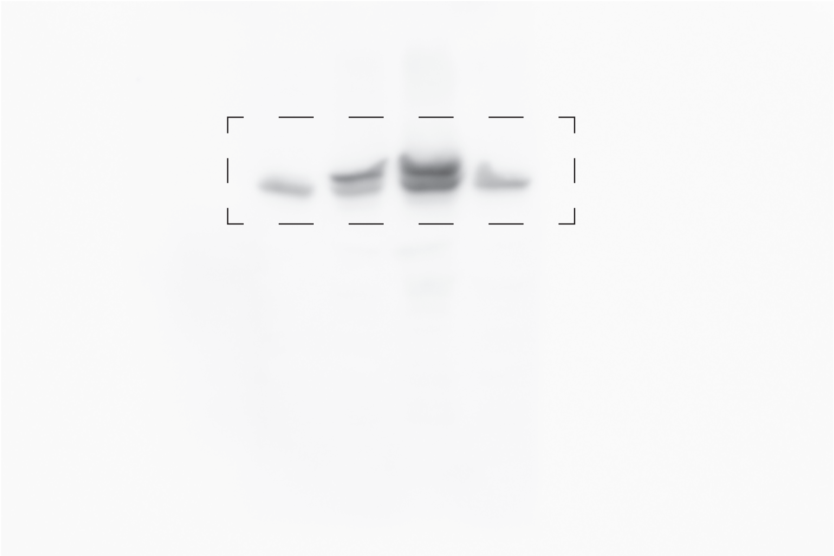

Relevant bands

Figure 5B - Cox5a

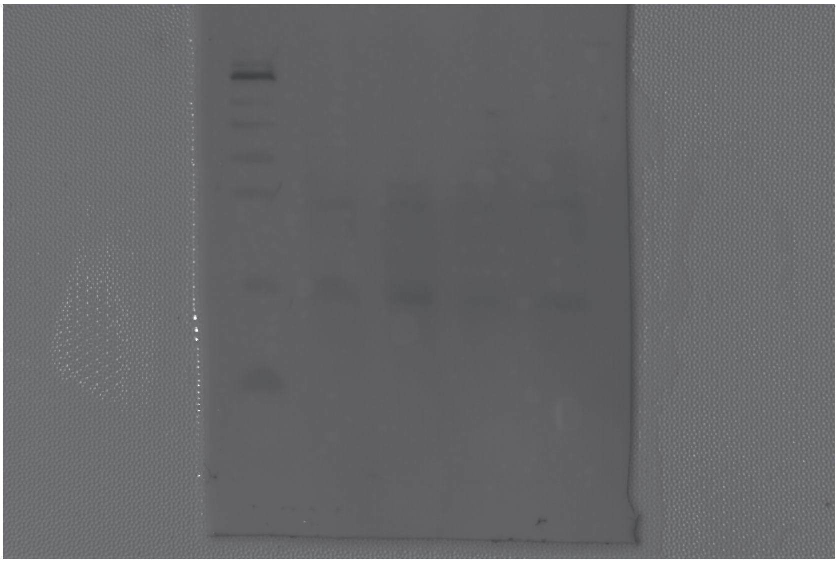

Protein ladder

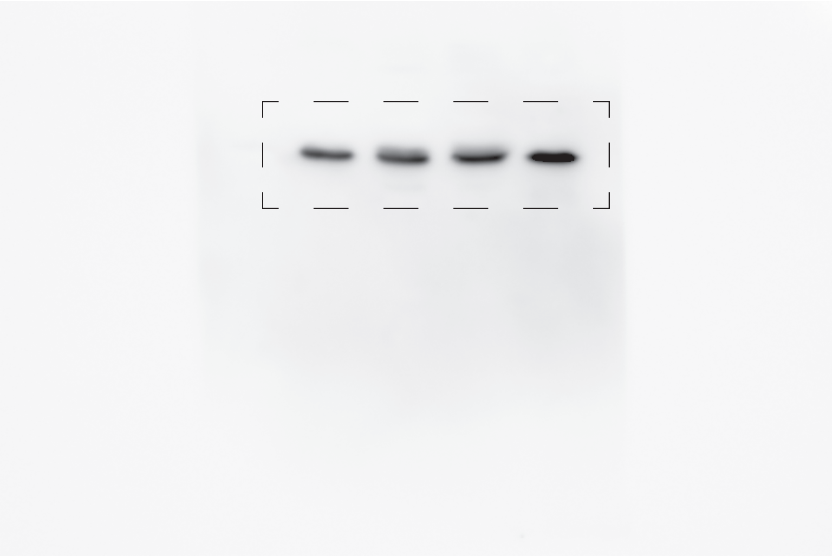

Relevant bands

Figure 5B - Ilv6

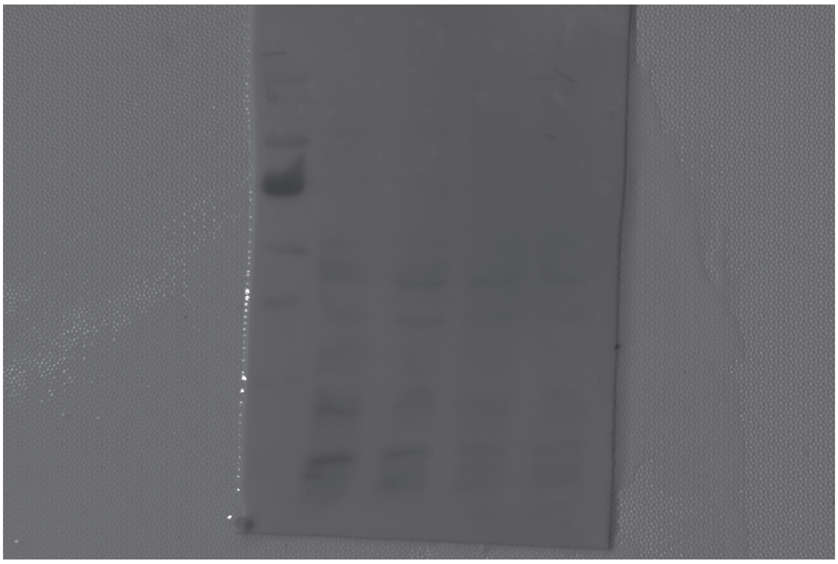

Protein ladder

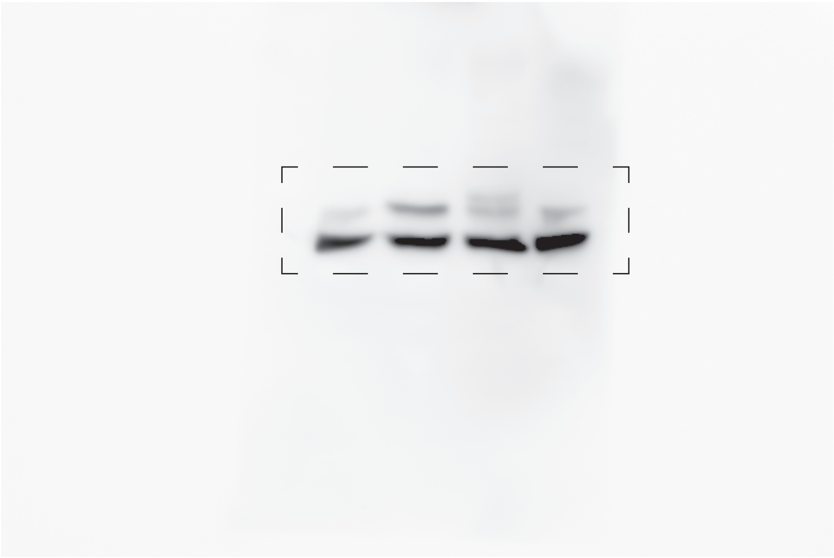

Relevant bands

Figure 5B - Sdh4

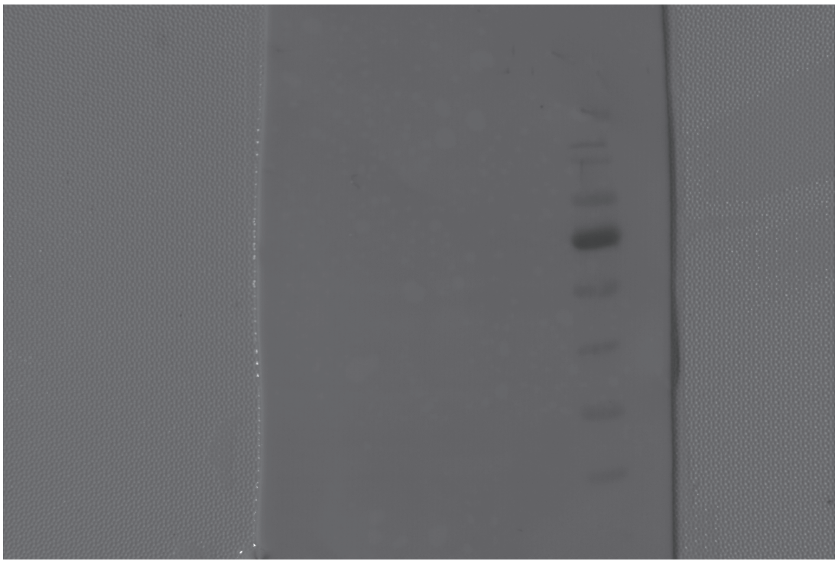

Protein ladder

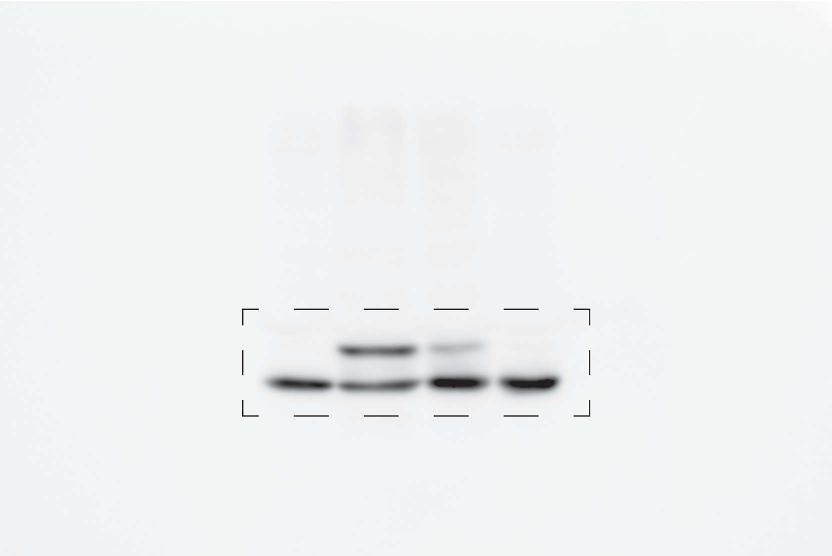

Relevant bands

Figure 5D - Aim17

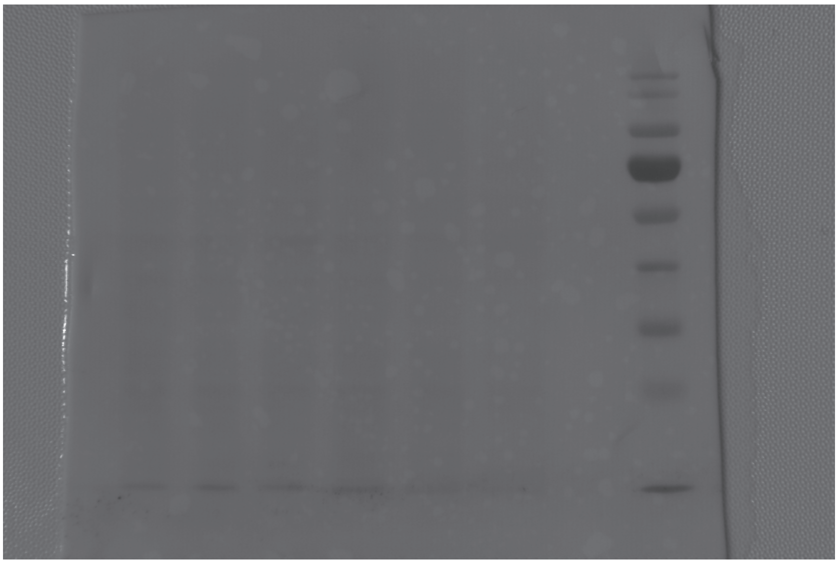

Protein ladder

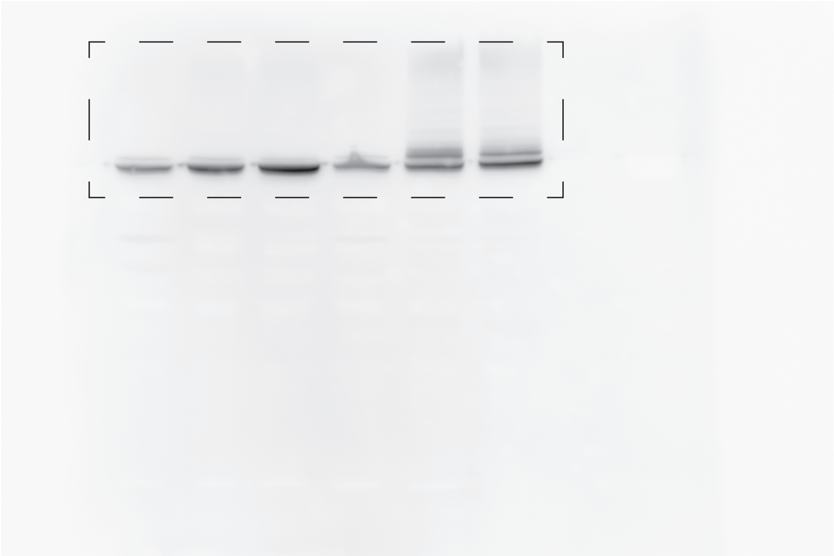

Relevant bands

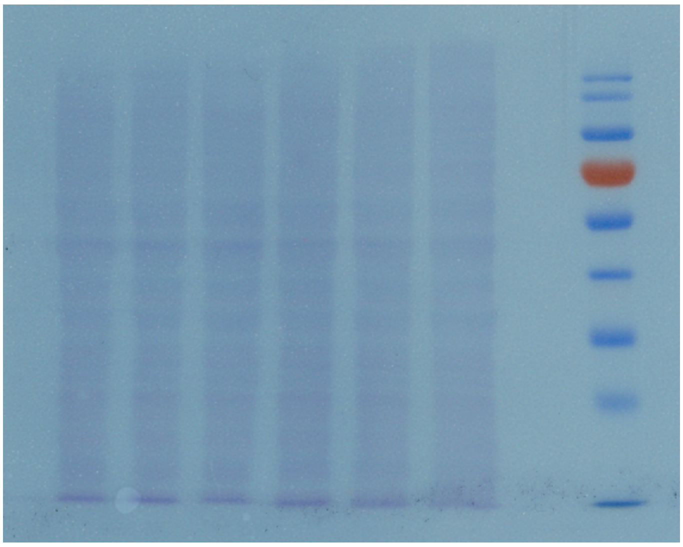

DB71 stain

Figure 5D - Cyc7

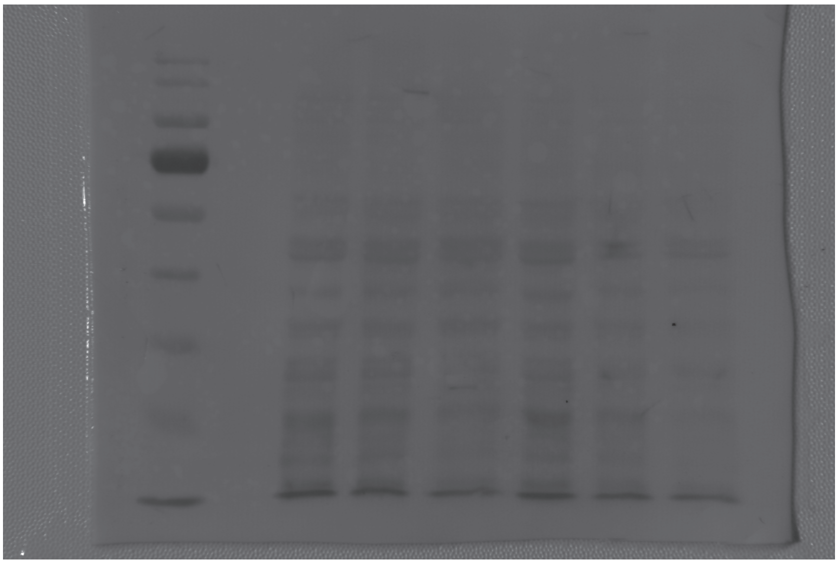

Protein ladder

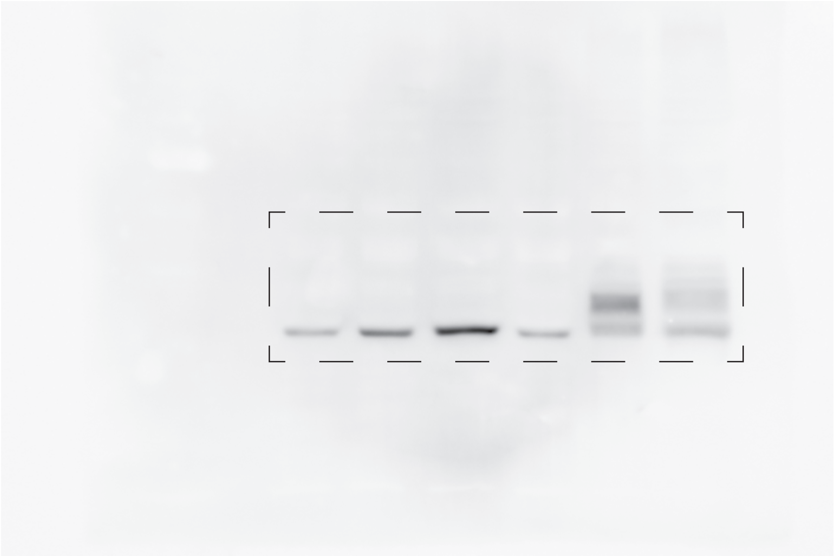

Relevant bands

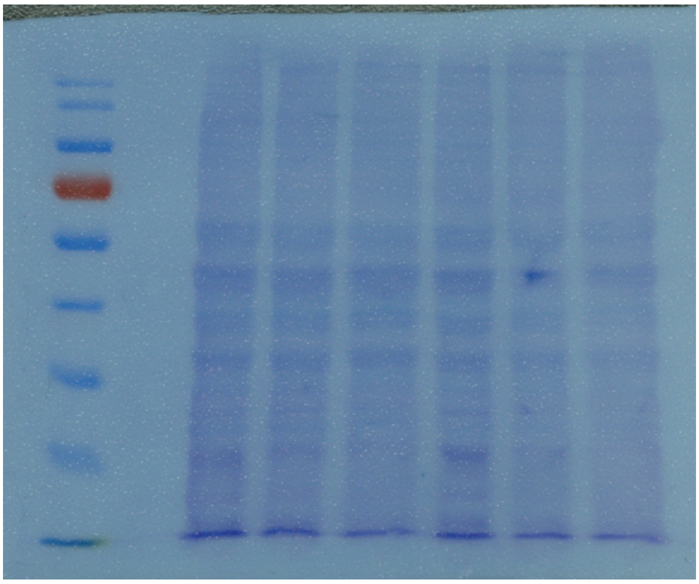

DB71 stain

Figure 5D - Sdh4

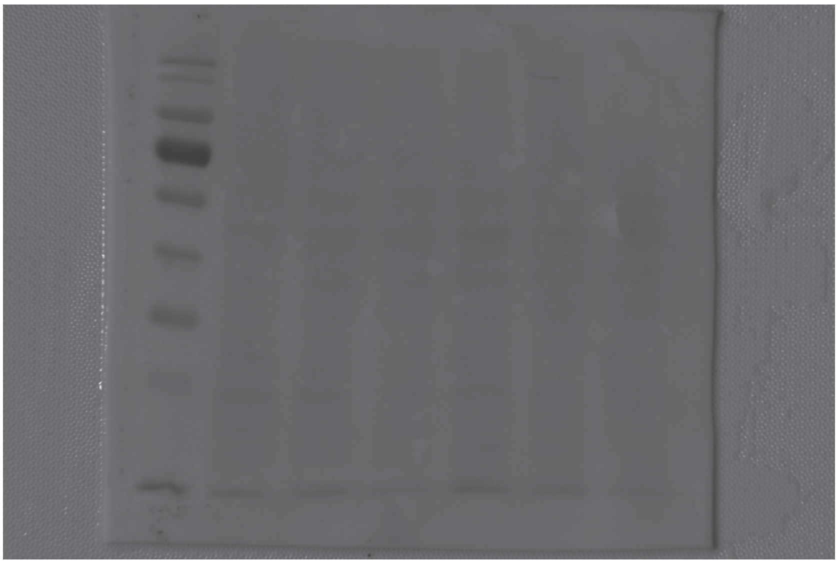

Protein ladder

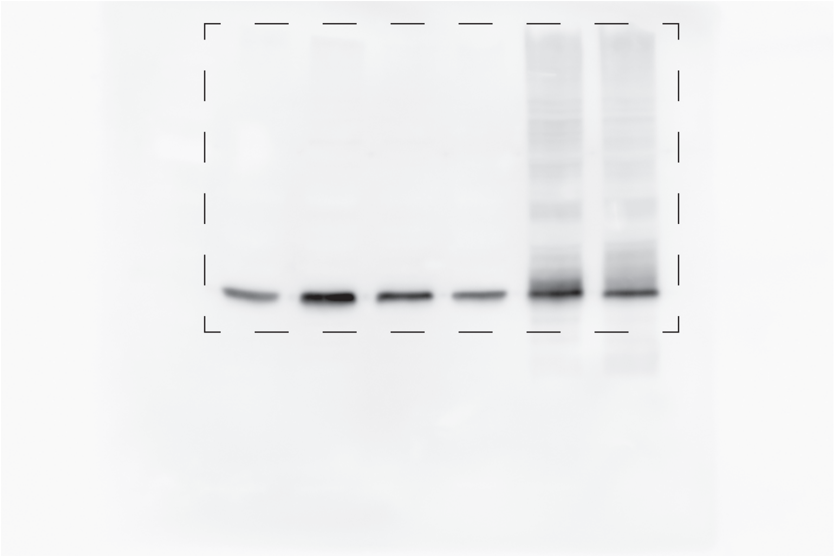

Relevant bands

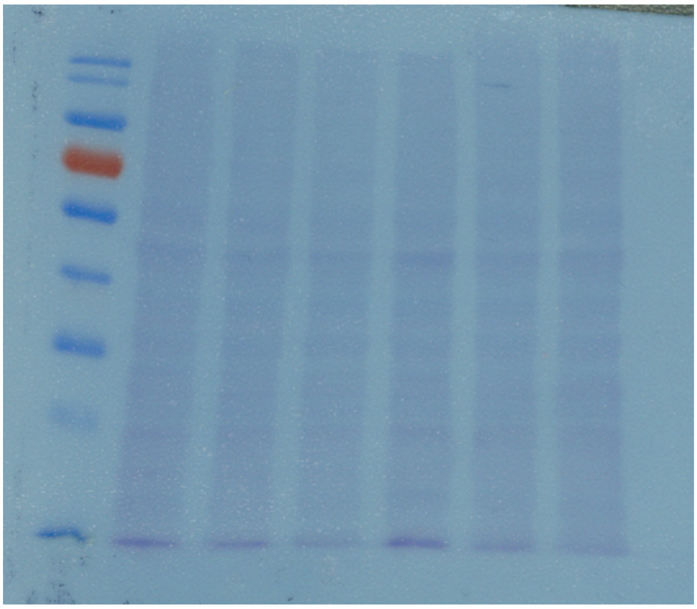

DB71 stain

Figure 5E - Aim17

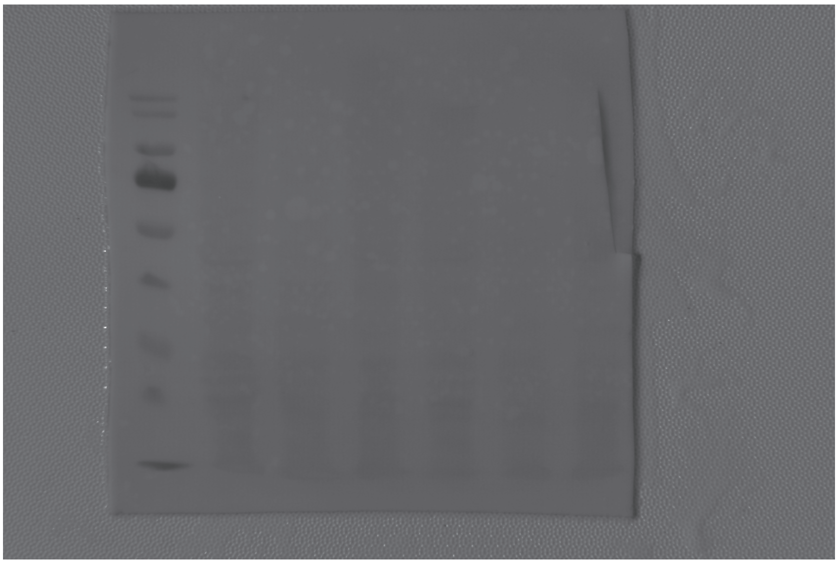

Protein ladder

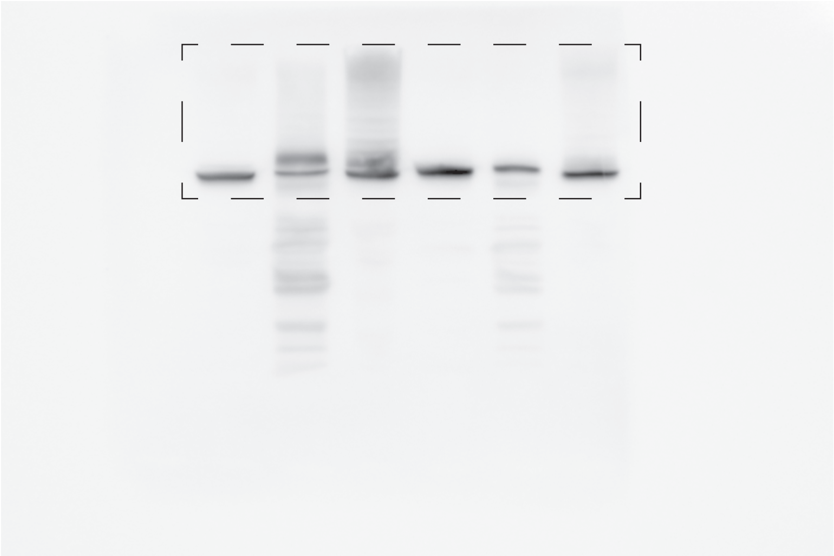

Relevant bands

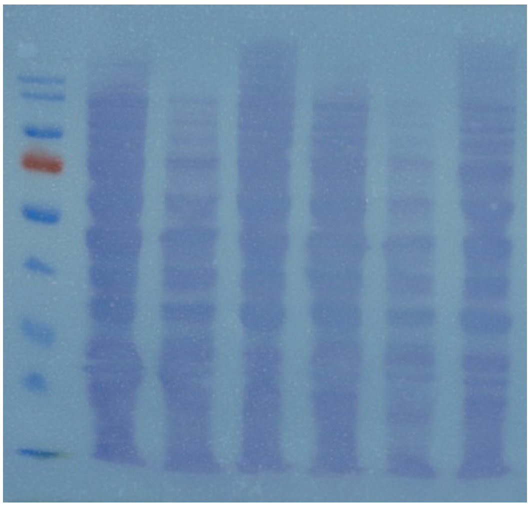

DB71 stain

Figure 5E - Mpc3

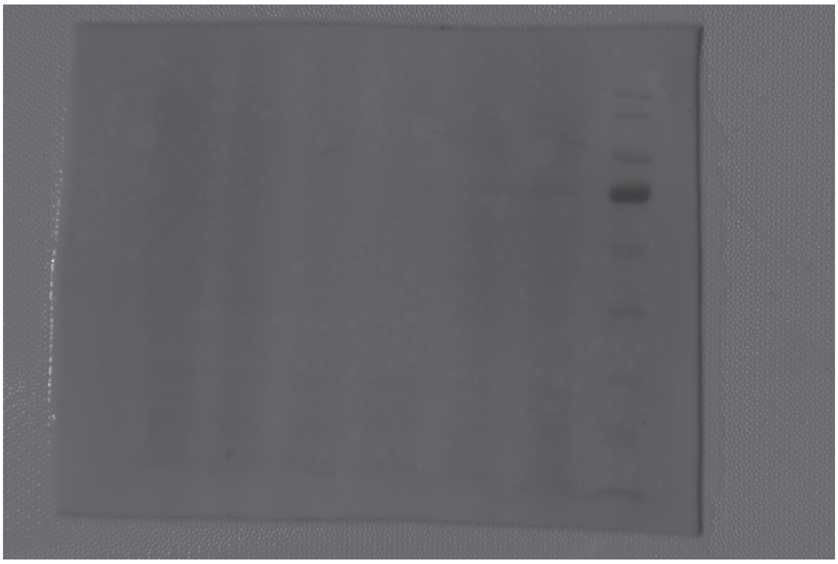

Protein ladder

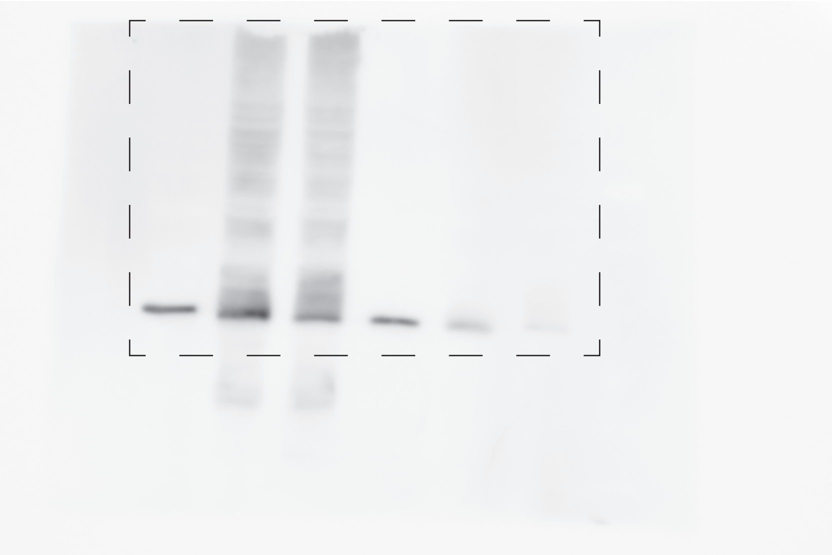

Relevant bands

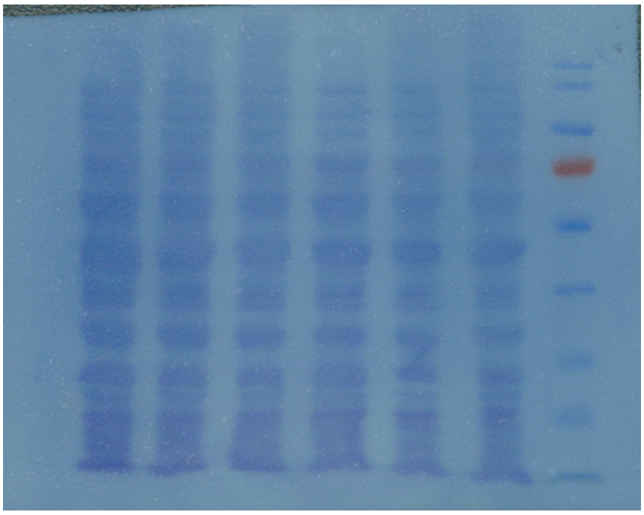

DB71 stain

Figure 5F - Cis1

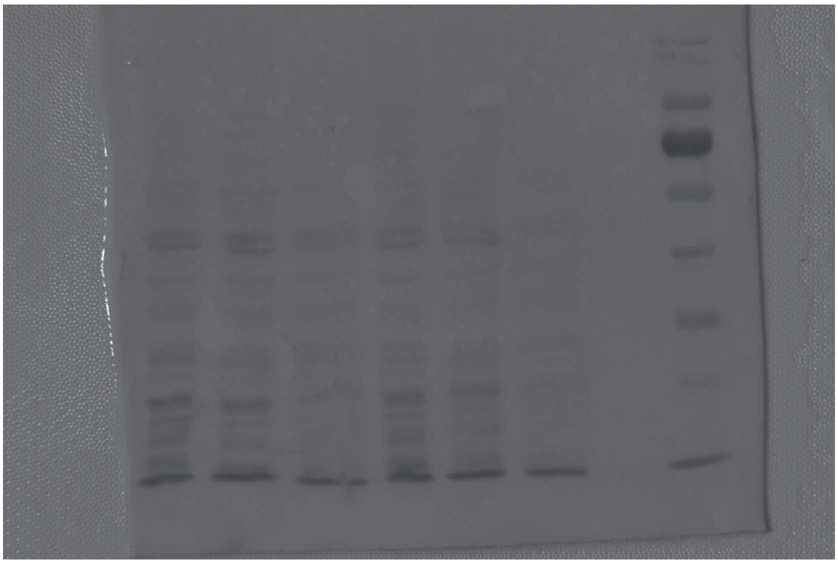

Protein ladder

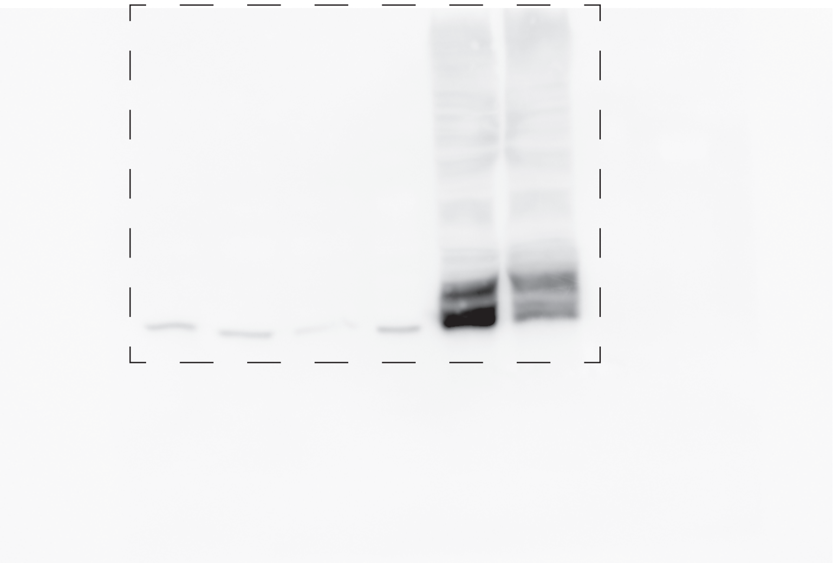

Relevant bands

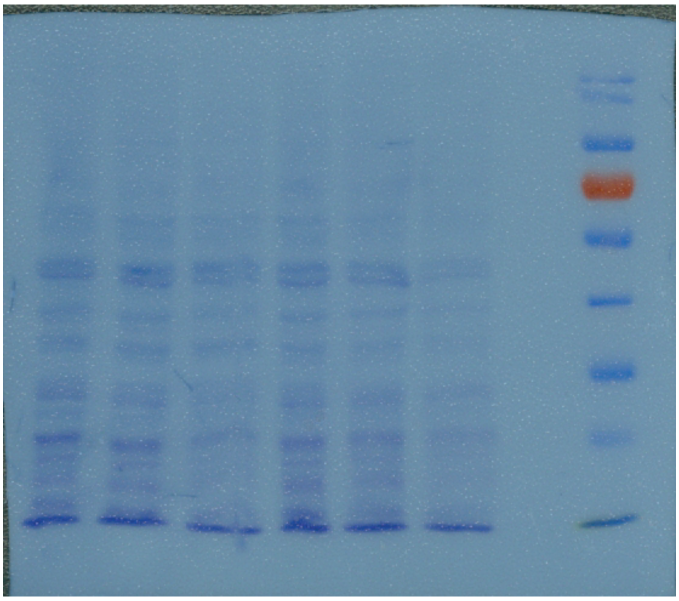

DB71 stain

Figure 5F - Tma10

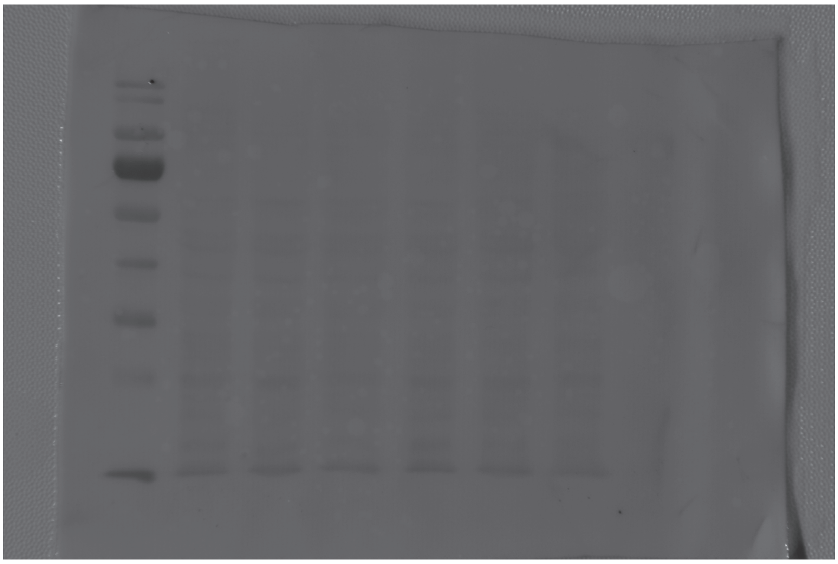

Protein ladder

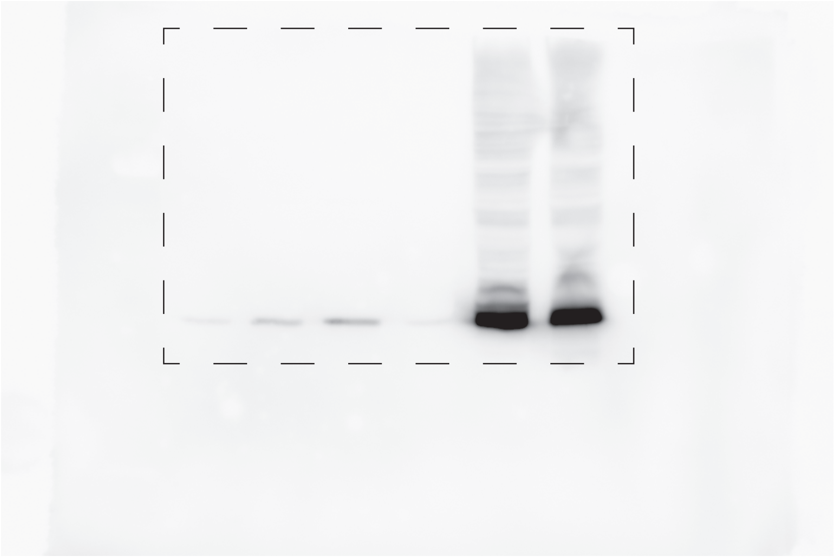

Relevant bands

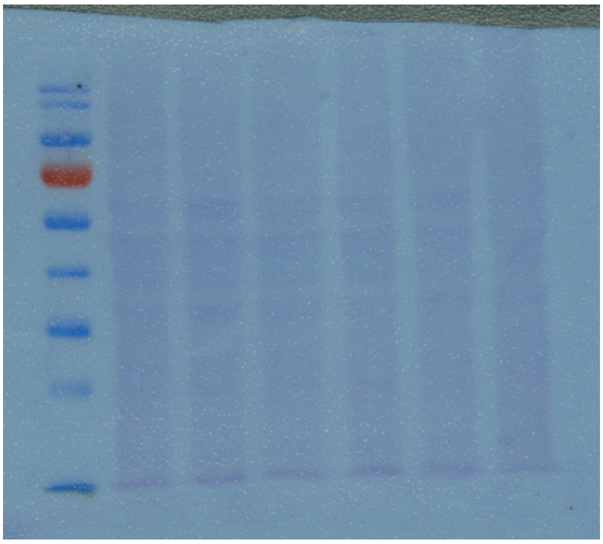

DB71 stain

Figure 5G - WT

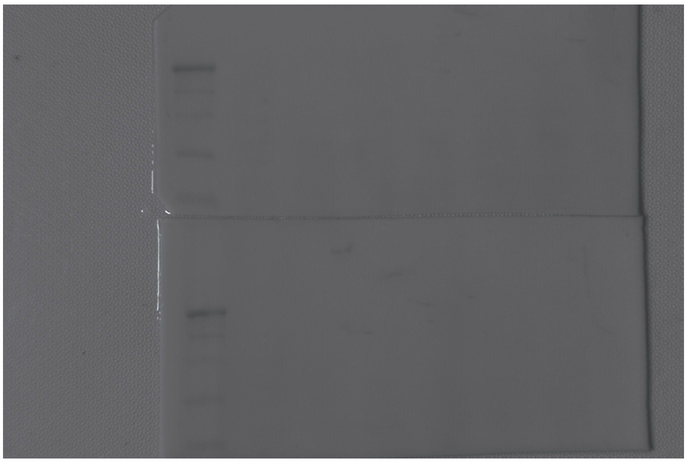

Protein ladder

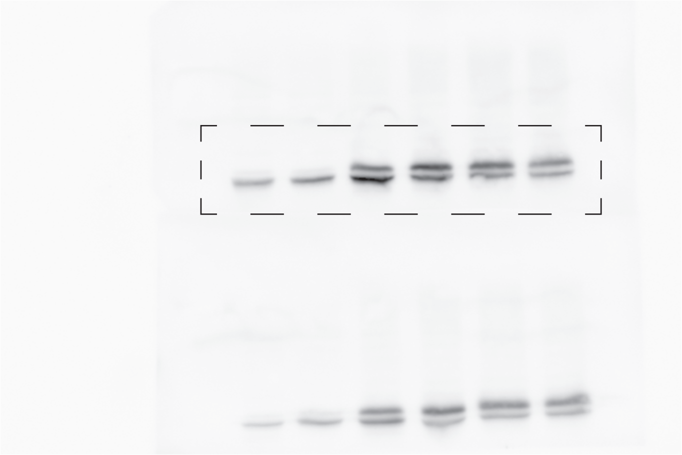

Relevant bands - TAP

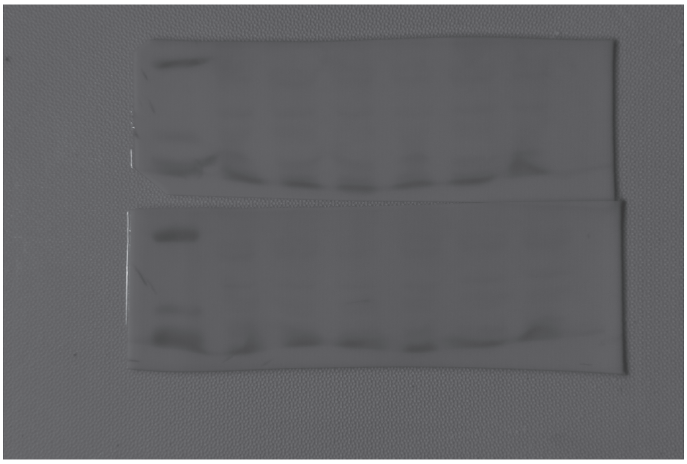

Protein ladder

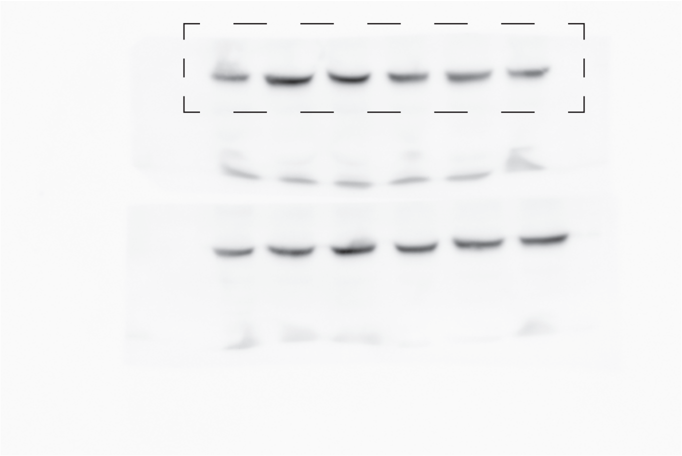

Relevant bands - Pgk1

Figure 5G - *hfd1*Δ

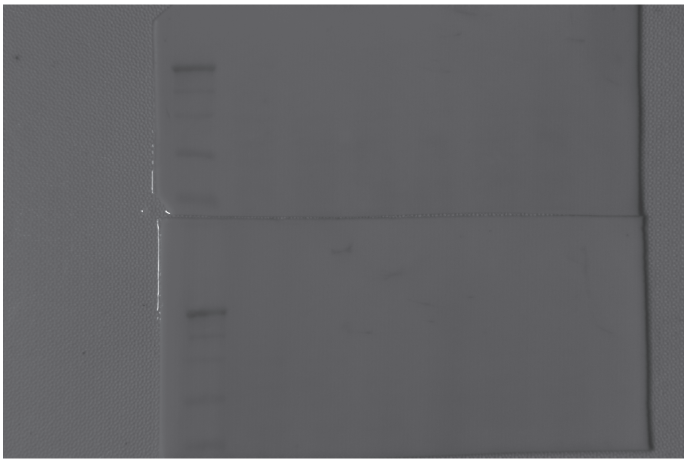

Protein ladder

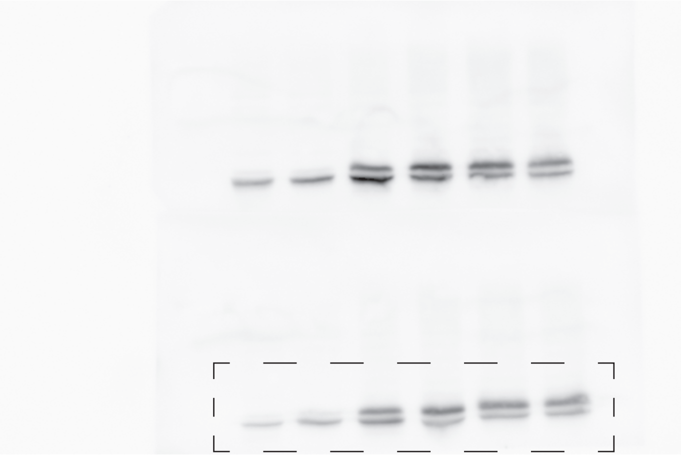

Relevant bands - TAP

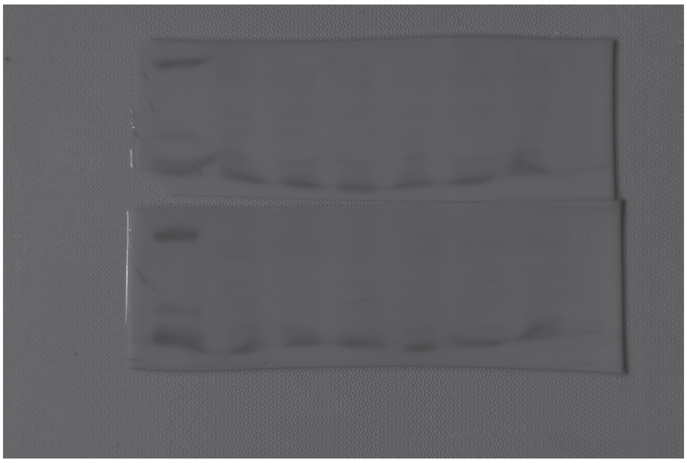

Protein ladder

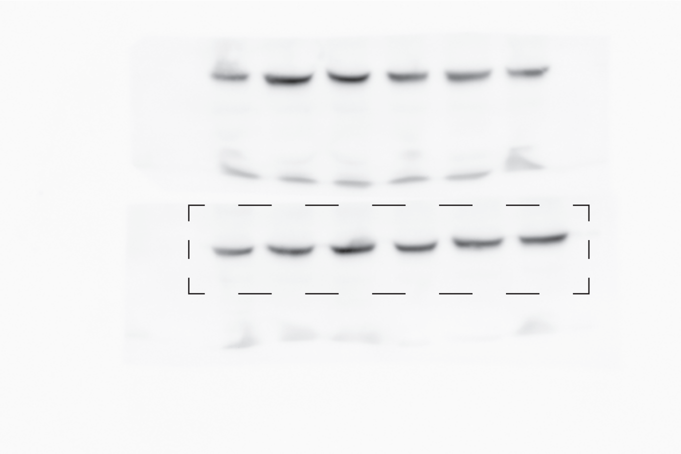

Relevant bands - Pgk1

Figure 5G - *TDH3*p-*HFD1*

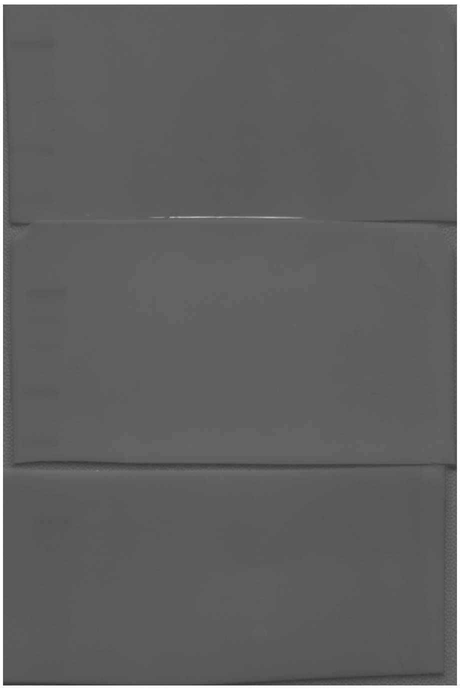

Protein ladder

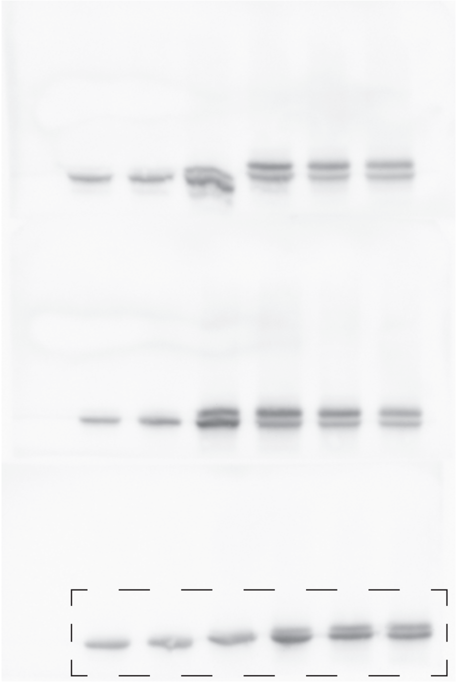

Relevant bands - TAP

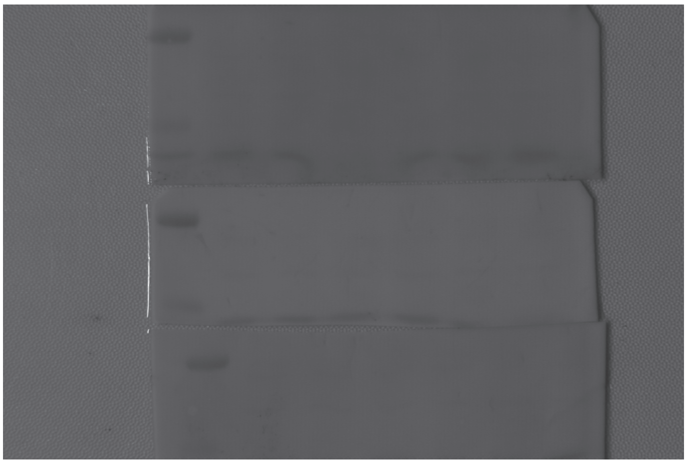

Protein ladder

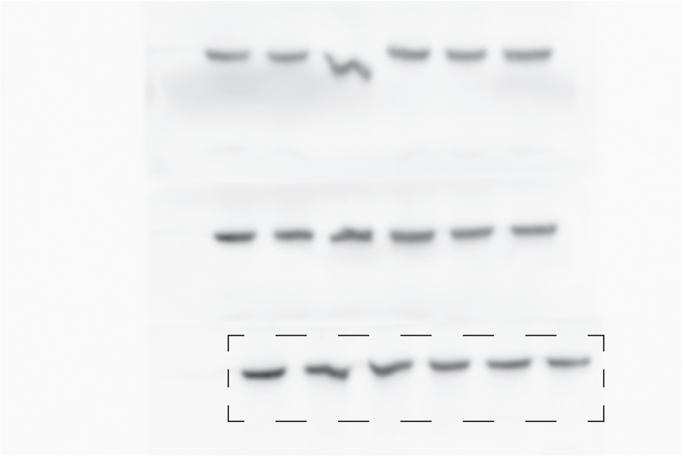

Relevant bands - Pgk1

Figure 5H

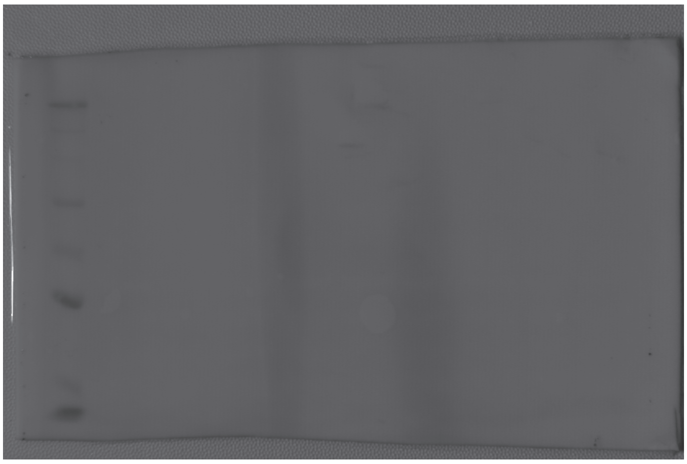

Protein ladder

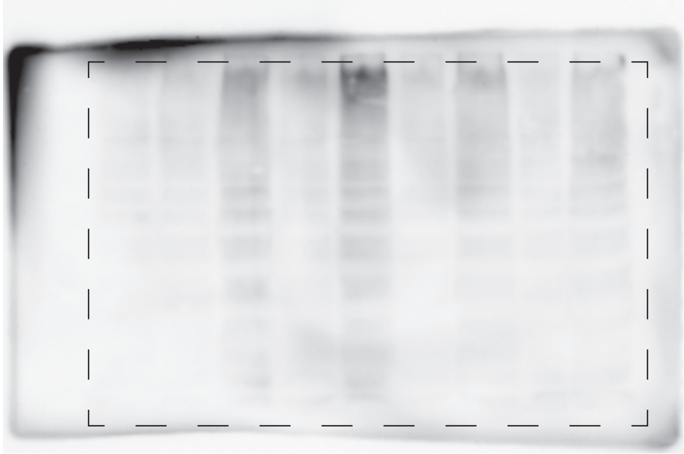

Relevant bands - TAP

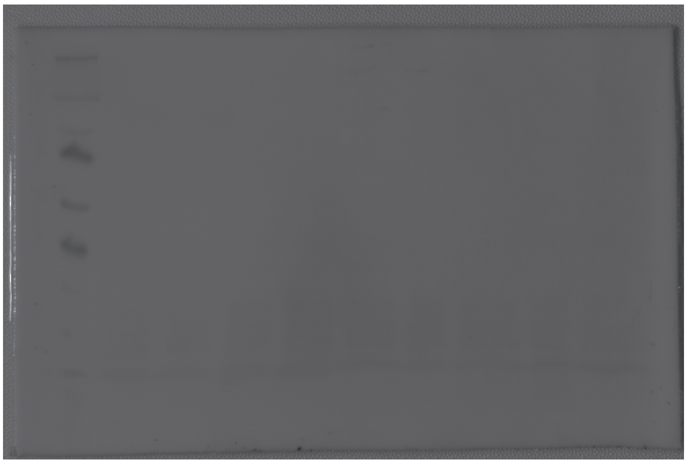

Protein ladder

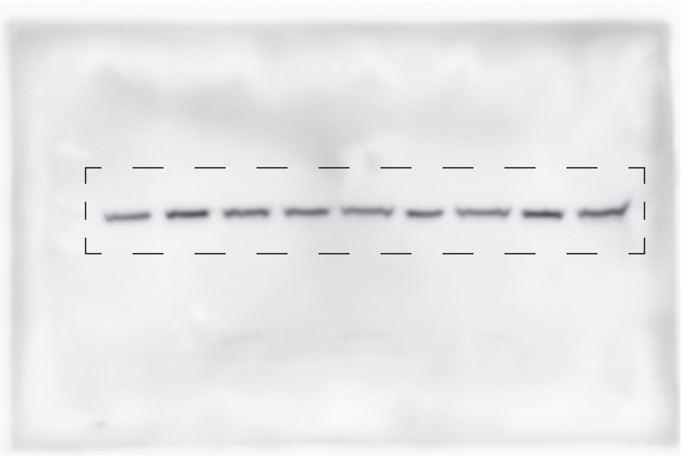

Relevant bands - Pgk1
