# Supplementary material for: Inhibition of mitochondrial protein import and proteostasis by a pro-apoptotic lipid
Source: eLife. 2025 May 30;13:RP93621. doi: 10.7554/eLife.93621 (PMC12124835; doi:10.7554/eLife.93621)

Figure 8A

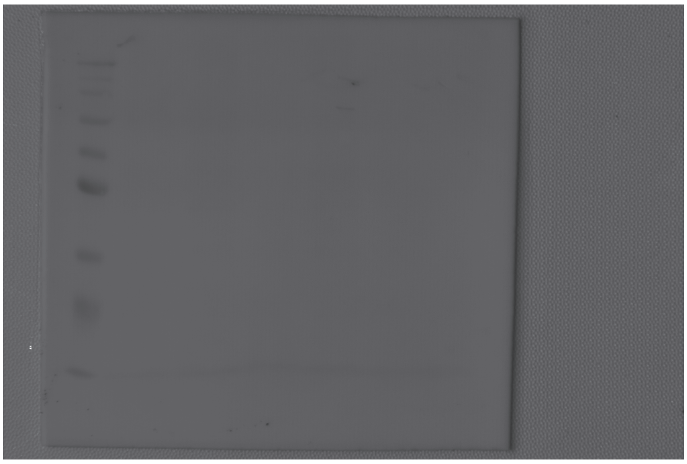

Protein ladder

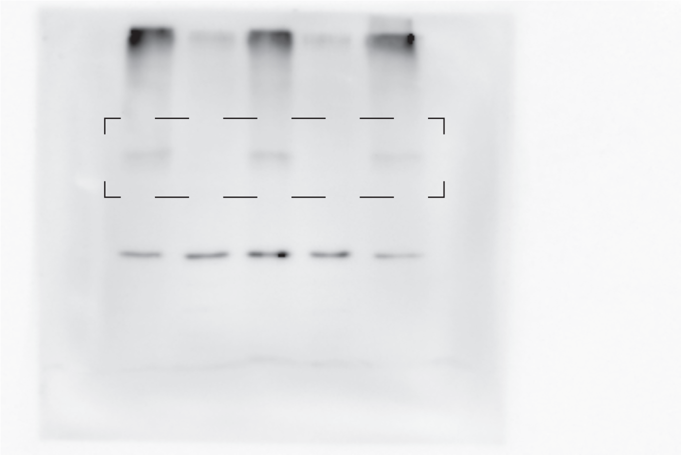

Relevant bands - Input HA

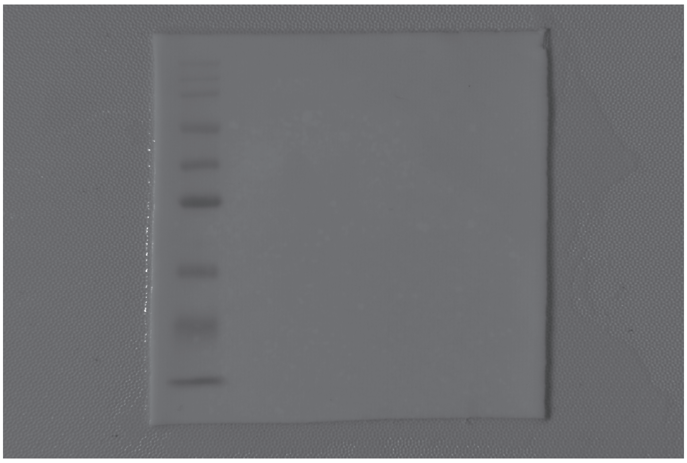

Protein ladder

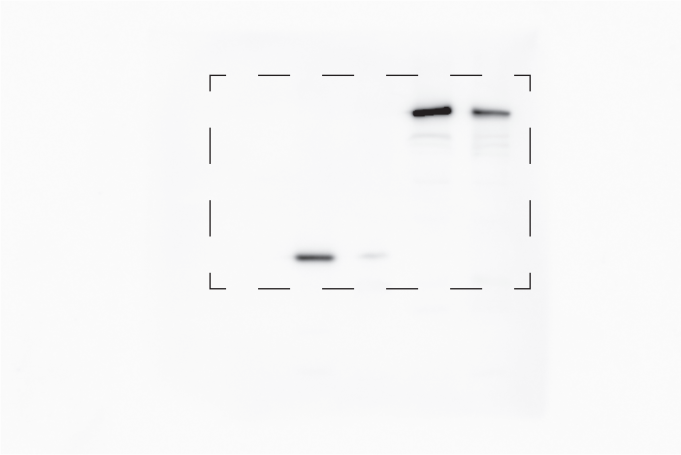

Relevant bands - Pulldown TAP

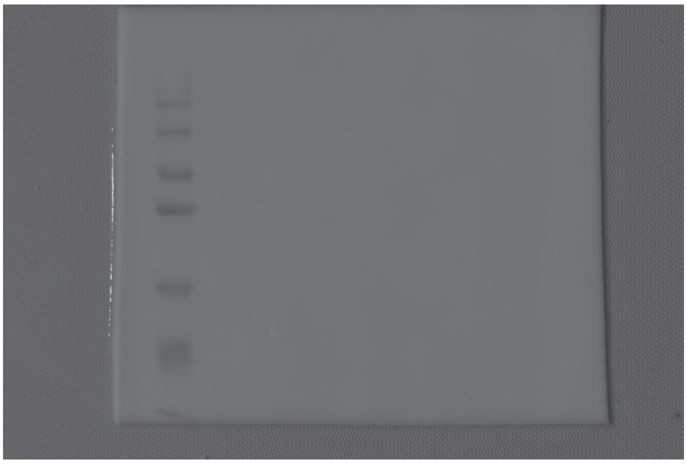

Protein ladder

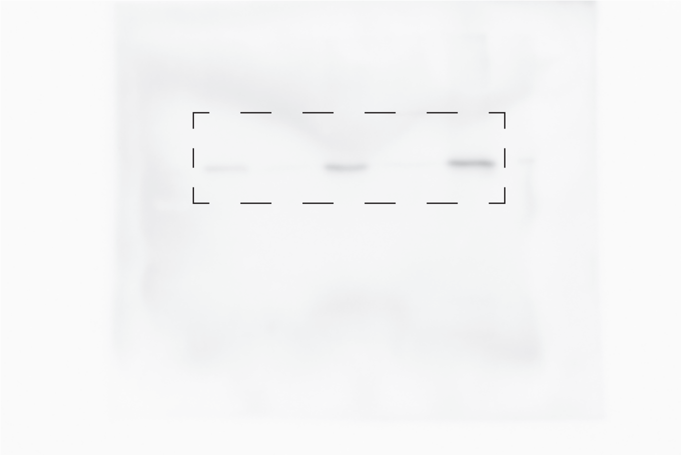

Relevant bands - Pulldown HA

Figure 8C - Upper panel

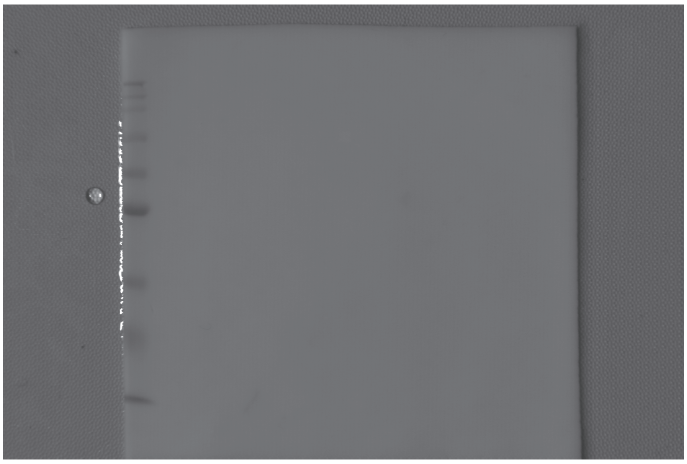

Protein ladder

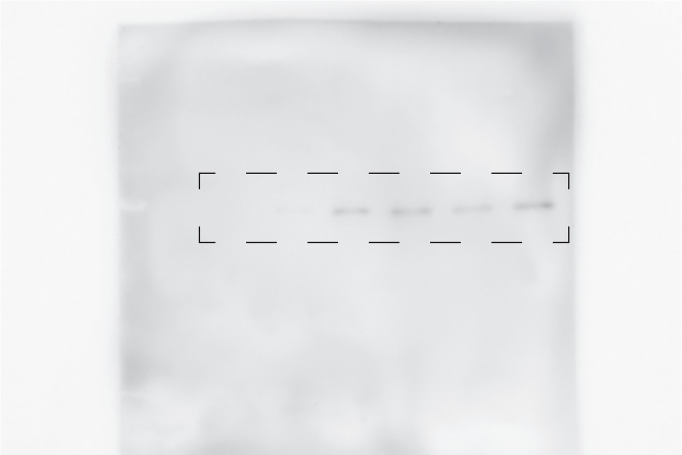

Relevant bands - Pulldown

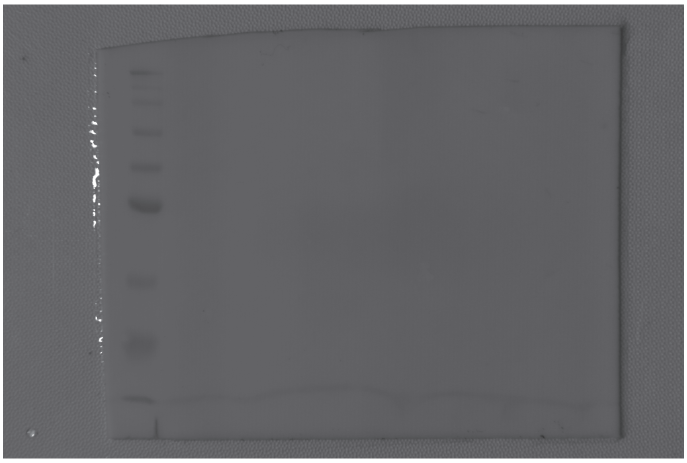

Protein ladder

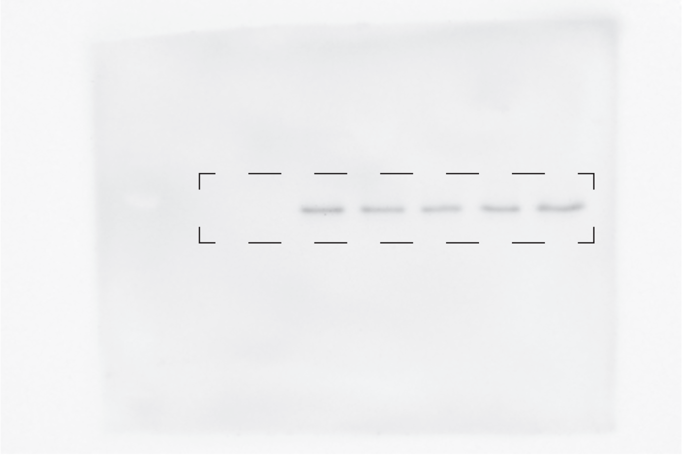

Relevant bands - Input

Figure 8C - Lower panel

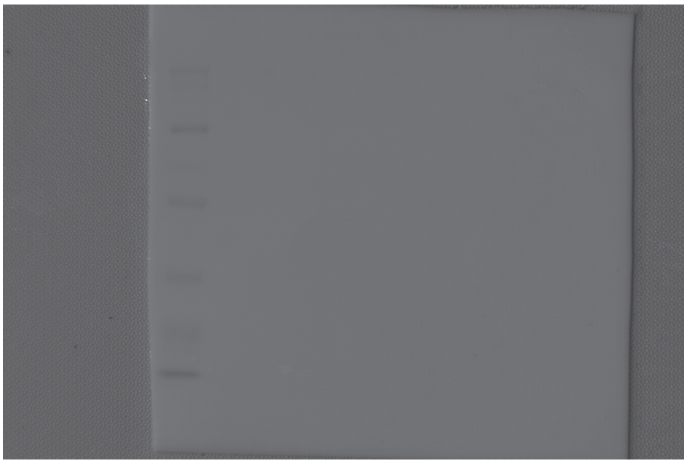

Protein ladder

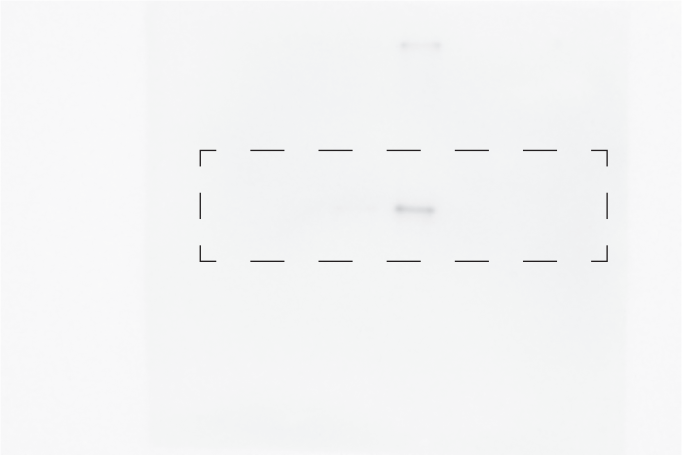

Relevant bands - Pulldown

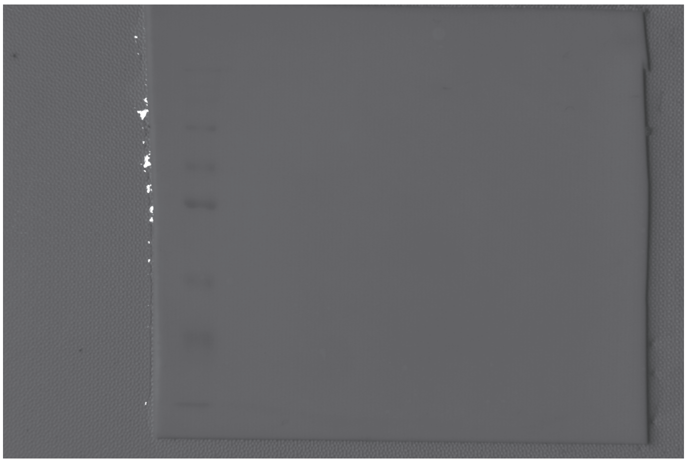

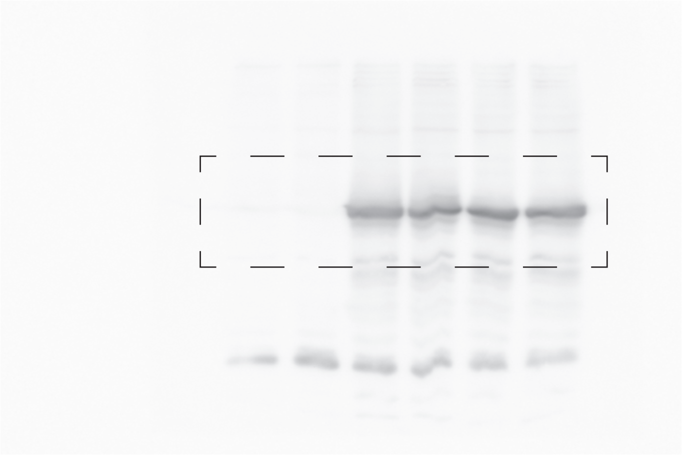

Supplement: Figure 8—source data 1. [file elife-93621-fig8-data1.pdf]
