# Supplementary material for: Inhibition of mitochondrial protein import and proteostasis by a pro-apoptotic lipid
Source: eLife. 2025 May 30;13:RP93621. doi: 10.7554/eLife.93621 (PMC12124835; doi:10.7554/eLife.93621)
Supplement: Figure 8—figure supplement 1—source data 1. [file elife-93621-fig8-figsupp1-data1.pdf]

Figure 8 - Figure Supplement 1 - WT

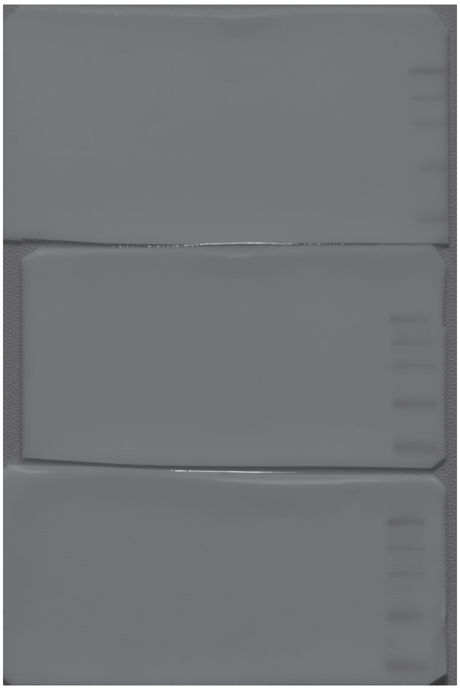

Protein ladder

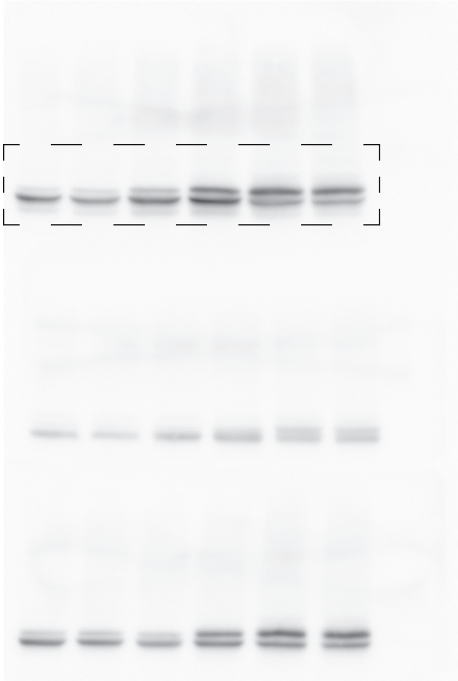

Relevant bands - TAP

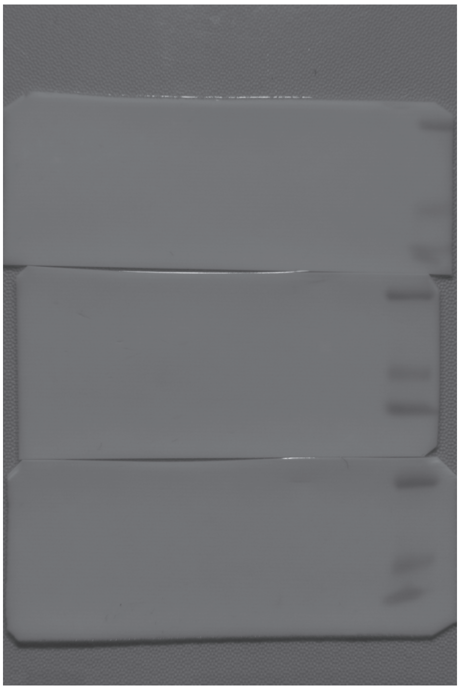

Protein ladder

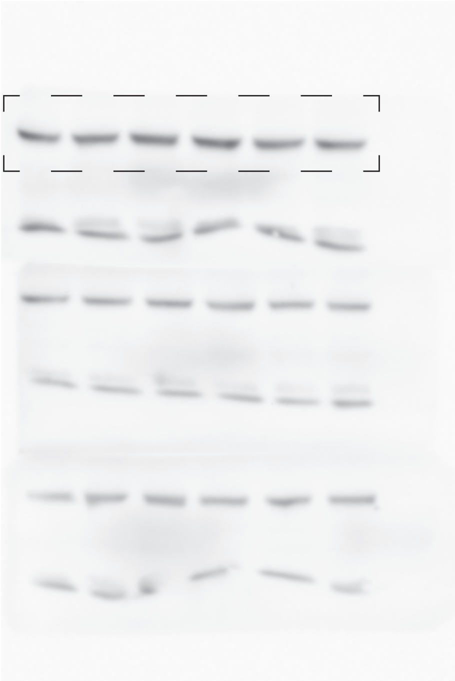

Relevant bands - Pgk1

Figure 8 - Figure Supplement 1 - *tom20*Δ

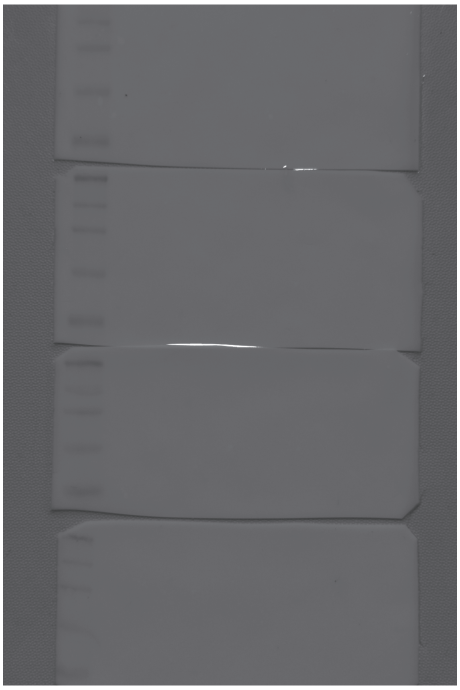

Protein ladder

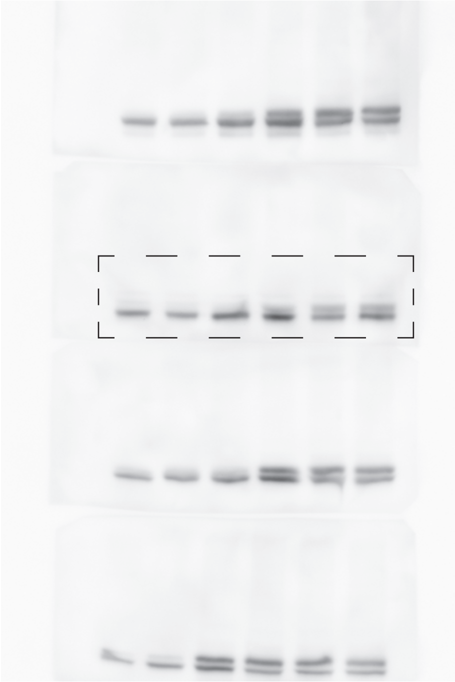

Relevant bands - TAP

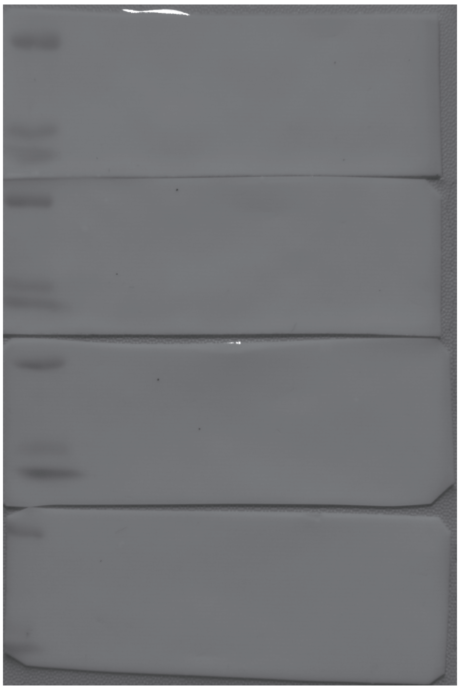

Protein ladder

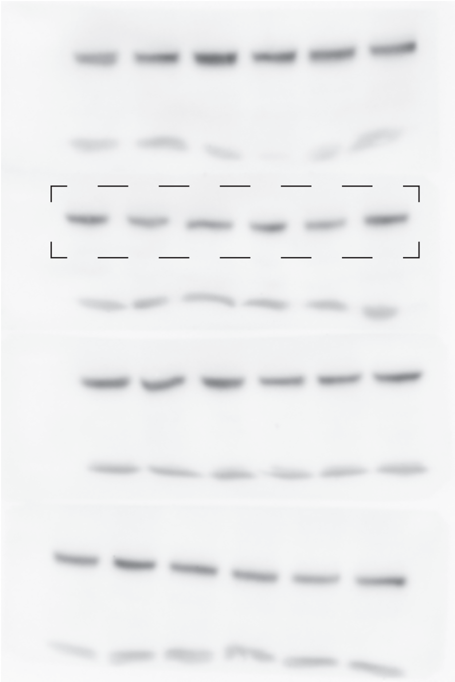

Relevant bands - Pgk1
